# Implementation of the SunSmart program and population sun protection behaviour in Melbourne, Australia: Results from cross-sectional summer surveys from 1987 to 2017

**DOI:** 10.1371/journal.pmed.1002932

**Published:** 2019-10-08

**Authors:** Tamara Tabbakh, Angela Volkov, Melanie Wakefield, Suzanne Dobbinson

**Affiliations:** Cancer Council Victoria, Melbourne, Australia; Peter MacCallum Cancer Centre, AUSTRALIA

## Abstract

**Background:**

Australia has one of the highest skin cancer rates in the world. ‘SunSmart’ is a multi-component, internationally recognised community-wide skin cancer prevention program implemented in Melbourne, Australia, since summer 1988–1989. Following recent reductions in melanoma rates among younger Australian cohorts, the extent of behaviour change and the potential contribution of prevention programs to this decline in melanoma rates are of interest. Sun protection is a multifaceted behaviour. Measures previously applied to monitor change over time in preventive behaviour for this population focused on individual behaviours. The omission of multiple behaviours that reduce exposure to ultraviolet radiation (UV) may have led to underestimates of behaviour change, meriting further analysis of long-term trends to contribute to this debate.

**Methods and findings:**

A population-based survey was conducted in Melbourne in the summer before SunSmart commenced (1987–1988) and across summers in 3 subsequent decades (1988–2017). During summer months, residents (14–69 years) were recruited to cross-sectional weekly telephone interviews assessing their tanning attitudes, sun protection behaviour, and sunburn incidence on the weekend prior to interview. Quotas were used to ensure the sample was proportional to the population by age and sex, while younger respondents were oversampled in some years. The majority of the respondents reported their skin was susceptible to sunburn. Changes in sun protection behaviour were analysed for *N =* 13,285 respondents in multivariable models, cumulating surveys within decades (1987–1988: *N =* 1,655; 1990s: *N =* 5,258; 2000s: *N =* 3,385; 2010s: *N =* 2,987) and adjusting for relevant ambient weather conditions and UV levels on weekend dates. We analysed specific and composite behaviours including a novel analysis of the use of maximal sun protection, which considered those people who stayed indoors during peak UV hours together with those people well-protected when outdoors. From a low base, use of sun protection increased rapidly in the decade after SunSmart commenced. The odds of use of at least 1 sun protection behaviours on summer weekends was 3 times higher in the 1990s than pre-SunSmart (adjusted odds ratio [AOR] 3.04, 95% CI 2.52–3.68, *p <* 0.001). There was a smaller increase in use of maximal sun protection including shade (AOR = 1.68, 95% CI 1.44–1.97, *p <* 0.001). These improvements were sustained into the 2000s and continued to increase in the 2010s. Inferences about program effects are limited by the self-reported data, the absence of a control population, the cross-sectional study design, and the fact that the survey was not conducted in all years. Other potential confounders may include increasing educational attainment among respondents over time and exposure to other campaigns such as tobacco and obesity prevention.

**Conclusions:**

With an estimated 20-year lag between sun exposure and melanoma incidence, our findings are consistent with SunSmart having contributed to the reduction in melanoma among younger cohorts.

## Abstract 2

Suzanne Dobbinson and colleagues reveal the benefits of the SunSmart program that has reduced rates of melanoma incidence in Melbourne, Australia.

## Author summary

**Why was this study done?:**

- In Australia, prevention programs have been implemented over 30 years encouraging the population to reduce their exposure to ultraviolet radiation (UV), the main cause of skin cancer.
- Prior research assessing the impact of these programs has focused on monitoring change in the prevalence of individual sun protection behaviours, and has largely overlooked sun avoidance and composite sun protection behaviours.
- This may have led to an underestimation of the behaviour change associated with these programs, given that effective sun protection involves multiple strategies.

**What did the researchers do and find?:**

- The prevalence of sun protection behaviour and sunburn incidence of residents of Melbourne, Australia, were analysed across 3 decades.
- Data were from a series of population-based cross-sectional surveys conducted in summers between 1987 and 2017.
- A considerably higher increase in sun protection behaviour than previously reported was revealed, with a rapid increase evident soon after the SunSmart program was established.

**What do these findings mean?:**

- The findings are consistent with the possibility that changes over the decades in sun protection behaviour have contributed to the decline in melanoma rates, and have substantial implications for the future delivery of skin cancer prevention programs.

## Introduction

Exposure to ultraviolet radiation (UV) is the main cause of skin cancer [1,2]. Melanoma is the most life-threatening form of skin cancer, while squamous cell carcinoma and basal cell carcinoma also present a substantial burden to public health [3,4]. In Australia the combined incidence of skin cancer is higher than the incidence of any other type of cancer and causes significant mortality (melanoma: 6.9 per 100,000 in 2016; squamous cell carcinoma and basal cell carcinoma combined: 1.9 per 100,000 in 2002) [5]. Australia’s high ambient UV environment and large population proportion with susceptible skin types account for the high incidence rates [6–8]. Skin cancer is a highly preventable disease, with an estimated 96% of melanoma cases in Oceania, and 75% globally, attributable to UV exposure [7]. To prevent skin cancer and other UV-related diseases, the World Health Organization recommends people protect their skin from the sun when the UV Index is 3 or higher [9].

Australia has implemented one of the most sustained and comprehensive suites of programs for skin cancer prevention [10–13]. Initial efforts were modest, relying on unpaid community service announcements on television and dissemination of brochures to raise awareness of skin cancer [11]. In 1988 the multi-component community-wide prevention program ‘SunSmart’ was established in the state of Victoria [12–14], and similar programs followed soon after in other Australian states [13]. These programs are widely acknowledged as being successful in raising public awareness, and promoting preventive behaviours and environmental change for skin cancer prevention [4,15]. In recent years a decrease in melanoma incidence and mortality among Australians born from about 1958 onward has prompted discussion about whether the extent of behaviour change achieved by these programs is sufficient to have contributed to these reduced melanoma rates [16–19]. In reviewing the evidence for behaviour change, one study labelled the extent of behaviour change as relatively ‘modest’, whilst widespread environmental supports for skin cancer prevention are posited as likely having a larger impact [17]. Certainly, during the past 40 years a large number of environmental supports for skin cancer prevention have been introduced in Australia. These supports include the widespread implementation of policies for hat wearing and shade provision in child care centres [20], primary schools [21], and workplaces [14]; the availability of more effective sunscreens that extend protection time and filter a greater range of UV rays [22]; the inclusion of sun protection items as a tax deductible expense for outdoor workers [23]; the increased availability from the 1980s of long-sleeved sun protective swimwear [24]; a ban on use of solaria in 2014 [25]; the provision of UV forecasts in weather reports [26]; and, in recent years, a comprehensive program of grants for community shade [27]. Indeed, rather than a competing influence, many of these environmental changes are a direct result of the advocacy and awareness raising of skin cancer prevention campaigns and programs [14]. However, environmental change is of little benefit without behaviour change. Sustained public education and environmental support are seen as critical to achieving this [28].

Long-term assessment of the population’s sun protection behaviour in Melbourne, Victoria, has provided some evidence of the impact of the SunSmart program [28–31]. However, in these studies assessment of impact focused on the uptake of individual sun protection behaviours, and found that ‘at best’ less than half the population used any given behaviour on summer weekends, with the exception of covering clothing on the legs [29,31]. The focus of these prior studies on use of specific behaviours during activities outdoors on summer weekends is limiting, and may substantially underestimate the behaviour change for this population.

Five sun protection behaviours (using wide-brimmed hats, sunglasses, sunscreen, and covering clothing and staying under shade while outdoors) are promoted by SunSmart, and since each behaviour offers protection from UV exposure, combined efforts need to be considered. Moreover, one of the most effective methods of reducing UV exposure, namely minimising time spent outside during peak UV hours (i.e., 11 AM to 3 PM DST), has not to date been considered in estimating the prevalence of sun protection behaviour. A more nuanced analysis of behaviour change that addresses these gaps in assessing program impact is warranted, including data from the most recent surveys in the context of long-term patterns of change over time.

## Methods

Cancer Council Victoria conducted surveys of Melbourne residents’ sun-related attitudes and behaviour over 3 decades during a total of 14 summers between 1987 and 2017. Respondents were recruited to telephone interviews on a weekly cross-sectional basis over 12 to 13 weeks. Eligible respondents were aged 14 to 69 years and recruited via calls made to a random selection of landline telephones and, since 2013, mobile telephones. Verbal consent to participate in the study was sought from respondents, and from guardians of respondents aged 14 to 15 years, following a brief description of the study. The respondents were informed that ‘Today we’re doing a survey about people’s attitudes towards being out in the sun, and we’d like the opinion of people aged 12 to 69’. If the respondent or guardian did not consent, they were thanked for their time and the telephone call was terminated. Weekly demographic quotas were used to retain the desired sample. The study was approved by Cancer Council Victoria’s Human Research Ethics Committee (HREC 0018).

### Measures

A core set of identical questions, with established face and internal validity [32,33], were included in all survey years. Briefly, measures assessed respondents’ preference for a suntan/tanning attitudes, sun-related behaviour, and sunburn experience during activities while outdoors on the previous weekend during peak UV hours (11 AM to 3 PM DST). Behaviour was reported separately for the Saturday and Sunday, or occasionally for a Sunday and public holiday Monday, immediately preceding the interview. Details of questions from 2 survey years are provided in S1 and S2 Appendices.

Interviews were held within 2 to 4 days of the reported behaviour to minimise recall bias. The behavioural measures that focused on a specific time and location enabled linkage with weather and UV records for Melbourne on the dates relevant to the respondents’ reported behaviour. The specific data appended were (i) the 3 PM temperature and the mean UV Index between 11 AM and 3 PM (DST) for the weekend date and (ii) the number of days of rainfall (≥1 mm), number of days of heavy cloud cover (≥6 oktas), and median 3 PM temperature over the past month.

### Dependent variables

Eleven dependent variables from the survey responses were the focus of the main analysis: (i) likes to get a suntan, (ii) agrees a suntanned person is more healthy, (iii) agrees most of their friends think a suntan is a good thing, (iv) outdoors during peak UV on Sunday, (v) wore a hat, (vi) used sunscreen, (vii) stayed mostly in the shade, (viii) used at least 1 sun protective behaviour, (ix) maximal sun protection both days, (x) maximal sun protection both days including shade, and (xi) burnt either day.

Preference for a suntan was assessed by asking ‘Do you like to get a suntan or not?’ with responses ‘yes’ or ‘no’. Other tanning attitudes were assessed using a 5-point Likert scale and later recoded into dichotomous variables, to indicate respondents’ agreement or disagreement with 2 statements: ‘A suntanned person is more healthy’ and ‘Most of my friends think a suntan is a good thing’. The categorisation of values was not based on the distribution of responses but rather on theoretical considerations to characterise respondents as having protective versus unprotective attitudes.

Respondents’ reports of their behaviour during activities outdoors and sunburn experience were coded into dichotomous or continuous dependent variables for each reported day. Subsequently, ‘weekend’ variables were created primarily using responses for the most recent day (Sunday), unless the respondent was only outdoors on the other day (Saturday). The reports on public holiday dates were coded into the weekend variables using a similar approach. The dependent variables were coded as follows: (i) sun exposure, categorised as outdoors for 15 minutes or more during peak UV hours (yes or no) and time spent outdoors (minutes); (ii) the type of protection worn/used during peak UV hours, categorised as a hat (yes or no), covering tops, i.e., at least three-quarter-length sleeves (yes or no), protective leg cover, i.e., at least three-quarter-length clothing (yes or no), and sunscreen of at least SPF 12 (yes or no) (for consistency in SPF of maximal protection sunscreen products available across the survey years); (iii) stayed mostly in the shade during their main activity outdoors in peak UV hours (mostly shaded or equally/mostly out in the open); and (iv) sunburnt on either weekend day (yes or no). The details of questions and responses for these dependent variables are provided in S1 Table.

Three composite measures were derived considering all the protective behaviours used by respondents. First, a ‘body cover index’ from 0 to 1, indicating the extent of the body protected from the sun, was calculated for those respondents outdoors during peak UV hours on the weekend. The index was determined considering the proportion of cover to each part of the body as indicated by respondents’ reported use of sunscreen, clothing, sunglasses, and a hat for the Saturday or Sunday the respondent was outdoors. The cover assigned to each body part is based on the body proportions used for assessing extent of burns in medical treatment [34], with further details for head cover as used previously for body cover and exposure indices [28,29,35,36]. The second measure considered whether respondents were using comprehensive measures for reducing UV exposure during peak UV hours on the weekend prior to interview. The dichotomous variable ‘maximal sun protection’ was defined as either (i) having spent less than 15 minutes outdoors on both days of the weekend or (ii) if outdoors on either day during the previous weekend, having protected each region of the body using a hat, covering clothing (three-quarter or long-sleeved tops and three-quarter or long leg cover), and/or sunscreen. The third measure considered the use of at least 1 sun protection behaviour excluding clothing, which is subject to fashion and thermal comfort influences. This variable defined respondents as having used at least 1 sun protection behaviour if they had worn a hat or used sunscreen or stayed mostly under shade during their activity outdoors on the most recent day outdoors on the weekend.

The independent variable of interest was a ‘decade’ variable. Data from 10 discrete summers over 3 decades were categorised into decades as follows: pre-SunSmart: 1987–1988; 1990s: 1991–1992, 1994–1995, 1997–1998, 1999–2000; 2000s: 2000–2001, 2001–2002, 2006–2007; and 2010s: 2010–2011 and 2016–2017. The single summer used to represent the pre-SunSmart period was the only survey conducted before the launch of the SunSmart program. Surveys in 1988–1989 and 1989–1990 were excluded given that these summers were immediately after the establishment of the SunSmart program. Surveys from 2003–2004 and 2013–2014 had only 8 weeks of data collection and were also excluded [29]. No data were collected in the intervening years. Thus, the cross-sectional surveys within each decade provide data representative of the decade.

### Statistical analysis

A total of *N =* 13,285 interviews by respondents aged 14 to 69 years were analysed. These data were weighted to be proportional to the estimated resident population by age and sex and rescaled to retain the original sample size [37]. Cases with missing data (2.5%) were excluded from analyses.

Multivariable regression models were used to examine the change in prevalence across the decades of respondents’ tanning attitudes, individual and combined sun protection behaviours, UV exposure, and sunburn on summer weekends. These dependent variables were described earlier. Additionally, use of sunscreen, a hat, and shade were selected as the focus for the analysis of individual sun protection behaviours, given that the type of clothing worn is commonly subject to fashion influences rather than being worn specifically for sun protection. Two models of maximal sun protection behaviour were analysed, one model with and one without consideration of whether the respondents stayed under shade during their activities outdoors. All models included the following covariates: age group, sex, skin sensitivity, and month of interview. The behavioural models included the relevant weather conditions, and the sunburn model also included the mean UV Index. These covariates have significant theoretical and observed influences on the outcome variables: (i) UV has a causal effect on sunburn prevalence [2,38,39], (ii) past and present ambient temperature influence thermal comfort [40,41], and (iii) month of interview provides a measure of temporal change across the summer, which may reflect temporal changes in respondents’ outdoor activities and desire for a suntan. Note that the model predicting the proportion of respondents outdoors during peak UV hours was modelled for Sunday, given it was only possible to adjust for weather conditions on 1 weekend day.

IBM SPSS Statistics version 24 was used for data merging and coding of variables for analysis. Forced entry logistic regression (dichotomous outcomes) and multiple linear regression (continuous outcomes) were performed using Stata/SE version 12.1. These models generally conformed with statistical assumptions [42,43], including non-collinearity of variables and valid standard errors of parameter estimates. However, inadequate model fit (Hosmer–Lemeshow test, *p* < 0.05) was observed for the 3 outcome variables outdoors during peak UV, hat use, and shade use; therefore, caution is needed when interpreting the predictive power of these models [44]. Margins analyses were performed using Stata/SE version 12.1 to predict the adjusted prevalence figures and 95% confidence intervals (CI) for each outcome and decade. Further details of prevalence for individual sun protection behaviours, including mean change in body cover index across the survey decades, are presented in the S2 and S3 Tables. The research questions and outcome variables, with the exception of the variable ‘use of at least 1 sun protection behaviour’, were defined prior to analyses commencing. Following preliminary analyses, it was decided that adding analysis of ‘use of at least 1 sun protection behaviour’ would provide a more complete picture of the levels of sun protection adopted by the population.

## Results

### Respondents surveyed

Table 1 describes the demographic characteristics of respondents pooled across each decade. The distributions by age and sex reflect the distribution of the population of Melbourne residents aged 14 to 69 years in each survey year. Most survey respondents had skin that was highly or moderately sensitive to sunburn. Although the proportion of respondents with less sensitive skin increased slightly in the 2010s, recruitment by skin type was relatively equal across the decades. The increase in respondents attaining higher education across the decades was consistent with changes in the population [45,46].

**Table 1:** Demographic characteristics of respondents by decade.

Characteristic	Pre-SunSmart *N =* 1,655	1990s *N =* 5,258	2000s *N =* 3,385	2010s *N =* 2,987
**Unweighted percent, n (%)**				
Sex				
Male	799 (48.3)	2,605 (49.5)	1,672 (49.4)	1,484 (49.7)
Female	856 (51.7)	2,653 (50.5)	1,713 (50.6)	1,503 (50.3)
Age, years				
14–24	494 (29.9)	1,549 (29.5)	933 (27.6)	1,239 (41.5)^a^
25–44	797 (48.2)	2,601 (49.5)	1,490 (44.0)	843 (28.2)
45–69	364 (22.0)	1,108 (21.1)	962 (28.4)	905 (30.3)
Skin sensitivity^b^				
Highly sensitive	475 (29.5)	1,650 (32.2)	935 (28.3)	749 (25.8)
Moderately sensitive	751 (46.6)	2,346 (45.8)	1,573 (47.6)	1,333 (45.8)
Not sensitive	385 (23.9)	1,129 (22.0)	796 (24.1)	826 (28.4)
**Weighted percent**^c^				
Highest education level^d^				
School qualification	985 (70.8)	2,593 (58.7)	1,416 (49.3)	1,022 (40.0)
Post-school qualification	407 (29.3)	1,822 (41.3)	1,455 (50.7)	1,532 (60.0)

^a^The sample for respondents aged 14–24 years was boosted in 2010–2011 (*N =* 967).

^b^Excludes individuals (1987–1988: *N =* 44; 1990s: *N =* 133; 2000s: *N =* 81; 2010s: *N =* 79) who could not specify their skin type.

^c^Weighted percentages are reported for educational attainment.

^d^Includes only respondents aged 20 to 64 years, to enable comparison with the educational attainment among the population [45,46]. Comparison between respondents with a highest education level attained of school qualification (matriculation/high school certificate/senior certificate) compared with post-school qualification (university or other tertiary institution certificate, diploma, or degree).

### Tanning attitudes

Preventive beliefs and attitudes regarding suntans increased among respondents in all decades relative to the baseline (Table 2). In 1987–1988 a minority of respondents reported they did not like to get a suntan (adjusted prevalence [AP] = 43.1%). The prevalence of this aversion to suntanning increased rapidly in the 1990s and peaked in the 2010s with the majority of respondents reporting they did not like to get a suntan (AP = 66.1%; adjusted odds ratio [AOR] = 2.78, 95% CI 2.41 to 3.20, *p <* 0.001). In contrast, many respondents in 1987–1988 disagreed with the statement ‘A suntanned person is more healthy’ (AP = 82.3%). The prevalence of this health belief increased among respondents in the 1990s (AP = 90.3%; AOR = 2.02, 95% CI 1.70 to 2.41, *p <* 0.001), but was slightly lower in subsequent decades, albeit remaining above the baseline decade. Social norms also became less supportive of tanning in the 1990s. The prevalence of respondents who disagreed with the statement ‘Most of my friends think a suntan is a good thing’ increased from AP = 35.9% in the 1980s to AP = 63.1% in the 1990s (AOR = 3.43, 95% CI 2.99 to 3.92, *p <* 0.001). This improvement appeared to weaken in the subsequent decades (2000s: AOR = 2.57, 95% CI 2.24 to 2.96, *p <* 0.001; 2010s: AOR = 2.52, 95% CI 2.17 to 2.91, *p <* 0.001).

**Table 2:** Changes in tanning attitudes among Melbourne residents by decade, relative to before the SunSmart program.^a^

Tanning attitudes^b^	Pre-SunSmart *N* = 1,655		1990s *N* = 5,258		2000s *N* = 3,385		2010s *N* = 2,987	
	AP	AOR (95% CI)	AP	AOR (95% CI)	AP	AOR (95% CI)	AP	AOR (95% CI)
Do you like to get a suntan? (no)	43.1%	1.00 (Ref)	65.0%	**2.63 (2.31, 3.00)**	59.7%	**2.06 (1.80, 2.36)**	66.1%	**2.78 (2.41, 3.20)**
A suntanned person is more healthy (disagree)^b^	82.3%	1.00 (Ref)	90.3%	**2.02 (1.70, 2.41)**	88.3%	**1.63 (1.36, 1.96)**	89.8%	**1.92 (1.58, 2.32)**
Most of my friends think a suntan is a good thing (disagree)^b^	35.9%	1.00 (Ref)	63.1%	**3.43 (2.99, 3.92)**	57.0%	**2.57 (2.24, 2.96)**	56.5%	**2.52 (2.17, 2.91)**

Bold face indicates statistical significance at p<0.05 level.

^a^Models were adjusted for the covariates age, sex, skin sensitivity, and month of interview.

^b^Responses were categorised into protective beliefs, i.e., strongly disagree or mildly disagree with the statement, or unprotective beliefs, i.e., strongly agree, mildly agree, neither agree nor disagree, or can’t say with the statement.

AOR, adjusted odds ratio; AP, adjusted prevalence; CI, confidence interval.

### UV exposure and sun protection behaviour

**Fig 1:**
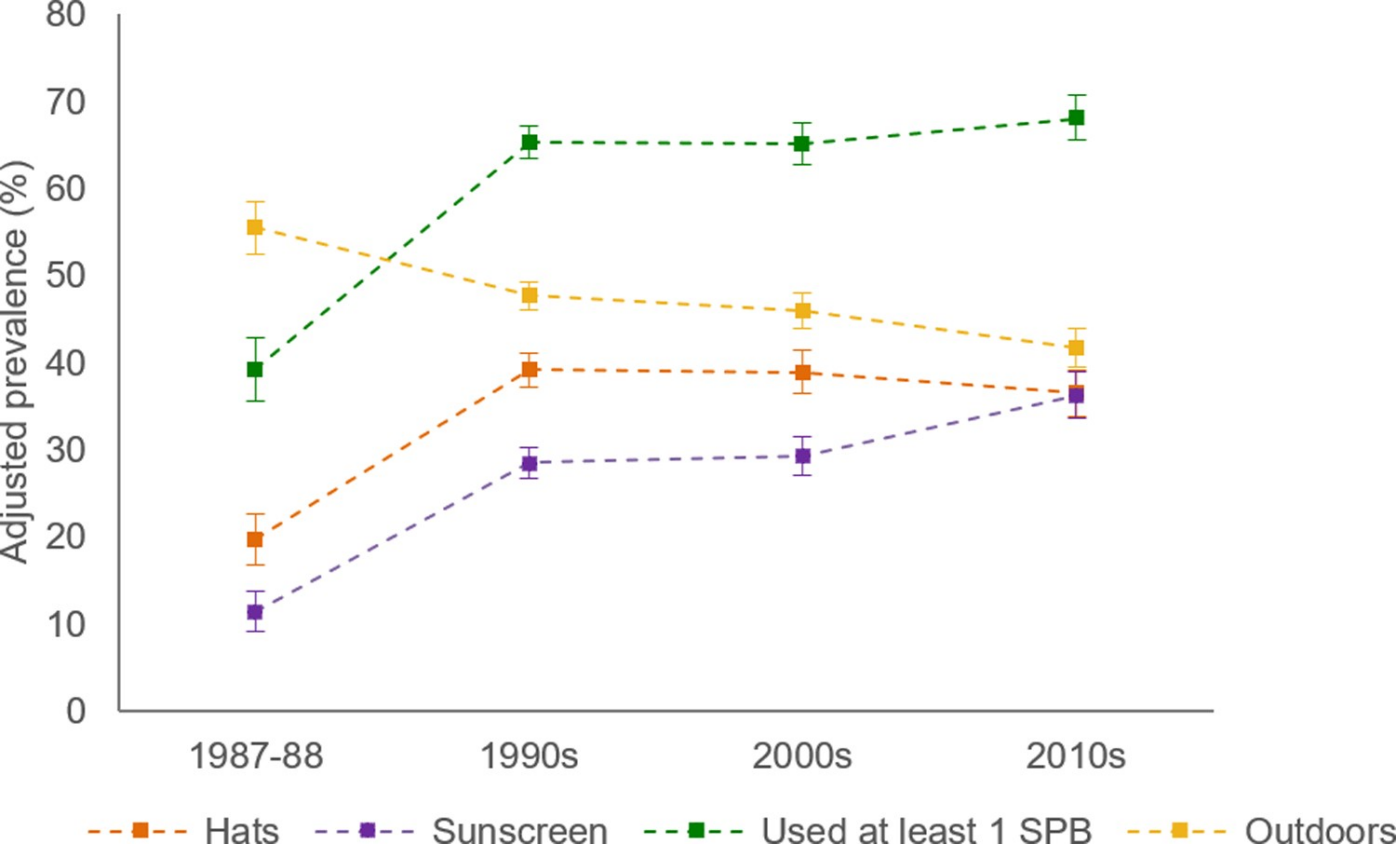
Adjusted prevalence of sun protection behaviours among Australian adults aged 18–69 years (1987–1988 to 2010s). Used at least 1 sun protection behaviour (SPB) defined as specifically used sunscreen or wore a hat or stayed mostly in the shade when outdoors during peak ultraviolet radiation hours on summer weekends. Data points represent cross-sectional samples from summers in each decade.

**Fig 2:**
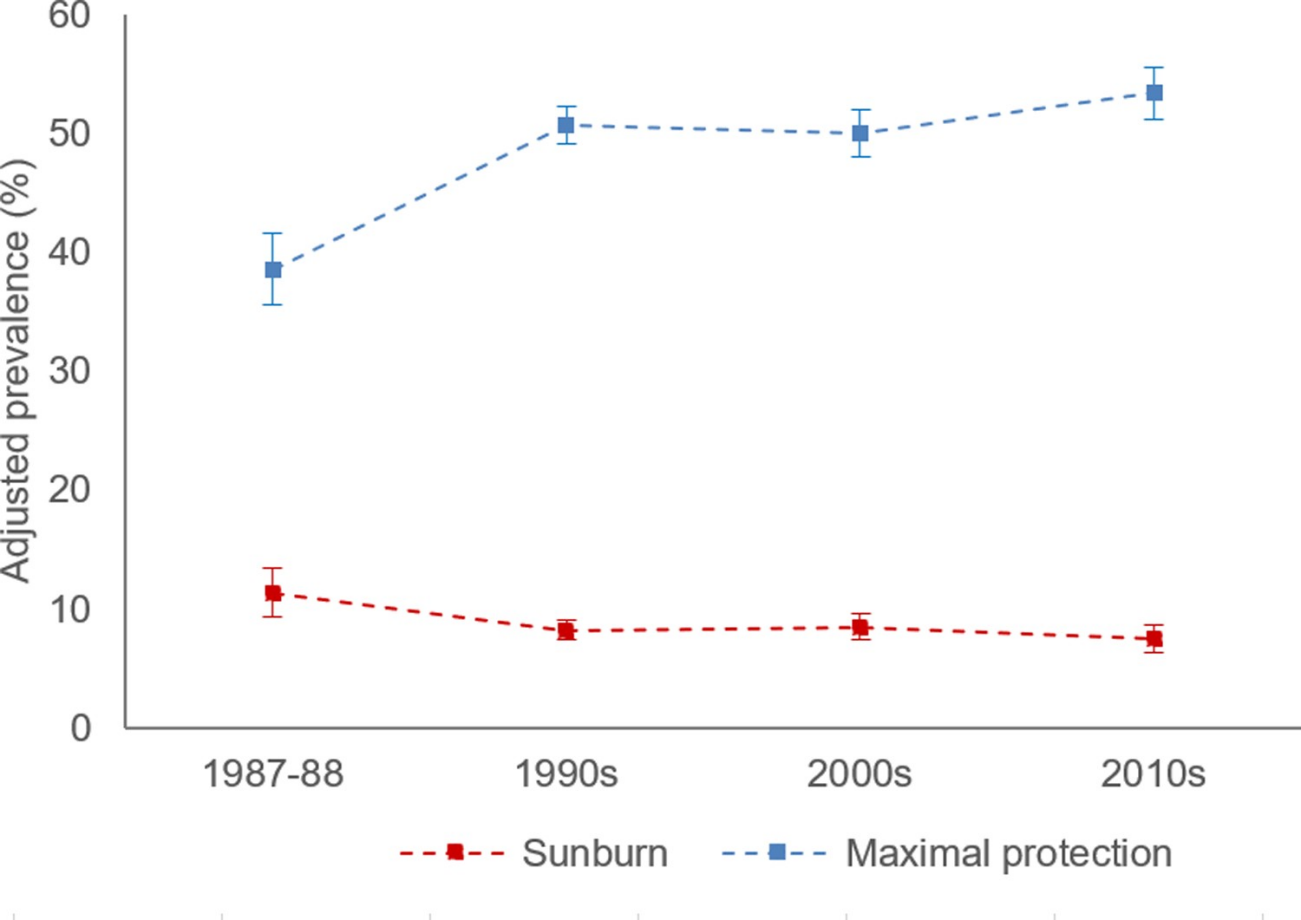
Adjusted prevalence of maximal sun protection and sunburn among Australian adults aged 18–69 years (1987–1988 to 2010s). Data points represent cross-sectional samples from summers in each decade.

Table 3 and Figs 1 and 2 describe the changes in UV exposure, individual and composite sun protection behaviours, and sunburn experience of respondents on summer weekends across the decades. Further details on change in the amount of time spent outdoors, the use of covering clothing, and mean body cover across 3 decades are available in S2 and S3 Tables.

**Table 3:** Sun exposure and sun protection use among Melbourne residents by decade, relative to before the SunSmart program.

Outcome variable	Pre-SunSmart		1990s		2000s		2010s	
	AP	AOR (95% CI)	AP	AOR (95% CI)	AP	AOR (95% CI)	AP	AOR (95% CI)
**Proportion outdoors on Sunday**	*N* = 1,501		*N* = 4,752		*N* = 2,940		*N* = 2,623	
Outdoors on Sunday (yes)	55.5%	1.00 (Ref)	47.7%	**0.72 (0.62, 0.84)**	46.0%	**0.67 (0.58, 0.79)**	41.8%	**0.57 (0.48, 0.66)**
**Specific sun protection used outdoors**	*N* = 1,064		*N* = 2,984		*N* = 1,802		*N* = 1,587	
A hat (yes)	19.7%	1.00 (Ref)	39.2%	**2.79 (2.24, 3.49)**	38.9%	**2.75 (2.19, 3.45)**	36.5%	**2.46 (1.95, 3.10)**
Sunscreen (yes)	11.4%	1.00 (Ref)	28.5%	**3.25 (2.52, 4.19)**	29.3%	**3.38 (2.60, 4.39)**	36.3%	**4.73 (3.65, 6.13)**
Stayed mostly in the shade (yes)	19.3%	1.00 (Ref)	25.7%	**1.47 (1.17, 1.83)**	25.3%	**1.43 (1.14, 1.80)**	28.2%	**1.68 (1.33, 2.11)**
**Composite behaviour**	*N* = 1,064		*N* = 2,984		*N* = 1,802		*N* = 1,587	
At least 1 SPB^a^ (yes)	39.3%	1.00 (Ref)	65.3%	**3.04 (2.52, 3.68**)	65.1%	**3.01 (2.47, 3.67)**	68.1%	**3.46 (2.83, 4.23)**
**Maximal sun protection**	*N* = 1,457		*N* = 4,612		*N* = 2,785		*N* = 2,496	
Both days^b^ excluding shade (yes)	27.9%	1.00 (Ref)	37.4%	**1.57 (1.33, 1.85)**	36.8%	**1.52 (1.28, 1.81)**	39.1%	**1.69 (1.42, 2.00)**
Both days^b^ including shade (yes)	38.7%	1.00 (Ref)	50.7%	**1.68 (1.44, 1.97)**	50.0%	**1.63 (1.39, 1.92)**	53.4%	**1.89 (1.60, 2.22)**
**Weekend sunburn**	*N* = 1,407		*N* = 4,610		*N* = 2,768		*N* = 2,499	
Burnt either day (yes)	11.4%	1.00 (Ref)	8.2%	**0.68 (0.53, 0.88)**	8.5%	**0.71 (0.54, 0.94)**	7.6%	**0.62 (0.47, 0.82)**

Bold face indicates statistical significance at *p* < 0.05 level. Models were adjusted for the covariates age, sex, skin sensitivity, survey month, weekend temperature, median monthly temperature, number of days with heavy cloud cover (≥6 oktas) in past month, and number of days with rainfall (≥1 mm) in past month. Additionally, the sunburn model was adjusted for the mean UV Index during peak UV hours (i.e., 11 AM to 3 PM DST) on the weekend. The models included all respondents who were outdoors in the Melbourne metropolitan area during peak UV hours on the weekend. Additionally, the outdoors, maximal sun protection, and sunburn models included respondents indoors during peak UV hours. The sunburn model excludes respondents who were burnt at times other than during peak UV hours on the weekend.

^a^At least 1 SPB defined as using a hat and/or using sunscreen and/or staying mostly under shade during their outdoor activity.

^b^Maximal sun protection defined as staying indoors on both days of the weekend or, if outdoors, wearing a hat, covering clothing, and/or sunscreen on each day of the weekend that the respondent was outdoors.

AOR, adjusted odds ratio; AP, adjusted prevalence; CI, confidence interval; SPB, sun protection behavior; UV, ultraviolet radiation.

Long-term reductions in UV exposure among Melbourne residents were observed. Overall, the majority of respondents spent 15 minutes or more outdoors during peak UV hours on at least 1 day on the weekend (unadjusted prevalence of respondents outdoors on either day: 1987–1988, 75.8%; 1990s, 68.3%; 2000s, 70.1%; 2010s, 68.9%). However, the prevalence of respondents outdoors on the Sunday prior to interview was lower (AP 1987–1988 to 2010s: 55.5% to 41.8%). This latter measure of the odds of respondents being outdoors during peak UV hours was adjusted for weather conditions, and demonstrated a significant decline across the decades relative to 1987–1988 (1990s: AOR = 0.72, 95% CI 0.62 to 0.84, *p <* 0.001; 2000s: AOR = 0.67, 95% CI 0.58 to 0.79, *p <* 0.001; 2010s: AOR = 0.57, 95% CI 0.48 to 0.66, *p <* 0.001). The mean length of time respondents spent outdoors on weekends also decreased (1987–1988: 120 min, SE 2.7; 1990s: 118 min, SE 1.5; 2000s: 111, SE 1.9; 2010s: 101 min, SE 2.0).

In the summer before the SunSmart program commenced, relatively few respondents had used SPF 12+ sunscreen (AP = 11.4%) or worn a hat (AP = 19.7%) on summer weekends. Fig 1 illustrates that the use of these specific behaviours greatly increased in the first decade after the initiation of the SunSmart program. In the subsequent decades there was little change in the prevalence of hat wearing. However, another increase in sunscreen use was observed in the 2010s, reaching a nearly 5-fold increase in the odds of sunscreen use relative to 1987–1988 (AOR = 4.73, 95% CI 3.65 to 6.13, *p <* 0.001). The highest adjusted prevalence of sunscreen use attained across the decades was 36.3% of respondents outdoors on summer weekends in the 2010s. Shade use was also relatively uncommon in 1987–1988 (AP = 19.3%). Although it increased across the decades, the greatest use observed was in the most recent decade, when less than one-third of respondents stayed mostly in the shade outdoors (AP = 28.2%; AOR = 1.68, 95% CI 1.33 to 2.11, *p <* 0.001).

A much higher prevalence of sun protection behaviour was evident when assessing respondents’ composite behaviour. Moreover, respondents’ use of clothing, hats, and sunscreen on summer weekends protected on average 72% of the body from UV before SunSmart, which increased to 79% in the 1990s and 2000s and 80% in the 2010s. The adjusted prevalence of use of at least 1 sun protection behaviour increased from 39.3% in 1987–1988 to 65.3% in the 1990s and 68.1% by the 2010s among respondents outdoors in peak UV hours (AOR = 3.46, 95% CI 2.83 to 4.23, *p <* 0.001). Use of maximal sun protection behaviour on both weekend days, including staying indoors during peak UV hours, also increased, from 27.9% in 1987–1988 to 39.1% in the 2010s (AOR = 1.69, 95% CI 1.42 to 2.00, *p* < 0.001). Additionally, when considering respondents who stayed in the shade during their activities outdoors in peak UV hours on the weekend, a nearly 2-fold shift in maximal sun protection behaviour was observed in the decades following the launch of SunSmart, with approximately half of respondents well protected from UV on summer weekends during this period (AP: 1987–1988, 38.7%; 1990s, 50.7%; 2000s, 50.0%; 2010s, 53.4%).

### Sunburn

The odds of sunburn were lower in all decades relative to the baseline before SunSmart and lowest in the 2010s (AOR = 0.62, 95% CI 0.47–0.82, *p <* 0.01). Moreover, Fig 2 illustrates that there is good concordance between the pattern of increase in the prevalence of use of maximal sun protection with shade and the decrease in sunburn prevalence across the decades.

## Discussion

This study provides evidence of substantial improvements in Melbourne residents’ skin cancer prevention attitudes and behaviour and their experience of sunburn in the decades since SunSmart was implemented. The reported use of sun protection rose rapidly in the 1990s from a low base, suggesting that in the initial years of the program it was relatively easy to shift the early adopters to comply with sun protection messages. In later decades improvement in preventive attitudes and behaviours was more gradual. Compellingly, a reduction in sunburn prevalence corresponding with the increased use of sun protection was also evident.

Relative to past studies for this population, we present a more complete appraisal of the spectrum of sun protection behaviours adopted by the population exposed to SunSmart messages. The approach considering composite behaviours revealed a higher level of sun protection behaviour than previously published for this survey, but this level was consistent with the prevalence of sunscreen use and weekend sunburn when analysing change for individual years from 1987 to 2007 [29]. Our analysis highlights the importance of recognising that reducing exposure to UV involves numerous behaviours that individually or together may provide adequate protection from UV. Additionally, a significant reduction in prevalence and amount of time spent outdoors during peak UV hours across the decades was evident, with the greatest reduction in the most recent decades, which had not previously been observed [29]. This suggests that pooling the survey years into decades in this study was beneficial for detecting the cumulative effects of incremental changes from year to year. The finding that a considerable proportion of respondents are staying indoors during peak UV hours on both weekend days or staying under shade when outside suggests that these behaviours have an important and increasing role in providing adequate levels of UV protection on summer weekends.

Of specific interest is whether or not the findings provide evidence of a sufficient shift in the population’s sun protection behaviour to have contributed to the downturn in melanoma incidence rates among younger cohorts in Australia. Indeed, the timing (with greatest improvement in the 1990s) and the size of the behaviour change (2-fold to 4-fold) observed for the Melbourne population suggests this is feasible. The cross-sectional study design and absence of a control population are limiting for this study. Nonetheless, implementation of a randomised trial to provide evidence of a causal link between SunSmart and the decline in skin cancer rates would be impractical in the context of the population-wide skin cancer prevention programs implemented across Australia [47]. Multiple studies have aimed to bridge this gap in evidence between prevention programs and behavioural outcomes, including by measuring program reach [12], monitoring the growing prevalence of sun protection policies and regulations [12], and analysing the association between the dose of campaign advertising and sun protection behaviour [28,31]. Moreover, the programs implemented are underpinned by a strong theoretical foundation including epidemiological evidence that UV causes skin cancer [1,39,48], evidence of the effectiveness of sun protection measures [49–53], and health promotion and behaviour change principles [12,54].

Although it is possible that secular trends such as changing interest in outdoor activities and increased immigration from populations with low melanoma risk may explain some of the decline in melanoma rates in Australia [17], the large program investment [12] and shift in sun protection behaviours observed in the current study precede the substantial increases in screen time due to the evolution of smart phones and social media in the early 2000s. Furthermore, it is unlikely the more protective sunscreen formulations introduced in the early 2000s would have yet had time to produce a substantial impact on melanoma rates, given an estimated 20-year lag between exposure and development of melanoma [55]. The strong association between the extent of exposure to SunSmart campaign advertising and weekend sun protection behaviour [28,31] suggests that program activities are a significant driver of behaviour change. Moreover, program-driven environmental changes [12], including sun protection policies [56,57] and provision of shade [12], have been shown to be effective in prompting and supporting increased sun protection behaviour.

It appears more likely than not that the operation of the SunSmart skin cancer prevention program over more than 3 decades has reduced the population’s exposure to UV, which may have contributed to a reduced burden of melanoma. It is estimated that every dollar invested in skin cancer prevention programs returns AU$3.20 in reduced burden of the disease [58]. It is therefore important to continue to regularly motivate the population and provide supportive environments to ensure that an adequate level of sun protection is sustained among the population. It is reasonable to expect that, without commitment to environmental change and public-awareness-raising activities by skin cancer prevention programs, a slow decline in sun protection of future cohorts may ensue [59]. The consistency of the survey methods used across the years is a strength of the study given that this minimised bias in analysing changes in prevalence over time. The time- and location-based self-report measures have shown good congruence with observed sun protection behaviour in public outdoor settings over time when assessing long-term behaviour change [35]. Accordingly, any social desirability bias that might otherwise explain change over time in reported behaviour is likely to be relatively small [35]. Congruence with the patterns of change over time nationally also provides further validation of the findings. Specifically, the increase in preventive attitudes regarding tanning and continued increase in use of sunscreen, reduction in time spent outdoors in peak UV hours, and reduced sunburn among Melbourne residents in the 2010s appeared relatively consistent with improvements observed nationally for the period from 2003 to 2011 [60].

There is limited comparability of these time- and location-based measures of recent behaviour with those used by population monitoring studies internationally [61,62]. The composite sun protection measure used in the longest running national sun protection survey in the US is based on the use of sunscreen or covering clothing or seeking shade on a regular basis, namely ‘most of the time’ or ‘always’, when outside for 1 hour or more in summer [63,64]. This composite measure used in the US is similar to the measure presented in the current study for minimal protection, i.e., use of least 1 sun protection behaviour, although the latter is based on sun exposure for a recent day during peak UV hours rather than usual behaviour. The prevalence of use of sunscreen and use of at least 1 sun protection behaviour among US and Australian adults appear to be of similar magnitude, with a somewhat similar pattern of increase during the past 2 decades, albeit with a stronger increase in the 2010s decade in Australia. However, there is no corresponding decrease in sunburn for this period in the US. It is probable that the measure of usual behaviour recalled over a longer time frame and for an extended period of sun exposure (>1 hour) overestimates UV protection. In high-UV environments during summer, a fair-skinned person will experience sunburn in less than 1 hour [65]. In the current study, the pattern of change in the prevalence of maximal sun protection corresponded most closely with the change in sunburn over time, compared with the other sun protection measures, highlighting the effectiveness of using either sun avoidance or multiple sun protection strategies together. This is encouraging given that the maximal sun protection measure is in line with the WHO guidelines for sun protection [9,66], which recommend limiting time in the midday sun and using a combination of sun protection behaviours when outdoors. However, recall of sunburn over an extended period of time (i.e., over the past month or past summer), rather than 2 to 3 days ago, may also explain the limited change in sunburn prevalence observed in the US. Nevertheless, annual surveys of sunburn rates among adults in Denmark, using a measure comparable to that in the US, did detect a small decline in sunburn following an intensive public education campaign [67], so other factors may be involved. It is interesting that the changes to sun protection in the US have occurred with minimal public education on skin cancer prevention. Chang et al. highlight the other socio-cultural influences on sun protection historically, including changes in swimwear fashions, travel, and leisure time in the past century [68].

One limitation of the study is that the measures were focused on behaviour during peak UV hours on summer weekends, although respondents may have had considerable UV exposure at other times. Additionally, in defining maximal sun protection behaviour, we acknowledge that small amounts of UV exposure may occur even when staying indoors or applying sunscreen to all exposed skin when outdoors [22,69]. The self-reported nature of the survey is a further limitation of the measures, but the survey is generally considered reliable [35,70]. We also note a large shift in educational attainment among respondents across the decades. Although this is consistent with the significant increase in school retention and tertiary education in Victoria [71], this and increased exposure to many health campaigns more broadly across the decades may have contributed to improved sun-related attitudes and behaviours [72]. Finally, the findings are limited to the years the survey was conducted, and unmeasured variation within the decades due to non-surveyed years is probable; however, this is unlikely to be substantial given the overall patterns of change observed across the decades. Nonetheless, following 30 years of SunSmart, despite significant behaviour change, the prevalence of use of adequate sun protection behaviours is still less than ideal. Sustained high-reach multi-media campaigns and community programs along with innovative strategies are needed to continue to improve the population’s sun protection behaviour in Australia. Novel strategies adopted recently for skin cancer prevention in Victoria include promoting daily sunscreen use [73] and creating shade in community settings. The state government is continuing to support creation of shade in community settings with a further AU$15.1 million in shade grants planned for 2019 to 2023 [27]. For countries yet to achieve significant behaviour change, we recommend new programs begin with identifying skin cancer as a problem to be addressed by the community and continually reminding the community of this need. Strategies implemented must be of sufficient scale, with well-funded campaigns and programs to ensure widespread and sustained population reach, and with advocacy for policy and legislative changes to create supportive environments for UV protection across varied community settings.

## Conclusions

This comprehensive analysis of changes in reported sun protection behaviour among Melbourne residents over a period of 3 decades shows a significant and sustained improvement. The timing and size of the shift in preventive behaviours implies that SunSmart is likely to have contributed to the reduced incidence in melanoma among younger cohorts observed in recent years. Although definitive evidence of the impact of the SunSmart program on skin cancer rates remains elusive, prevention programs should be supported to maintain and further their strategies, given that lifelong protection is beneficial in reducing risk of skin cancer [1].

## Supporting information

S1 Appendix Sun protection survey questionnaire (2000–2001). (DOC)

S2 Appendix Sun protection survey questionnaire (2016–2017). (DOCX)

S1 Table Sun protection survey questions and response categories. (DOCX)

S2 Table Tanning attitudes and sun protection behaviour among respondents outdoors during peak UV hours (1987–1988 to 2010s). (DOCX)

S3 Table Mean change in the proportion of the body protected by sunscreen, clothing, or a hat when outdoors per decade, relative to before the SunSmart program. (DOCX)

S1 STROBE checklist Checklist of items for cross-sectional studies. (DOC)

## Abbreviations

AOR: adjusted odds ratio
AP: adjusted prevalence
UV: ultraviolet radiation
