# Supplementary material for: Implementation of the SunSmart program and population sun protection behaviour in Melbourne, Australia: Results from cross-sectional summer surveys from 1987 to 2017
Source: PLoS Med. 2019 Oct 8;16(10):e1002932. doi: 10.1371/journal.pmed.1002932 (PMC6782093; doi:10.1371/journal.pmed.1002932)
Supplement: S1 Appendix — (DOC) [file pmed.1002932.s001.doc]

**S1 Appendix.** Sun Protection Survey questionnaire (2000-01)

29-NOV-00

ROY MORGAN RESEARCH STRICTLY CONFIDENTIAL

2nd Floor Rear, 411 Collins Street, Melbourne, Vic., 3000 CM-2418

Tel: (03) 9629-6888 Dec/Jan/Feb 2000/2001

SUN PROTECTION SURVEY 2000/2001

________________________________________________________________________________

QP. Good %A. My name is <SAY NAME>. | 25-29............ 4

I'm from Roy Morgan Research , the |

people who conduct the Morgan Gallup | 30-34............ 5

Poll. Today we're doing a survey about |

people's attitudes towards being out | 35-39............ 6

in the sun, and we'd like the opinion |

of people aged 14 to 69. May I speak | 40-44............ 7

to the youngest male at home aged 14 |

years or over? | 45-49............ 8

IF NO MALES ASK: Then may I speak to |

the youngest female at home aged 14 | 50-54............ 9

years or over? |

| 55-59............ 10

IF NO-ONE AT HOME AGED 14-69, RECORD |

INELIGIBLE HOUSEHOLD BELOW | 60-64............ 11

|

ELIGIBLE 14-69 | 65-69............ 12

YEARS............ 1 |

| QUOTA CHECK

INELIGIBLE |

HOUSEHOLD........ 2 | MALE UNDER 35

| YEARS............ 1

IF INELIGIBLE HOUSEHOLD |

| MALE 35 OR OVER.. 2

+------------------------------------+ |

| Thank you for your time and | | FEMALE UNDER 35

| assistance | | YEARS............ 3

+------------------------------------+ |

| FEMALE 35 OR OVER 4

QA. RECORD SEX OF RESPONDENT (DO NOT |

ASK!) | +------------------------------------+

| | I'm sorry %569,/sir/madam/ but we |

MALE............. 1 | | have already interviewed enough |

| | %571,/males/females/ of your age |

FEMALE........... 2 | | group for our survey. |

| +------------------------------------+

AGE1. To make sure we have a true |

cross-section of people, would you | QB. May I have your postcode please?

mind telling me your approximate age |

please? | IF RESPONDENT DOESN'T KNOW, ASK: Well,

| could you please tell me the suburb in

IF REFUSES READ OUT. | which you live?

|

14-17............ 1 | ESC 0 AND TYPE IN SUBURB, LOOK UP AND

| FILL IN POSTCODE BEFORE COMPLETION OF

18-19............ 2 | INTERVIEW.

|

20-24............ 3 | |__|__|__|__+

|

25-29............ 4 | Q1. Now, I'd like to ask a few

| questions about your skin type.

30-34............ 5 |

| Suppose your skin was exposed to

35-39............ 6 | strong sunshine at the beginning of

| summer with no protection at all. If

40-44............ 7 | you stayed in the sun for 30 minutes,

| would your skin: (READ OUT)

45-49............ 8 | IF SAYS 'GO RED' INTERPRET AS A BURN

| AND ASK:

50-54............ 9 | Would you then tan afterwards or not?

|

55-59............ 10 | Just burn and not

| tan afterwards... 1

60-64............ 11 |

| Burn first, then

65-69............ 12 | tan afterwards,

| or............... 2

REFUSED.......... 13 |

| Not burn at all,

IF RESPONDENT REFUSED TO GIVE AGE: | just tan......... 3

AGE2. ESTIMATE AGE OF RESPONDENT |

| (DO NOT READ)

14-17............ 1 | NOTHING WOULD

| HAPPEN........... 4

18-19............ 2 |

| (DO NOT READ)

20-24............ 3 | CAN'T SAY........ 5

SUN PROTECTION SURVEY 2000/2001

(C) Roy Morgan Research Pty Ltd. 2000.

DATE 29-NOV-00 SUN PROTECTION SURVEY 2000/2001 PAGE 2

________________________________________________________________________________

Q2. How would you describe your skin | IF SUNBURNT (CODES 1 OR 2 ON Q4A), ASK

colour when you don't have any tan? |

| Q4B. Which part or parts of you got

IF RESPONDENT SAYS 'FAIR' OR 'DARK' | sunburnt at the weekend? Where else?

ASK: Would that be very fair/dark or |

fair/dark? | FACE............. 1,

|

IF RESPONDENT HESITATES, PROMPT | NOSE............. 2,

WITH:When you don't have any tan would |

you say your skin colour is... | HEAD............. 3,

(READ OUT) |

| EARS............. 4,

Very fair........ 1 |

| CHEST............ 5,

|

Fair............. 2 | STOMACH.......... 6,

|

| BACK............. 7,

Medium........... 3 |

| NECK............. 8,

Olive............ 4 |

| SHOULDERS........ 9,

Dark............. 5 |

| ARMS............. 10,

Very Dark........ 6 |

| HANDS............ 11,

Black............ 7 |

| LEGS............. 12,

(DO NOT READ) |

DON'T KNOW/ CAN'T | BACK OF KNEES.... 13,

SAY.............. 8 |

| FEET............. 14,

ASK EVERYONE: |

Q3A. Have you made any attempt to get | IF BURNT IN MORE THAN ONE PLACE, ASK:

a suntan this season? | Q4C. Which part was burnt WORST?

|

YES.............. 1 | FACE............. 1

|

NO............... 2 | NOSE............. 2

|

Q3B. Do you like to get a suntan or | HEAD............. 3

not? |

| EARS............. 4

YES.............. 1 |

| CHEST............ 5

NO............... 2 |

| STOMACH.......... 6

IF LIKES TO GET A SUNTAN (CODE 1 ON |

Q3B) | BACK............. 7

|

Q4. How DEEP a tan do you like to get? | NECK............. 8

|

| SHOULDERS........ 9

IF RESPONDENT SAYS "GOLDEN", SAY: Does |

that mean light or moderate? | ARMS............. 10

|

IF RESPONDENT HESITATES PROMPT WITH: | HANDS............ 11

Do you like that tan to be.. |

(READ OUT) | LEGS............. 12

|

Light............ 1 | BACK OF KNEES.... 13

|

Moderate......... 2 | FEET............. 14

|

Dark............. 3 | Q5AI.Which of the following statements

| best describes the burn on your %130.?

Very Dark........ 4 |

| (READ OUT)

(DO NOT READ) |

CAN'T SAY........ 5 | Red without being

| tender........... 1

+------------------------------------+ |

| The next questions are about | | Red and tender... 2

| sunburn. By sunburn we mean any | |

| amount of reddening of the skin | | Red, tender and

| after being in the sun. | | blistered........ 3

+------------------------------------+ |

| IF BURN WAS RED WITHOUT BEING TENDER

Q4A. Did you get at all sunburnt | (CODE 1 ON Q5AI), ASK:

yesterday? What about on Saturday? |

| Q5AII.Was the redness present the next

SUNDAY........... 1, | morning?

|

SATURDAY......... 2, | YES.............. 1

|

NEITHER DAY...... 3, | NO............... 2

SUN PROTECTION SURVEY 2000/2001

(C) Roy Morgan Research Pty Ltd. 2000.

DATE 29-NOV-00 SUN PROTECTION SURVEY 2000/2001 PAGE 3

________________________________________________________________________________

COULDN'T DECIDE.. 3 | +------------------------------------+

| | Next we'd like to ask about your |

| | outdoor activities over the |

| | weekend between 11am and 3pm |

DON'T KNOW | | because that's when the sun shines |

(DIDN'T LOOK).... 4 | | strongest. |

| +------------------------------------+

|

| Q6B. Thinking back to Sunday. Were you

IF BLISTERED (CODE 3 ON Q5AI), ASK: | out of doors for longer than 15

| minutes between 11am and 3pm? By out

| of doors we mean not in a building and

| not in a covered vehicle.

Q5B. Are the blisters weeping? | IF INTERMITTENTLY OUTDOORS ASK: Would

| you say you were actually out of doors

YES.............. 1 | for longer than 15 minutes in total?

|

| YES.............. 1

|

NO............... 2 | NO............... 2

================================================================================

IF YES ON SUNDAY: | OT- | CAN

| HER | 'T

Q7. What activity were | (S- | SAY 998

you DOING MOSTLY during | PE- |

that time out of doors? | CI- |

| FY) 997 |

| |

IF OTHER, HIGHLIGHT | |

OTHER AND TYPE IN | |

RESPONSE | |

================================================================================

Q8. About how much time did you spend | Q9A. Were you in the Melbourne

out of doors on Sunday between 11am | metropolitan area when you were %O

and 3pm %O %135.? | %135.?

TYPE IN THE TOTAL IN MINUTES. |

| YES.............. 1

IF CAN'T SAY ESC D |

| NO............... 2

|__|__|__+ |

================================================================================

Q10A.While you were %O %135., was it | Q11A1.Now we need to ask you some

MOSTLY SUNNY or MOSTLY CLOUDY? | questions in detail about what you

| were wearing yesterday (SUNDAY) to

MOSTLY SUNNY..... 1 | find out how much your skin was

| exposed to direct sunlight.

SOMETHING IN | Would you mind telling me what you

BETWEEN | were wearing while you were %O %135.?

HAZY/LIGHT |

CLOUDY/HALF SUNNY | IF RESPONDENT HESITATES, SAY:

AND HALF CLOUDY.. 2 | Can you remember what you were wearing

| on the top part of your body?

MOSTLY CLOUDY.... 3 |

| RETURN FOR CLOTHING WORN ON LOWER PART

CAN'T SAY........ 4 | OF BODY

|

| TOP/DRESS/WETSUIT 1

|

| SWIMWEAR......... 2

|

| TOPLESS.......... 3

|

| Q11A2. CLOTHING WORN ON LOWER PART OF

| BODY

|

Q10B.Were you MOSTLY IN THE SHADE or | RECORD OR IF RESPONDENT HESITATES

MOSTLY OUT IN THE OPEN while you were | PROMPT WITH:

%O %135.? | Can you remember what you were wearing

| on the lower part of your body?

IN THE SHADE..... 1 |

| TROUSERS/JEANS/S-

IN THE OPEN...... 2 | HORTS/SKIRT/DRESS

| /WETSUIT......... 1

IN SHADE AND OUT |

IN OPEN EQUALLY.. 3 | SWIMWEAR......... 2

|

CAN'T SAY........ 4 | BOTTOMLESS....... 3

SUN PROTECTION SURVEY 2000/2001

(C) Roy Morgan Research Pty Ltd. 2000.

DATE 29-NOV-00 SUN PROTECTION SURVEY 2000/2001 PAGE 4

________________________________________________________________________________

IF TOP/DRESS/WETSUIT (CODE 1 ON | Q11F.What length were the socks?

Q11A1), ASK |

| KNEE............. 1

Q11B.How long were the sleeves of your |

top/dress/wetsuit? | ANKLE............ 2

IF RESPONDENT HESITATES, READ OUT: |

|

WRIST LENGTH..... 1 | ASK OR RECORD:

| Q11G.Were you wearing a cap, hat or

3/4 LENGTH....... 2 | sun visor?

|

ELBOW LENGTH..... 3 | HAT.............. 1

|

SHORT............ 4 | CAP.............. 2

|

SLEEVELESS....... 5 | VISOR............ 3

|

IF TROUSERS/JEANS/SHORTS/SKIRT/DRESS/ | NONE WORN........ 4

WETSUIT (CODE 1 ON Q11A2) ASK |

|

Q11C.How long were/was your | IF HAT OR CAP WORN (CODES 1 OR 2 ON

trousers/jeans/shorts/skirt/dress/ | Q11G), ASK:

wetsuit? |

IF RESPONDENT HESITATES, READ OUT: | Q11H.Did your %600,/hat/cap/ have a

| wide brim or a narrow brim?

ANKLE LENGTH..... 1 |

| WIDE BRIM........ 1

3/4 LENGTH....... 2 |

| NARROW BRIM...... 2

KNEE LENGTH...... 3 |

| NO BRIM.......... 3

MINI SKIRT/SHORT |

SHORTS........... 4 | IF HAT, CAP OR VISOR WORN (CODES 1 OR

| 2 OR 3 ON Q11G), ASK:

IF SWIMWEAR (CODE 2 ON Q11A1 OR CODE 2 |

ON Q11A2), ASK: | Q11H2. Did it have a flap which

| covered the back of your neck?

Q11D.What sort of swimwear/bathers |

were you wearing? | YES.............. 1

|

ONE PIECE BATHERS 1 | NO............... 2

|

TWO PIECE/BIKINI. 2 | ASK OR RECORD:

| Q11I.Were you wearing any sunglasses?

BIKINI TOP ONLY.. 3 |

| YES.............. 1

BIKINI BOTTOM |

ONLY............. 4 | NO............... 2

|

LONG/BOARD SHORTS 5 | Q12. Did you do anything else out of

| doors on Sunday between 11am and 3pm?

SHORT SHORTS..... 6 |

| YES.............. 1

BRIEFS/SPEEDOS... 7 |

| NO............... 2

ASK OR RECORD: |

Q11E.What sort of footwear if any were | IF DID A SECOND ACTIVITY:

you wearing? |

IF WEARING SHOES OR SANDALS, ASK: Were | Q13. What other activity were you

you wearing socks/stockings with your | DOING MOSTLY?

shoes/sandals or not? |

|

THONGS........... 1 | IF OTHER, HIGHLIGHT OTHER AND TYPE IN

| RESPONSE

SANDALS WITHOUT |

SOCKS/STOCKINGS.. 2 | OTHER (SPECIFY).. 997

|

SANDALS WITH | CAN'T SAY........ 998

SOCKS............ 3 |

| Q14. About how much time did you spend

SANDALS WITH | out of doors between 11am and 3pm %O

STOCKINGS........ 4 | %152.?

| TYPE IN THE TOTAL IN MINUTES.

SHOES WITHOUT |

SOCKS/STOCKINGS.. 5 | IF CAN'T SAY ESC D

|

SHOES WITH SOCKS. 6 | |__|__|__+

|

SHOES WITH | Q15A.Were you in the Melbourne

STOCKINGS........ 7 | metropolitan area when you were %O

| %152.?

NONE WORN........ 8 |

| YES.............. 1

IF WITH SOCKS (CODES 3 OR 6 ON Q11E), |

ASK: | NO............... 2

SUN PROTECTION SURVEY 2000/2001

(C) Roy Morgan Research Pty Ltd. 2000.

DATE 29-NOV-00 SUN PROTECTION SURVEY 2000/2001 PAGE 5

________________________________________________________________________________

Q16A.While you were %O %152., was it | Q17C. How long were/was your

MOSTLY SUNNY OR MOSTLY CLOUDY? | trousers/jeans/shorts/skirt/

| dress/wetsuit?

MOSTLY SUNNY..... 1 |

| IF RESPONDENT HESITATES, READ OUT:

SOMETHING IN |

BETWEEN | ANKLE LENGTH..... 1

HAZY/LIGHT |

CLOUDY/HALF SUNNY | 3/4 LENGTH....... 2

AND HALF CLOUDY.. 2 |

| KNEE LENGTH...... 3

MOSTLY CLOUDY.... 3 |

| MINI SKIRT/SHORT

CAN'T SAY........ 4 | SHORTS........... 4

|

| IF SWIMWEAR (CODE 2 ON Q17A1 OR CODE 2

Q16B.Were you MOSTLY IN THE SHADE or | ON Q17A2) ASK:

MOSTLY OUT IN THE OPEN while you were |

%O %152.? | Q17D. What sort of swimwear/bathers

| were you wearing?

IN THE SHADE..... 1 |

| ONE PIECE BATHERS 1

IN THE OPEN...... 2 |

| TWO PIECE/BIKINI. 2

IN SHADE AND OUT |

IN OPEN EQUALLY.. 3 | BIKINI TOP ONLY.. 3

|

CAN'T SAY........ 4 | BIKINI BOTTOM

| ONLY............. 4

|

Q17A1.Would you mind telling me what | LONG/BOARD SHORTS 5

you were wearing while you were %O |

%152.? | SHORT SHORTS..... 6

|

IF RESPONDENT HESITATES PROMPT | BRIEFS/SPEEDOS... 7

WITH:Can you remember what you were |

wearing on the top part of your body? | ASK OR RECORD:

| Q17E.What sort of footwear if any were

RETURN FOR CLOTHING WORN ON LOWER PART | you wearing?

OF BODY | IF WEARING SHOES OR SANDALS, ASK: Were

| you wearing socks/stockings with your

TOP/DRESS/WETSUIT 1 | shoes/sandals or not?

|

SWIMWEAR......... 2 | THONGS........... 1

|

TOPLESS.......... 3 | SANDALS WITHOUT

| SOCKS/STOCKINGS.. 2

|

Q17A2.CLOTHING WORN ON LOWER PART OF | SANDALS WITH

BODY | SOCKS............ 3

|

RECORD OR IF RESPONDENT HESITATES | SANDALS WITH

PROMPT WITH: | STOCKINGS........ 4

Can you remember what you were wearing |

on the lower part of your body? | SHOES WITHOUT

| SOCKS/STOCKINGS.. 5

TROUSERS/JEANS/S- |

HORTS/SKIRT/DRESS | SHOES WITH SOCKS. 6

/WETSUIT......... 1 |

| SHOES WITH

SWIMWEAR......... 2 | STOCKINGS........ 7

|

BOTTOMLESS....... 3 | NONE WORN........ 8

|

| IF WITH SOCKS (CODES 3 OR 6 ON Q17E),

IF TOP/DRESS/WETSUIT (CODE 1 ON | ASK:

Q17A1), ASK: |

| Q17F.What length were the socks?

Q17B. How long were the sleeves of |

your top/dress/wetsuit? | KNEE............. 1

IF RESPONDENT HESITATES, READ OUT: |

| ANKLE............ 2

WRIST LENGTH..... 1 |

| ASK OR RECORD:

3/4 LENGTH....... 2 | Q17G.Were you wearing a cap, hat or

| sun visor?

ELBOW LENGTH..... 3 |

| HAT.............. 1

SHORT............ 4 |

| CAP.............. 2

SLEEVELESS....... 5 |

| VISOR............ 3

IF TROUSERS/JEANS/SHORTS/SKIRT/DRESS/ |

WETSUIT (CODE 1 ON Q17A2), ASK: | NONE WORN........ 4

SUN PROTECTION SURVEY 2000/2001

(C) Roy Morgan Research Pty Ltd. 2000.

DATE 29-NOV-00 SUN PROTECTION SURVEY 2000/2001 PAGE 6

________________________________________________________________________________

IF HAT OR CAP WORN (CODES 1 OR 2 ON | Q26C.Did you use a different sunscreen

Q17G), ASK: | on your face from the one you just

| mentioned?

|

Q17H.Did your %618,/hat/cap/ have a | YES, USED A

wide brim or a narrow brim? | DIFFERENT

| SUNSCREEN........ 1

WIDE BRIM........ 1 |

| USED SAME

NARROW BRIM...... 2 | SUNSCREEN........ 2

|

NO BRIM.......... 3 | DIDN'T USE ON

| FACE............. 3

|

IF HAT, CAP OR VISOR WORN (CODES 1 OR | IF DIFFERENT SUNSCREEN USED ON FACE

2 OR 3 ON Q17G), ASK: | (CODE 1 ON Q26C)

|

| Q26D. Was that another brand of

Q17H2. Did it have a flap which | sunscreen, makeup with a sunscreen or

covered the back of your neck? | moisturiser with a sunscreen?

|

YES.............. 1 | IF OTHER, HIGHLIGHT OTHER AND TYPE IN

| RESPONSE

NO............... 2 |

| Another brand of

| sunscreen........ 1

ASK OR RECORD: |

Q17I.Were you wearing any sunglasses? | Makeup with a

| sunscreen........ 2

YES.............. 1 |

| Moisturiser with

NO............... 2 | a sunscreen...... 3

|

Q24. Now some questions about | If unsure (ENTER

sunscreen. | PRODUCT NAME).... 97

A sunscreen is a gel, lotion or cream |

that filters out ultraviolet sunlight | CAN'T SAY........ 98

to prevent burning and other skin |

damage. | Q26E.What was the sun protection

Did you use a sunscreen between 11am | factor of the sunscreen you used on

and 3pm on Sunday? | your face?

| IF RESPONDENT SAYS "15"+ ENTER "16".

YES, USED | IF RESPONDENT SAYS "30"+ ENTER "31".

SUNSCREEN........ 1 | IF "DON'T KNOW" ESCAPE D.

|

NO, DIDN'T USE | |__|__+

SUNSCREEN........ 2 |

| Q27. On what parts of the body did you

MAKEUP WITH A | apply sunscreen?

SUNSCREEN ONLY... 3 | Where else?

| Anywhere else?

IF USED SUNSCREEN ASK: | PROMPT:Did you apply sunscreen to

Q25A.Did you apply the sunscreen: | your...

(READ OUT) | READ OUT LIST EXCLUDING ANSWERS

IF RESPONDENT ANSWERS "as soon as I | ALREADY GIVEN

went out in the sun" ENTER AS '2' |

| FACE............. 1,

Before going out |

in the sun....... 1 | NOSE............. 2,

|

After you'd been | (DO NOT READ)

in the sun a | HEAD............. 3,

while............ 2 |

| (DO NOT READ)

IF APPLIED AFTER BEING IN SUN (CODE 2 | EARS............. 4,

ON Q25A), ASK |

| CHEST............ 5,

Q25B.About how long after you went out |

in the sun did you apply the | STOMACH.......... 6,

sunscreen? |

IF RESPONDENT ANSWERED "as soon as I | BACK............. 7,

went out in the sun" ENTER '0' MINUTES |

| NECK............. 8,

|

IF CAN'T SAY ESC D | SHOULDERS........ 9,

|

|__|__|__+ | ARMS............. 10,

|

Q26B.What was the sun protection | HANDS............ 11,

factor of the suncreen you used? |

IF RESPONDENT SAYS "15"+ ENTER "16". | LEGS............. 12,

IF RESPONDENT SAYS "30"+ ENTER "31". |

IF "DON'T KNOW" ESCAPE D. | BACK OF KNEES.... 13,

|

|__|__+ | FEET............. 14,

SUN PROTECTION SURVEY 2000/2001

(C) Roy Morgan Research Pty Ltd. 2000.

DATE 29-NOV-00 SUN PROTECTION SURVEY 2000/2001 PAGE 7

________________________________________________________________________________

Q28. Were there any areas exposed to | OTHER (SPECIFY).. 997

the sun that didn't have sunscreen on |

them? | CAN'T SAY........ 998

|

FACE............. 1, | Q31. About how much time did you spend

| out of doors on Saturday between 11am

NOSE............. 2, | and 3pm %O %180.?

|

HEAD............. 3, | TYPE IN THE TOTAL IN MINUTES.

|

EARS............. 4, | IF STILL CAN'T SAY ESC D

|

CHEST............ 5, | |__|__|__+

|

STOMACH.......... 6, | Q32A. Were you in the Melbourne

| metropolitan area when you were %O

BACK............. 7, | %180.?

|

NECK............. 8, | YES.............. 1

|

SHOULDERS........ 9, | NO............... 2

|

ARMS............. 10, | Q33A. While you were %O %180., was it

| MOSTLY SUNNY OR MOSTLY CLOUDY?

HANDS............ 11, |

| MOSTLY SUNNY..... 1

LEGS............. 12, |

| SOMETHING IN

BACK OF KNEES.... 13, | BETWEEN

| HAZY/LIGHT

FEET............. 14, | CLOUDY/HALF SUNNY

| AND HALF CLOUDY.. 2

NONE............. 15, |

| MOSTLY CLOUDY.... 3

ASK EVERYONE: |

Q6C. Between 11:00am and 3:00pm on | CAN'T SAY........ 4

Sunday did you at any time CHOOSE to |

stay out of the sun so as not to get | Q33B. Were you MOSTLY IN THE SHADE or

too much sun? | MOSTLY OUT IN THE OPEN while you were

| %O %180.?

YES.............. 1 |

| IN THE SHADE..... 1

NO............... 2 |

| IN THE OPEN...... 2

Q6A. Thinking about the WHOLE day on |

Sunday - not just the time between | IN SHADE AND OUT

11:00am and 3:00pm - about how much | IN OPEN EQUALLY.. 3

time did you spend out of doors? By |

out of doors we mean not in a building | CAN'T SAY........ 4

and not in a covered vehicle. |

TYPE IN TOTAL IN MINUTES! | Q34A1.Would you mind telling me what

IF CAN'T SAY, ASK: Well, could you | you were wearing while you were %O

estimate to the NEAREST 1/2 HOUR how | %180.?

much time you spent out of doors on |

Sunday? | IF RESPONDENT HESITATES, SAY: Can you

TYPE TIME IN MINUTES | remember what you were wearing on the

| top part of your body?

|

IF STILL CAN'T SAY ESC D | RETURN FOR CLOTHING WORN ON LOWER PART

| OF BODY

|__|__|__+ |

| TOP/DRESS/WETSUIT 1

Q29B.Thinking back to Saturday. Were |

you out of doors for longer than 15 | SWIMWEAR......... 2

minutes between 11am and 3pm? |

| TOPLESS.......... 3

IF INTERMITTENTLY OUTDOORS ASK: Would |

you say you were actually out of doors | Q34A2.CLOTHING WORN ON LOWER PART OF

for longer than 15 minutes in total? | BODY

|

YES.............. 1 | RECORD OR IF RESPONDENT HESITATES

| PROMPT WITH:

NO............... 2 | Can you remember what you were wearing

| on the lower part of your body?

IF YES ON SATURDAY: |

| TROUSERS/JEANS/S-

Q30. What activity were you DOING | HORTS/SKIRT/DRESS

MOSTLY during that time out of doors? | /WETSUIT......... 1

|

| SWIMWEAR......... 2

IF OTHER, HIGHLIGHT OTHER AND TYPE IN |

RESPONSE | BOTTOMLESS....... 3

SUN PROTECTION SURVEY 2000/2001

(C) Roy Morgan Research Pty Ltd. 2000.

DATE 29-NOV-00 SUN PROTECTION SURVEY 2000/2001 PAGE 8

________________________________________________________________________________

IF TOP/DRESS/WETSUIT (CODE 1 ON | Q34F.What length were the socks?

Q34A1), ASK |

| KNEE............. 1

Q34B.How long were the sleeves of your |

top/dress/wetsuit? |

IF RESPONDENT HESITATES, READ OUT: | ANKLE............ 2

|

WRIST LENGTH..... 1 | ASK OR RECORD:

| Q34G.Were you wearing a cap, hat or

3/4 LENGTH....... 2 | sun visor?

|

ELBOW LENGTH..... 3 | HAT.............. 1

|

SHORT............ 4 | CAP.............. 2

|

SLEEVELESS....... 5 | VISOR............ 3

|

IF TROUSERS/JEANS/SHORTS/SKIRT/DRESS/ | NONE WORN........ 4

WETSUIT (CODE 1 ON Q34A2), ASK |

| IF HAT OR CAP WORN (CODES 1 OR 2 ON

Q34C.How long were/was your trousers/ | Q34G), ASK

jeans/shorts/skirt/dress /wetsuit? |

| Q34H.Did your %640,/hat/cap/ have a

IF RESPONDENT HESITATES, READ OUT: | wide brim or a narrow brim?

|

ANKLE LENGTH..... 1 | WIDE BRIM........ 1

|

3/4 LENGTH....... 2 | NARROW BRIM...... 2

|

KNEE LENGTH...... 3 | NO BRIM.......... 3

|

MINI SKIRT/SHORT | IF HAT, CAP OR VISOR WORN (CODE 1 OR 2

SHORTS........... 4 | OR 3 ON Q34G), ASK

|

IF SWIMWEAR (CODE 2 ON Q34A1 OR CODE 2 | Q34H2. Did it have a flap which

ON Q34A2), ASK | covered the back of your neck?

|

Q34D.What sort of swimwear/bathers | YES.............. 1

were you wearing? |

| NO............... 2

ONE PIECE BATHERS 1 |

| ASK OR RECORD:

TWO PIECE/BIKINI. 2 | Q34I.Were you wearing any sunglasses?

|

BIKINI TOP ONLY.. 3 | YES.............. 1

|

BIKINI BOTTOM | NO............... 2

ONLY............. 4 |

| Q35. Did you do anything else out of

LONG/BOARD SHORTS 5 | doors on Saturday between 11am and

| 3pm?

SHORT SHORTS..... 6 |

| YES.............. 1

BRIEFS/SPEEDOS... 7 |

| NO............... 2

ASK OR RECORD: |

Q34E.What sort of footwear if any were | IF DID A SECOND ACTIVITY:

you wearing? |

IF WEARING SHOES OR SANDALS, ASK: Were | Q36. What other activity were you

you wearing socks/stockings with your | DOING MOSTLY?

shoes/sandals or not? |

|

THONGS........... 1 | IF OTHER, HIGHLIGHT OTHER AND TYPE IN

| RESPONSE

SANDALS WITHOUT |

SOCKS/STOCKINGS.. 2 | OTHER (SPECIFY).. 997

|

SANDALS WITH | CAN'T SAY........ 998

SOCKS............ 3 |

| Q37. About how much time did you spend

SANDALS WITH | out of doors between 11am and 3pm %O

STOCKINGS........ 4 | %197.?

| TYPE IN THE TOTAL IN MINUTES.

SHOES WITHOUT |

SOCKS/STOCKINGS.. 5 | IF CAN'T SAY ESC D

|

SHOES WITH SOCKS. 6 | |__|__|__+

|

SHOES WITH | Q38A.Were you in the Melbourne

STOCKINGS........ 7 | metropolitan area when you were %O

| %197.?

NONE WORN........ 8 |

| YES.............. 1

IF WITH SOCKS (CODES 3 OR 6 ON Q34E), |

ASK | NO............... 2

SUN PROTECTION SURVEY 2000/2001

(C) Roy Morgan Research Pty Ltd. 2000.

DATE 29-NOV-00 SUN PROTECTION SURVEY 2000/2001 PAGE 9

________________________________________________________________________________

Q39A. While you were %O %197., was it | Q40C.How long were/was your trousers/

MOSTLY SUNNY OR MOSTLY CLOUDY? | jeans/shorts/skirt/dress /wetsuit?

|

MOSTLY SUNNY..... 1 | IF RESPONDENT HESITATES, READ OUT:

|

| ANKLE LENGTH..... 1

SOMETHING IN |

BETWEEN | 3/4 LENGTH....... 2

HAZY/LIGHT |

CLOUDY/HALF SUNNY | KNEE LENGTH...... 3

AND HALF CLOUDY.. 2 |

| MINI SKIRT/SHORT

| SHORTS........... 4

MOSTLY CLOUDY.... 3 |

| IF SWIMWEAR (CODE 2 ON Q40A1 OR CODE 2

CAN'T SAY........ 4 | ON Q40A2), ASK

|

Q39B.Were you MOSTLY IN THE SHADE or | Q40D.What sort of swimwear/bathers

MOSTLY OUT IN THE OPEN while you were | were you wearing?

%O %197.? |

| ONE PIECE BATHERS 1

IN THE SHADE..... 1 |

| TWO PIECE/BIKINI. 2

IN THE OPEN...... 2 |

| BIKINI TOP ONLY.. 3

IN SHADE AND OUT |

IN OPEN EQUALLY.. 3 | BIKINI BOTTOM

| ONLY............. 4

CAN'T SAY........ 4 |

| LONG/BOARD SHORTS 5

Q40A1. Would you mind telling me what |

you were wearing while you were %O | SHORT SHORTS..... 6

%197.? |

| BRIEFS/SPEEDOS... 7

IF RESPONDENT HESITATES, SAY: |

Can you remember what you were wearing | ASK OR RECORD:

on the top part of your body? | Q40E.What sort of footwear if any were

| you wearing?

| IF WEARING SHOES OR SANDALS, ASK: Were

RETURN FOR CLOTHING WORN ON LOWER PART | you wearing socks/stockings with your

OF BODY | shoes/sandals or not?

|

TOP/DRESS/WETSUIT 1 | THONGS........... 1

|

SWIMWEAR......... 2 | SANDALS WITHOUT

| SOCKS/STOCKINGS.. 2

TOPLESS.......... 3 |

| SANDALS WITH

Q40A2.CLOTHING WORN ON LOWER PART OF | SOCKS............ 3

BODY |

| SANDALS WITH

RECORD OR IF RESPONDENT HESITATES | STOCKINGS........ 4

PROMPT WITH: |

Can you remember what you were wearing | SHOES WITHOUT

on the lower part of your body? | SOCKS/STOCKINGS.. 5

|

TROUSERS/JEANS/S- | SHOES WITH SOCKS. 6

HORTS/SKIRT/DRESS |

/WETSUIT......... 1 | SHOES WITH

| STOCKINGS........ 7

SWIMWEAR......... 2 |

| NONE WORN........ 8

BOTTOMLESS....... 3 |

| IF WITH SOCKS (CODES 3 OR 6 ON Q40E),

IF TOP/DRESS/WETSUIT (CODE 1 ON Q40A1) | ASK

ASK |

| Q40F.What length were the socks?

Q40B.How long were the sleeves of your |

top/dress/wetsuit? | KNEE............. 1

IF RESPONDENT HESITATES, READ OUT: |

| ANKLE............ 2

WRIST LENGTH..... 1 |

| ASK OR RECORD:

3/4 LENGTH....... 2 | Q40G.Were you wearing a cap, hat or

| sun visor?

ELBOW LENGTH..... 3 |

| HAT.............. 1

SHORT............ 4 |

| CAP.............. 2

SLEEVELESS....... 5 |

| VISOR............ 3

IF TROUSERS/JEANS/SHORTS/SKIRT/DRESS/ |

WETSUIT (CODE 1 ON Q40A2), ASK | NONE WORN........ 4

SUN PROTECTION SURVEY 2000/2001

(C) Roy Morgan Research Pty Ltd. 2000.

DATE 29-NOV-00 SUN PROTECTION SURVEY 2000/2001 PAGE 10

________________________________________________________________________________

IF HAT OR CAP WORN (CODE 1 OR 2 ON | Q49C.Did you use a different sunscreen

Q40G), ASK | on your face from the one you just

| mentioned?

|

Q40H.Did your %656,/hat/cap/ have a | YES, USED A

wide brim or a narrow brim? | DIFFERENT

| SUNSCREEN........ 1

WIDE BRIM........ 1 |

| USED SAME

| SUNSCREEN........ 2

NARROW BRIM...... 2 |

| DIDN'T USE ON

| FACE............. 3

NO BRIM.......... 3 |

| IF DIFFERENT SUNSCREEN USED ON FACE

| (CODE 1 ON Q49C)

IF HAT, CAP OR VISOR WORN (CODE 1 OR 2 |

OR 3 ON Q40G), ASK | Q49D. Was that another brand of

| sunscreen, makeup with a sunscreen or

| moisturiser with a sunscreen?

Q40H2. Did it have a flap which |

covered the back of your neck? |

| IF OTHER, HIGHLIGHT OTHER AND TYPE IN

YES.............. 1 | RESPONSE

|

NO............... 2 | Another brand of

| sunscreen........ 1

ASK OR RECORD: |

Q40I.Were you wearing any sunglasses? | Makeup with a

| sunscreen........ 2

YES.............. 1 |

| Moisturiser with

NO............... 2 | a sunscreen...... 3

|

Q47. Now some questions about | If unsure (ENTER

sunscreen. | PRODUCT NAME).... 97

A sunscreen is a gel, lotion or cream |

that filters out ultraviolet sunlight | CAN'T SAY........ 98

to prevent burning and other skin |

damage. | Q49E. What was the sun protection

Did you use a sunscreen between 11am | factor of the sunscreen you used on

and 3pm on Saturday? | your face?

|

YES, USED | IF RESPONDENT SAYS "15"+ ENTER "16".

SUNSCREEN........ 1 | IF RESPONDENT SAYS "30"+ ENTER "31".

| IF "DON'T KNOW" ESCAPE D.

NO, DIDN'T USE |

SUNSCREEN........ 2 | |__|__+

|

MAKEUP WITH A | Q50A. On what parts of the body did

SUNSCREEN ONLY... 3 | you apply sunscreen?

| Where else?

IF USED SUNSCREEN ASK | Anywhere else?

Q48A.Did you apply the sunscreen: |

(READ OUT) | PROMPT: Did you apply sunscreen to

IF RESPONDENT ANSWERS "as soon as I | your... READ OUT LIST EXCLUDING

went out in the sun" ENTER AS '2' | ANSWERS GIVEN

|

Before going out | FACE............. 1,

in the sun....... 1 |

| NOSE............. 2,

After you'd been |

in the sun a | (DO NOT READ)

while............ 2 | HEAD............. 3,

|

IF APPLIED AFTER BEING IN SUN | (DO NOT READ)

| EARS............. 4,

Q48B.About how long after you went out |

in the sun did you apply the | CHEST............ 5,

sunscreen? |

IF RESPONDENT ANSWERED "as soon as I | STOMACH.......... 6,

went out in the sun" ENTER '0' MINUTES |

| BACK............. 7,

|

IF CAN'T SAY ESC D | NECK............. 8,

|

|__|__|__+ | SHOULDERS........ 9,

|

Q49B.What was the sun protection | ARMS............. 10,

factor of the suncreen you used? |

IF RESPONDENT SAYS "15"+ ENTER "16". | HANDS............ 11,

IF RESPONDENT SAYS "30"+ ENTER "31". |

IF "DON'T KNOW" ESCAPE D. | LEGS............. 12,

|

|__|__+ | BACK OF KNEES.... 13,

SUN PROTECTION SURVEY 2000/2001

(C) Roy Morgan Research Pty Ltd. 2000.

DATE 29-NOV-00 SUN PROTECTION SURVEY 2000/2001 PAGE 11

________________________________________________________________________________

FEET............. 14, | Q51B. And would you describe it

| as...(READ OUT)

Q50B.Were there any areas exposed to |

the sun that didn't have sunscreen on | Wet.............. 1

them? |

| Humid or......... 2

FACE............. 1, |

| Dry.............. 3

NOSE............. 2, |

|

HEAD............. 3, |

|

EARS............. 4, | Q51C. Did these conditions

| feel...(READ OUT)

CHEST............ 5, |

| Very comfortable. 1

STOMACH.......... 6, |

| Comfortable...... 2

BACK............. 7, |

| Uncomfortable or. 3

NECK............. 8, |

| Very

SHOULDERS........ 9, | uncomfortable.... 4

|

ARMS............. 10, |

|

HANDS............ 11, |

| +------------------------------------+

LEGS............. 12, | | I am now going to read out a |

| | number of statements. For each |

BACK OF KNEES.... 13, | | statement, I'd like to know how |

| | much you agree or disagree. |

FEET............. 14, | +------------------------------------+

|

NONE............. 15, |

|

ASK EVERYONE: |

Q29C. Between 11:00am and 3:00pm on | Q52A.Do you agree or disagree with the

Saturday did you at any time CHOOSE to | statement "I feel more healthy with a

stay out of the sun so as not to get | suntan"?

too much sun? |

| IF AGREE OR DISAGREE, ASK: Is that

YES.............. 1 | strongly agree/disagree or mildly

| agree/disagree?

NO............... 2 |

| STRONGLY DISAGREE 1

Q29A. Thinking about the WHOLE day on |

Saturday not just the time between | MILDLY DISAGREE.. 2

11:00am and 3:00pm - about how much |

time did you spend out of doors? By | NEITHER AGREE NOR

out of doors we mean not in a building | DISAGREE......... 3

and not in a covered vehicle. |

TYPE IN TOTAL IN MINUTES! | MILDLY AGREE..... 4

IF CAN'T SAY, ASK: Well, could you |

estimate to the NEAREST 1/2 HOUR how | STRONGLY AGREE... 5

much time you spent out of doors on |

Saturday? | CAN'T SAY........ 6

TYPE TIME IN MINUTES |

IF STILL CAN'T SAY ESC D |

|

|__|__|__+ | Q52B.Do you agree or disagree with the

| statement "I take great care to avoid

+------------------------------------+ | getting sunburnt".

| The following question is | |

| concerned with SUNDAY rather than | | IF AGREE OR DISAGREE ASK: Is that

| SATURDAY. | | strongly agree/disagree or mildly

+------------------------------------+ | agree/disagree?

|

Q51A. Now, thinking about the weather | STRONGLY DISAGREE 1

conditions between 11am and 3pm on |

SUNDAY. Would you describe them | MILDLY DISAGREE.. 2

as...(READ OUT) |

| NEITHER AGREE NOR

Cold............. 1 | DISAGREE......... 3

|

Cool............. 2 | MILDLY AGREE..... 4

|

Warm or.......... 3 | STRONGLY AGREE... 5

|

Hot.............. 4 | CAN'T SAY........ 6

SUN PROTECTION SURVEY 2000/2001

(C) Roy Morgan Research Pty Ltd. 2000.

DATE 29-NOV-00 SUN PROTECTION SURVEY 2000/2001 PAGE 12

________________________________________________________________________________

Q52C.Do you agree or disagree with the | Q52G.Do you agree or disagree with the

statement "A suntanned person looks | statement "Once you get a suntan it's

more healthy". | easier to enjoy the summer months".

|

IF AGREE OR DISAGREE ASK: Is that | IF AGREE OR DISAGREE ASK: Is that

strongly agree/disagree or mildly | strongly agree/disagree or mildly

agree/disagree? | agree/disagree?

|

STRONGLY DISAGREE 1 | STRONGLY DISAGREE 1

|

MILDLY DISAGREE.. 2 | MILDLY DISAGREE.. 2

|

NEITHER AGREE NOR | NEITHER AGREE NOR

DISAGREE......... 3 | DISAGREE......... 3

|

MILDLY AGREE..... 4 | MILDLY AGREE..... 4

|

STRONGLY AGREE... 5 | STRONGLY AGREE... 5

|

CAN'T SAY........ 6 | CAN'T SAY........ 6

|

Q52D.Do you agree or disagree with the | Q52H.Do you agree or disagree with the

statement "It's worth a lot of effort | statement "Most of my close family

to get a suntan". | think that a suntan is a good thing".

|

IF AGREE OR DISAGREE ASK: Is that | IF AGREE OR DISAGREE ASK: Is that

strongly agree/disagree or mildly | strongly agree/disagree or mildly

agree/disagree? | agree/disagree?

|

STRONGLY DISAGREE 1 | STRONGLY DISAGREE 1

|

MILDLY DISAGREE.. 2 | MILDLY DISAGREE.. 2

|

NEITHER AGREE NOR | NEITHER AGREE NOR

DISAGREE......... 3 | DISAGREE......... 3

|

MILDLY AGREE..... 4 | MILDLY AGREE..... 4

|

STRONGLY AGREE... 5 | STRONGLY AGREE... 5

|

CAN'T SAY........ 6 | CAN'T SAY........ 6

|

Q52E.Do you agree or disagree with the | Q52I.Do you agree or disagree with the

statement "A suntanned person is more | statement "I find it difficult to

healthy". | protect myself from the sun".

|

IF AGREE OR DISAGREE ASK: Is that | IF AGREE OR DISAGREE ASK: Is that

strongly agree/disagree or mildly | strongly agree/disagree or mildly

agree/disagree? | agree/disagree?

|

STRONGLY DISAGREE 1 | STRONGLY DISAGREE 1

|

MILDLY DISAGREE.. 2 | MILDLY DISAGREE.. 2

|

NEITHER AGREE NOR | NEITHER AGREE NOR

DISAGREE......... 3 | DISAGREE......... 3

|

MILDLY AGREE..... 4 | MILDLY AGREE..... 4

|

STRONGLY AGREE... 5 | STRONGLY AGREE... 5

|

CAN'T SAY........ 6 | CAN'T SAY........ 6

|

Q52F.Do you agree or disagree with the | Q52J.Do you agree or disagree with the

statement "A lot of sun throughout | statement "Most of my friends think a

life ages the skin". | suntan is a good thing".

|

IF AGREE OR DISAGREE ASK: Is that | IF AGREE OR DISAGREE ASK: Is that

strongly agree/disagree or mildly | strongly agree/disagree or mildly

agree/disagree? | agree/disagree?

|

STRONGLY DISAGREE 1 | STRONGLY DISAGREE 1

|

MILDLY DISAGREE.. 2 | MILDLY DISAGREE.. 2

|

NEITHER AGREE NOR | NEITHER AGREE NOR

DISAGREE......... 3 | DISAGREE......... 3

|

MILDLY AGREE..... 4 | MILDLY AGREE..... 4

|

STRONGLY AGREE... 5 | STRONGLY AGREE... 5

|

CAN'T SAY........ 6 | CAN'T SAY........ 6

SUN PROTECTION SURVEY 2000/2001

(C) Roy Morgan Research Pty Ltd. 2000.

DATE 29-NOV-00 SUN PROTECTION SURVEY 2000/2001 PAGE 13

________________________________________________________________________________

Q52K.Do you agree or disagree with the | Q52O.Do you agree or disagree with the

statement "Skin cancer is a dangerous | statement "In summer sunshine I

disease". | usually take care to put on some

| sunscreen".

IF AGREE OR DISAGREE ASK: Is that |

strongly agree/disagree or mildly | IF AGREE OR DISAGREE ASK: Is that

agree/disagree? | strongly agree/disagree or mildly

| agree/disagree?

STRONGLY DISAGREE 1 |

| STRONGLY DISAGREE 1

MILDLY DISAGREE.. 2 |

| MILDLY DISAGREE.. 2

NEITHER AGREE NOR |

DISAGREE......... 3 | NEITHER AGREE NOR

| DISAGREE......... 3

MILDLY AGREE..... 4 |

| MILDLY AGREE..... 4

STRONGLY AGREE... 5 |

| STRONGLY AGREE... 5

CAN'T SAY........ 6 |

| CAN'T SAY........ 6

Q52L.Do you agree or disagree with the |

statement "There is little chance that |

I'll get skin cancer". |

|

IF AGREE OR DISAGREE ASK: Is that | Q52P.Do you agree or disagree with the

strongly agree/disagree or mildly | statement "I need to do more to

agree/disagree? | protect myself from the sun because of

| the hole in the Ozone layer"?

STRONGLY DISAGREE 1 |

| IF AGREE OR DISAGREE ASK: Is that

MILDLY DISAGREE.. 2 | strongly agree/disagree or mildly

| agree/disagree?

NEITHER AGREE NOR |

DISAGREE......... 3 | STRONGLY DISAGREE 1

|

MILDLY AGREE..... 4 | MILDLY DISAGREE.. 2

|

STRONGLY AGREE... 5 | NEITHER AGREE NOR

| DISAGREE......... 3

CAN'T SAY........ 6 |

| MILDLY AGREE..... 4

Q52M.Do you agree or disagree with the |

statement "Often in summer sunshine I | STRONGLY AGREE... 5

don't bother putting on a hat". |

| CAN'T SAY........ 6

IF AGREE OR DISAGREE ASK: Is that |

strongly agree/disagree or mildly |

agree/disagree? |

| Q52Q.Do you agree or disagree with the

STRONGLY DISAGREE 1 | statement "I should try to avoid

| getting ANY sunlight on my skin".

MILDLY DISAGREE.. 2 |

| IF AGREE OR DISAGREE ASK: Is that

NEITHER AGREE NOR | strongly agree/disagree or mildly

DISAGREE......... 3 | agree/disagree?

|

MILDLY AGREE..... 4 | STRONGLY DISAGREE 1

|

STRONGLY AGREE... 5 | MILDLY DISAGREE.. 2

|

CAN'T SAY........ 6 | NEITHER AGREE NOR

| DISAGREE......... 3

Q52N.Do you agree or disagree with the |

statement "A suntan protects you | MILDLY AGREE..... 4

against skin cancer". |

| STRONGLY AGREE... 5

IF AGREE OR DISAGREE ASK: Is that |

strongly agree/disagree or mildly | CAN'T SAY........ 6

agree/disagree? |

|

STRONGLY DISAGREE 1 |

| IF SUNBURNT (SINGLE RESPONSE ON Q4B OR

MILDLY DISAGREE.. 2 | RESPONSE ON Q4C)

|

NEITHER AGREE NOR |

DISAGREE......... 3 |

| +------------------------------------+

MILDLY AGREE..... 4 | | Returning to the issue of sunburn. |

| | I want to find out a little bit |

STRONGLY AGREE... 5 | | more about how much of your body |

| | got sunburnt on the weekend. |

CAN'T SAY........ 6 | +------------------------------------+

SUN PROTECTION SURVEY 2000/2001

(C) Roy Morgan Research Pty Ltd. 2000.

DATE 29-NOV-00 SUN PROTECTION SURVEY 2000/2001 PAGE 14

________________________________________________________________________________

QAA1. %671,/You said your worst | Q53A.Which of the following statements

sunburn was on your/ You said you were | best describes your moles or freckles?

sunburnt on your/ %130.. |

| (READ OUT)

Were you burnt over...READ OUT |

| They cover most

Most of your | of my body....... 1

%130............. 1 |

| I have a lot..... 2

About half of |

your %130........ 2 | I have a few..... 3

|

Well under half.. 3 | I have very few

| or none.......... 4

Or only on a |

small strip...... 4 | (DO NOT READ)

| DON'T KNOW/CAN'T

(DO NOT READ) | SAY.............. 5

DON'T KNOW/ CAN'T |

SAY.............. 5 |

| +------------------------------------+

IF SUNBURNT (SINGLE RESPONSE ON Q4B OR | | Now I am going to ask you a |

RESPONSE ON Q4C) | | question about advertisements on |

| | television. |

QAB7. During the time you got sunburnt | +------------------------------------+

on your %130., were you trying to |

protect your %130.? |

| Q53C2. Do you remember ever seeing an

YES.............. 1 | advertisement on TV which showed

| scenes of a young man with a large

NO............... 2 | mole on his neck? Do you remember ever

| seeing this advertisement?

IF TRYING TO PROTECT BODY PART FROM |

SUN (CODE 1 ON QAB7) ASK: | YES.............. 1

|

QAB7A. How do you think you became | NO............... 2

sunburnt on your %130.? |

| CAN'T SAY........ 3

IF UNSURE READ OUT. |

|

DIDN'T PROTECT | +------------------------------------+

STRAIGHT AWAY OR | | Thinking now about the daily |

ALL THE TIME..... 1 | | weather reports and forecasts in |

| | the news on television, radio and |

MISSED AREA WHEN | | in newspapers. |

APPLIED SUNSCREEN 2 | +------------------------------------+

|

SUNSCREEN WORE | QD1. Did the weather forecast

OFF.............. 3 | influence your plans for spending time

| outside last weekend?

BURNT THROUGH |

SUNSCREEN........ 4 | IF NO, SAY: Did the forecast not

| influence your plans, or did you

BURNT THROUGH | simply not see the forecast?

CLOTHING......... 5 |

| YES.............. 1

CAN'T SAY........ 6 |

| NO, NO CHANGE IN

IF NOT TRYING TO PROTECT BODY PART | PLANS............ 2

FROM SUN (CODE 2 ON QAB7) ASK: |

| NO, DIDN'T SEE OR

QAB7B. Why didn't you protect your | HEAR WEATHER

%130.? | FORECAST......... 3

Was it because you...READ OUT |

| CAN'T SAY........ 4

Forgot........... 1 |

| IF WEATHER FORECAST INFLUENCED YOUR

Couldn't be | PLANS (CODE 1 ON QD1)

bothered......... 2 |

| QDA1. Did you plan to spend more time

Didn't think | outside or less time outside?

needed to........ 3 |

| PLANNED TO SPEND

(DO NOT READ) | MORE TIME OUTSIDE 1

CAN'T SAY........ 4 |

| PLANNED TO SPEND

+------------------------------------+ | LESS TIME OUTSIDE 2

| ASK EVERYONE. | |

+------------------------------------+ | CAN'T SAY........ 3

SUN PROTECTION SURVEY 2000/2001

(C) Roy Morgan Research Pty Ltd. 2000.

DATE 29-NOV-00 SUN PROTECTION SURVEY 2000/2001 PAGE 15

________________________________________________________________________________

IF CAN'T SAY WHETHER WEATHER FORECAST | QD2. Thinking about the weekend just

INFLUENCED YOUR PLANS (CODE 4 ON QD1) | gone, did you notice the UV forecasts

or YOU PLANNED TO SPEND MORE OR LESS | for Saturday?

TIME OUTSIDE OR CAN'T SAY AFTER |

HEARING OR SEEING THE WEATHER FORECAST | YES.............. 1

(CODES 1 OR 2 OR 3 ON QDA1), ASK |

| NO............... 2

QD1A. Did the forecast lead you to |

take more or less precautions to | CAN'T SAY........ 3

protect yourself from the sun when you |

were out, or did it have no effect? |

| QD3. Thinking about the weekend just

MORE............. 1 | gone, did you notice the UV forecasts

| for Sunday?

LESS............. 2 |

| YES.............. 1

NO EFFECT........ 3 |

| NO............... 2

CAN'T SAY........ 4 |

| CAN'T SAY........ 3

IF MORE PRECAUTIONS TAKEN BECAUSE OF |

FORECAST (CODE 1 ON QD1A) ASK: |

| Q61. In the past 12 months have you

QD1B. What aspects of the weather | attended a solarium to get your skin

influenced you to change your sun | tanned?

protection? |

| YES.............. 1

IF OTHER, HIGHLIGHT OTHER AND TYPE IN |

RESPONSE | NO............... 2

|

TEMPERATURE...... 1, | CAN'T SAY........ 3

|

CLOUD............ 2, |

| IF ATTENDED SOLARIUM IN PAST 12 MONTHS

WIND............. 3, | (CODE 1 ON Q61) ASK:

|

RAIN............. 4, |

| Q62. How many times in the last 12

UV............... 5, | months have you attended a solarium to

| get your skin tanned?

THE SUN.......... 6, |

| 1-2 TIMES........ 1

THE HUMIDITY..... 7, |

| 3-5 TIMES........ 2

OTHER (SPECIFY).. 97, |

| MORE THAN 5 TIMES 3

CAN'T SAY........ 98, |

| CAN'T SAY........ 4

IF LESS PRECAUTIONS TAKEN BECAUSE OF |

FORECAST (CODE 2 ON QD1A) ASK: | +------------------------------------+

| | ASK EVERYONE. |

QD1C. What aspects of the weather | +------------------------------------+

influenced you to change your sun |

protection? | Q63. Do you agree or disagree with the

| statement

IF OTHER, HIGHLIGHT OTHER AND TYPE IN | "You can get a 'safer suntan' in a

RESPONSE | solarium than at the beach"?

| IF AGREE/DISAGREE ASK: Is that

TEMPERATURE...... 1, | strongly agree/disagree or mildly

| agree/disagree?

CLOUD............ 2, |

| STRONGLY DISAGREE 1

WIND............. 3, |

| MILDLY DISAGREE.. 2

RAIN............. 4, |

| NEITHER DISAGREE

UV............... 5, | NOR AGREE........ 3

|

THE SUN.......... 6, | MILDLY AGREE..... 4

|

THE HUMIDITY..... 7, | STRONGLY AGREE... 5

|

OTHER (SPECIFY).. 97, | CAN'T SAY........ 6

|

CAN'T SAY........ 98, | Q64. This spring or summer, have you

| used a cosmetic product to make you

+------------------------------------+ | look more tanned than you were? That

| ASK EVERYONE | | is, used an artificial tan or fake

+------------------------------------+ | tan?

|

+------------------------------------+ | YES.............. 1

| UV forecasts tell you the level of | |

| ultra violet radiation expected | | NO............... 2

| for the forecast period | |

+------------------------------------+ | CAN'T SAY........ 3

SUN PROTECTION SURVEY 2000/2001

(C) Roy Morgan Research Pty Ltd. 2000.

DATE 29-NOV-00 SUN PROTECTION SURVEY 2000/2001 PAGE 16

________________________________________________________________________________

Q65. Do you agree or disagree with the | Q67. Thinking about your youngest

statement | child under 16 years of age, could you

"An artificial tan you get from | tell me their age?

applying a cosmetic product protects |

you from the harmful rays of the sun"? | INFANT LESS THAN

IF AGREE/DISAGREE ASK: Is that | 1 YEAR........... 1

strongly agree/disagree or mildly |

agree/disagree? | 1 YEAR UP TO 2

| YEARS............ 2

STRONGLY DISAGREE 1 |

| 2 YEARS UP TO 3

MILDLY DISAGREE.. 2 | YEARS............ 3

|

NEITHER DISAGREE | 3 YEARS UP TO 4

NOR AGREE........ 3 | YEARS............ 4

|

MILDLY AGREE..... 4 | 4 YEARS UP TO 5

| YEARS............ 5

STRONGLY AGREE... 5 |

| 5 YEARS UP TO 6

CAN'T SAY........ 6 | YEARS............ 6

|

| 6 YEARS UP TO 7

| YEARS............ 7

|

| 7 YEARS UP TO 8

| YEARS............ 8

|

| 8 YEARS UP TO 9

| YEARS............ 9

|

| 9 YEARS UP TO 10

| YEARS............ 10

|

| 10 YEARS UP TO 11

| YEARS............ 11

|

| 11 YEARS UP TO 12

| YEARS............ 12

|

| 12 YEARS UP TO 13

| YEARS............ 13

Q66. Are you a parent or guardian of |

one or more children under 16 years of | 13 YEARS UP TO 14

age? | YEARS............ 14

|

YES.............. 1 | 14 YEARS UP TO 15

| YEARS............ 15

NO............... 2 |

| 15 YEARS UP TO 16

CAN'T SAY........ 3 | YEARS............ 16

|

| CAN'T SAY/

| REFUSED.......... 17

|

| IF YOUNGEST CHILD IS AN INFANT LESS

| THAN 1 YEAR OLD (CODE 1 ON Q67) ASK:

|

| Q68. How many months?

|

| UP TO 3 MONTHS... 1

|

| 3 MONTHS UP TO 6

| MONTHS........... 2

|

| 6 MONTHS UP TO 12

| MONTHS........... 3

|

| CAN'T SAY/

| REFUSED.......... 4

|

| +------------------------------------+

IF PARENT OR GUARDIAN OF CHILDREN | | IF PARENT OR GUARDIAN OF CHILDREN |

UNDER 16 (CODE 1 ON Q66) ASK, | | UNDER 16 (CODE 1 ON Q66) ASK: |

OTHERWISE GO TO Q53D: | +------------------------------------+

SUN PROTECTION SURVEY 2000/2001

(C) Roy Morgan Research Pty Ltd. 2000.

DATE 29-NOV-00 SUN PROTECTION SURVEY 2000/2001 PAGE 17

________________________________________________________________________________

Q69. Still thinking about your | Q74. Can you tell me the type of

youngest child, can you tell me if he | things they do to protect the children

or she got sunburnt at all on Sunday? | from the sun?

What about Saturday? | HIGHLIGHT ALL MENTIONED.

|

SUNDAY........... 1, | IF OTHER, HIGHLIGHT OTHER AND TYPE IN

| RESPONSE

SATURDAY......... 2, |

| PROVIDE SHADE

NEITHER DAY...... 3, | OUTDOORS......... 1,

|

CAN'T SAY........ 4, | PROVIDE SUNSCREEN 2,

|

| HAT-WEARING...... 3,

Q70. Still thinking about your |

youngest child, can you tell me if he | LIMIT OUTDOOR

or she was out of doors for longer | ACTIVITIES

than 15 minutes between 11am and 3pm | BETWEEN 11AM AND

on Sunday? | 3PM.............. 4,

|

YES.............. 1 | PROTECTIVE SPORTS

| UNIFORM.......... 5,

NO............... 2 |

| HAVE A SUN

CAN'T SAY........ 3 | PROTECTION POLICY 6,

|

| OTHER (SPECIFY).. 97,

Q71. Now thinking about your youngest |

child on the day before, can you tell | CAN'T SAY........ 98,

me if he or she was out of doors for |

longer than 15 minutes between 11am | +------------------------------------+

and 3pm on Saturday? | | IF YOUNGEST CHILD REGULARLY |

| | ATTENDS CRECHE, CHILD-CARE CENTRE |

YES.............. 1 | | OR KINDERGARTEN (CODE 1 ON Q72) |

| | ASK: |

NO............... 2 | +------------------------------------+

|

CAN'T SAY........ 3 | Q75. Can you tell me whether at the

| centre they have provided a playground

| with lots of shade?

IF YOUNGEST CHILD IS UNDER 5 YEARS OF |

AGE (CODES 1 TO 5 ON Q67) ASK: | YES.............. 1

|

| NO............... 2

Q72. Thinking about your youngest |

child, does he or she regularly attend | PLAY INDOORS ONLY 3

a creche, child-care centre or |

kindergarten? | CAN'T SAY........ 4

|

YES.............. 1 | Q76. Can you tell me whether at the

| centre the children are asked to wear

NO............... 2 | hats when they play outside?

|

CAN'T SAY........ 3 | YES.............. 1

|

| NO............... 2

IF YOUNGEST CHILD REGULARLY ATTENDS |

CRECHE, CHILD-CARE CENTRE OR | PLAY INDOORS ONLY 3

KINDERGARTEN (CODE 1 ON Q72) ASK: |

| CAN'T SAY........ 4

|

Q73. In your opinion, would you say | +------------------------------------+

that the centre your child attends has | | IF PARENT OR GUARDIAN OF CHILDREN |

made an effort to protect the children | | UNDER 16 (CODE 1 ON Q66) ASK: |

from sunburn when they play outside? | +------------------------------------+

|

YES.............. 1 | Q77. How many children under 16 years

| of age do you have currently attending

NO............... 2 | secondary school?

|

PLAY INDOORS ONLY 3 | ONE.............. 1

|

CAN'T SAY........ 4 | TWO OR MORE...... 2

|

| NONE............. 3

IF THINKS CENTRE MADE AN EFFORT (CODE |

1 ON Q73) ASK: | CAN'T SAY........ 4

SUN PROTECTION SURVEY 2000/2001

(C) Roy Morgan Research Pty Ltd. 2000.

DATE 29-NOV-00 SUN PROTECTION SURVEY 2000/2001 PAGE 18

________________________________________________________________________________

IF HAVE ONE CHILD UNDER 16 ATTENDING | IF HAVE ONE CHILD UNDER 16 ATTENDING

SECONDARY SCHOOL (CODE 1 ON Q77) ASK: | PRIMARY SCHOOL (CODE 1 ON Q80) ASK:

|

Q78A. In your opinion, would you say |

that the secondary school your child | Q81A. In your opinion, would you say

attends has made an effort to protect | that the primary school your child

the children from sunburn when they | attends has made an effort to protect

are outside during breaks or sport? | the children from sunburn when they

| are outside during breaks or sport?

YES.............. 1 |

| YES.............. 1

NO............... 2 |

| NO............... 2

CAN'T SAY........ 3 |

| CAN'T SAY........ 3

IF HAVE TWO OR MORE CHILDREN UNDER 16 |

ATTENDING SECONDARY SCHOOL (CODE 2 ON |

Q77) ASK: | IF HAVE TWO OR MORE CHILDREN UNDER 16

| ATTENDING PRIMARY SCHOOL (CODE 2 ON

Q78B. In your opinion, would you say | Q80) ASK:

that the secondary school THE YOUNGEST |

OF THESE CHILDREN attends has made an |

effort to protect the children from | Q81B. In your opinion, would you say

sunburn when they are outside during | that the primary school THE YOUNGEST

breaks or sport? | OF THESE CHILDREN attends has made an

| effort to protect the children from

YES.............. 1 | sunburn when they are outside during

| breaks or sport?

NO............... 2 |

| YES.............. 1

CAN'T SAY........ 3 |

| NO............... 2

IF THINKS SECONDARY SCHOOL HAS MADE AN |

EFFORT (CODE 1 ON Q78A OR Q78B) ASK: | CAN'T SAY........ 3

|

Q79. Can you tell me the type of |

things they do to protect the children | IF THINKS PRIMARY SCHOOL HAS MADE AN

from the sun? | EFFORT (CODE 1 ON Q81A OR Q81B) ASK:

HIGHLIGHT ALL MENTIONED. |

|

IF OTHER, HIGHLIGHT OTHER AND TYPE IN | Q82. Can you tell me the type of

RESPONSE | things they do to protect the children

| from the sun?

PROVIDE SHADE | HIGHLIGHT ALL MENTIONED.

OUTDOORS......... 1, |

| IF OTHER, HIGHLIGHT OTHER AND TYPE IN

PROVIDE SUNSCREEN 2, | RESPONSE

|

HAT-WEARING...... 3, | PROVIDE SHADE

| OUTDOORS......... 1,

LIMIT OUTDOOR |

ACTIVITIES | PROVIDE SUNSCREEN 2,

BETWEEN 11AM AND |

3PM.............. 4, | HAT-WEARING...... 3,

|

PROTECTIVE SPORTS | LIMIT OUTDOOR

UNIFORM.......... 5, | ACTIVITIES

| BETWEEN 11AM AND

HAVE A SUN | 3PM.............. 4,

PROTECTION POLICY 6, |

| PROTECTIVE SPORTS

OTHER (SPECIFY).. 97, | UNIFORM.......... 5,

|

CAN'T SAY........ 98, | HAVE A SUN

| PROTECTION POLICY 6,

+------------------------------------+ |

| IF PARENT OR GUARDIAN OF CHILDREN | | OTHER (SPECIFY).. 97,

| UNDER 16 (CODE 1 ON Q66) ASK: | |

+------------------------------------+ | CAN'T SAY........ 98,

|

Q80. How many children under 16 years |

of age do you have currently attending | +------------------------------------+

primary school? | | ASK EVERYONE. |

| +------------------------------------+

ONE.............. 1 |

| +------------------------------------+

TWO OR MORE...... 2 | | Just so we can check that we've |

| | interviewed a good sample of |

NONE............. 3 | | people, I'd like to ask you a few |

| | questions about yourself. |

CAN'T SAY........ 4 | +------------------------------------+

SUN PROTECTION SURVEY 2000/2001

(C) Roy Morgan Research Pty Ltd. 2000.

DATE 29-NOV-00 SUN PROTECTION SURVEY 2000/2001 PAGE 19

________________________________________________________________________________

Q53D.Are you now in paid employment? | Q56C. Could you please tell me your

| highest level of education?

IF YES ASK: Is that full-time for 35 |

hours or more a week, or part-time? | SOME PRIMARY

| SCHOOL........... 1

IF NO ASK: Are you retired, studying |

or home duties? | FINISHED PRIMARY

| SCHOOL........... 2

YES - FULL-TIME.. 1 |

| SOME SECONDARY

YES - PART-TIME.. 2 | SCHOOL........... 3

|

NO - HOME | SOME TECHNICAL OR

DUTIES/DON'T WORK 3 | COMMERCIAL....... 4

|

NO - AT | 4TH FORM/INTERME-

SCHOOL/STUDYING | DIATE, 5TH

FULL TIME........ 4 | FORM/LEAVING..... 5

|

NO - RETIRED..... 5 | TECHNICAL

| SCHOOL/TAFE...... 6

IF RESPONDENT WORKS (CODE 1 OR 2 ON |

Q53D), ASK | VCE/HSC OR MATRIC 7

|

Q53E.When you're working at your job, | SOME UNI/C.A.E... 8

about how much time do you spend |

outside? | TERTIARY DIPLOMA. 9

|

READ OUT... | CURRENTLY

| UNIVERSITY OR

All.............. 1 | C.A.E............ 10

|

Most............. 2 | TERTIARY DEGREE.. 11

|

Half............. 3 |

| Q57. May I have your name and address

Very little or... 4 | so we can contact you again if we need

| any further information?

(DO NOT READ) |

NONE............. 5 | IF YES, ENTER NAME ON FIRST LINE AND

| ADDRESS ON THE LINES FOLLOWING

Q54. What's the main income earner's |

(last) occupation -the position and | |_|_|_|_|_|_|_|_|_+

industry? |

1: Professional | |_|_|_|_|_|_|_|_|_+

2: Owner or Executive |

3: Owner of Small Businesses | |_|_|_|_|_|_|_|_|_+

1 |

1: Sales | |_|_|_|_|_|_|_|_|_+

1 |

2: Semi-Professional |

4: Other White Collar | +------------------------------------+

5: Skilled | | Thank you for your time and |

6: Semi-Skilled | | assistance for the interview. |

7: Unskilled | +------------------------------------+

8: Farm Owner |

9: Farm Worker 10: | Q. RECORD YOUR NAME FOR A TRUE AND

No Occupation | HONEST INTERVIEW.

|

|__|__+ | |_|_|_|_|_|_|_|_|_+

SUN PROTECTION SURVEY 2000/2001

(C) Roy Morgan Research Pty Ltd. 2000.
