# Supplementary material for: Implementation of the SunSmart program and population sun protection behaviour in Melbourne, Australia: Results from cross-sectional summer surveys from 1987 to 2017
Source: PLoS Med. 2019 Oct 8;16(10):e1002932. doi: 10.1371/journal.pmed.1002932 (PMC6782093; doi:10.1371/journal.pmed.1002932)
Supplement: S2 Appendix — (DOCX) [file pmed.1002932.s002.docx]

**S2 Appendix:** Sun Protection Survey questions (2016-17)

**SURVEY QUESTIONS**

*Programming notes are in [italicized red text].*

*Interviewer instructions are in blue text*

**RECRUITMENT**

*[Select single response]*

QP. Good [Afternoon/ Evening]. My name is…. . I'm from Roy Morgan Research, the people who conduct the Morgan Gallup Poll. Today we're doing a survey about people's attitudes towards being out in the sun, and we'd like the opinion of people aged 12 to 69.

May I speak to the youngest male at home aged 12 years or over?

If Mobile sample ask “Is now a good time to talk?”

IF NO MALES ASK: Then may I speak to the youngest female at home aged 12 years or over?

SELECT IF QUOTAS ARE STILL OPEN:

#/ MALES 12-13/ /#/ FEMALES 12-13//

#/ MALES 12-17/ /#/ FEMALES 12-17//

#/ MALES 18-34/ /#/ FEMALES 18-34//

#/ MALES 35-69/ /#/ FEMALES 35-69//

IF NO-ONE AT HOME AGED 12-69, RECORD INELIGIBLE HOUSEHOLD BELOW

1 ELIGIBLE 12-69 YEARS

2 INELIGIBLE HOUSEHOLD

IF INELIGIBLE HOUSEHOLD , **SAY**

Thank you for your time and assistance

ENDIF

**RESPONDENT DEMOGRAPHICS**

**(Also used for recruitment quotas)**

*[Single response]*

QA. RECORD SEX OF RESPONDENT (**DO NOT ASK**!)

1 MALE

2 FEMALE

*[Quantity]*

AGE1. To make sure we have a true cross-section of people, would you mind telling me your age please?

|  |  |  |
| --- | --- | --- |

IF CAN'T SAY, Abandon respondent and **READ OUT** Thank you for your help.

IF AGED LESS THAN SIXTEEN **OBTAIN GUARDIAN PERMISSION**

*[Single response]*

QPER. We would like to check that your parents would not mind you helping us out with this survey about people's attitudes towards being out in the sun. Can you please check that this is ok?

1 YES - GUARDIAN PERMISSION OBTAINED

2 NO GUARDIAN AVAILABLE TO CHECK WITH - MAKE AN APPOINTMENT

3 NO - GUARDIAN REFUSED PERMISSION TO CONTINUE

IF PARENT REFUSED PERMISSION – **Abandon respondent** and

**READ OUT** Thank you for your help.

*[END IF]*

**RESPONDENT QUOTA CHECKS**

***[ASK EVERYONE]***

*[Single response]*

QUOTA CHECK [THESE SHOULD MATCH QUOTAS NEEDED]

1 MALE 12-13 - SEE NEXT QUOTA CHECK

2 MALE 12-17

3 MALE 18-34

4 MALE 35-69

5 FEMALE 12-13 - SEE NEXT QUOTA CHECK

6 FEMALE 12-17

7 FEMALE 18-34

8 FEMALE 35-69

IF QUOTA FULL **SAY**:

I'm sorry #/sir/madam/ but we have already interviewed enough #/males/females/ of your age group for our survey.

*ENDIF*

*[Single response]*

QUOTA CHECK FOR 12-13 YRS ONLY

1 MALE 12-13

5 FEMALE 12-13

IF QUOTA FULL **SAY**: I'm sorry #/sir/madam/ but we have already interviewed enough #/males/females/ of your age group for our survey.

*ENDIF*

*[Quantity]*

QB. May I have your postcode please?

|  |  |  |  |
| --- | --- | --- | --- |

IF RESPONDENT DOESN'T KNOW, **ASK**: Well, could you please tell me the suburb in which you live?

TYPE IN SUBURB, LOOK UP AND FILL IN POSTCODE BEFORE COMPLETION OF INTERVIEW.

[suburb name]

It looks like we have a sufficient number of people who meet similar criteria to yourself, and because of this you will not be required to take part in this survey. We apologise for the inconvenience.

Thank you for your time and assistance. This market research is carried out in compliance with the Privacy Act, and the information you provided will be used only for research purposes.

If you would like any more information about this project or Roy Morgan Research, you can phone us on 1800 337 332**LANDLINE AND MOBILE PHONE USE**

*(Ask Mobile Phone sample only)*

QPHONE1. Do you live in a home that also has a landline telephone?

1 YES

2 NO

*ENDIF*

*(Ask Telephone Landline sample only)*

QPHONE2. Do you personally have a mobile phone?

1 YES

2 NO

*ENDIF*

***[ASK EVERYONE]***

*[Quantity]*

QPHONE3. How many people, including yourself, usually live in your household?

|  |  |  |
| --- | --- | --- |

IF CAN'T SAY, Abandon respondent and

**READ OUT** Thank you for your help.

Unfortunately that’s all the questions we have for you today as your responses don’t match the type of people we are looking for in this particular survey.

Thank you for your time and assistance. This market research is carried out in compliance with the Privacy Act, and the information you provided will be used only for research purposes.

If you would like any more information about this project or Roy Morgan Research, you can phone us on 1800 337 332 **SKIN TYPE – SUNBURN SENSITVITY**

*[Single response]*

Q1. Now, I'd like to ask a few questions about your skin type.

Suppose your skin was exposed to strong sunshine at the beginning of summer with no protection at all. If you stayed in the sun for 30 minutes, would your skin:

**(READ OUT)**

1 JUST BURN AND NOT TAN AFTERWARDS

2 BURN FIRST, THEN TAN AFTERWARDS, OR

3 NOT BURN AT ALL, JUST TAN

4 (DO NOT READ) NOTHING WOULD HAPPEN

5 (DO NOT READ) CAN'T SAY

IF SAYS 'GO RED' INTERPRET AS A BURN AND **ASK**: Would you then tan afterwards or not?

**SKIN TYPE – SKIN COLOUR**

*[Single response]*

Q2. How would you describe your skin colour when you don't have any tan?

IF RESPONDENT SAYS 'FAIR' OR 'DARK' **ASK**: Would that be very fair/dark or fair/dark?

IF RESPONDENT HESITATES, PROMPT WITH:

When you don't have any tan would you say your skin colour is...

**(READ OUT)**

1 VERY FAIR

2 FAIR

3 MEDIUM

4 OLIVE

5 DARK

6 VERY DARK

7 BLACK

8 **(DO NOT READ)** DON'T KNOW/ CAN'T SAY

**TAN BEHAVIOUR**

*[Single response]*

Q3A. Have you made any attempt to get a suntan this season?

1 YES

2 NO

**TAN PREFERENCE**

*[Single response]*

Q3B. Do you like to get a suntan or not?

1 YES

2 NO

*IF LIKES TO GET A SUNTAN (CODE 1 ON Q3B),* ***ASK****:*

*[Single response]*

Q4. How DEEP a tan do you like to get?

IF RESPONDENT SAYS "GOLDEN", **SAY**: Does that mean light or moderate?

IF RESPONDENT HESITATES PROMPT WITH:

Do you like that tan to be..

**(READ OUT)**

1 LIGHT

2 MODERATE

3 DARK

4 VERY DARK

5 (**DO NOT READ**) CAN'T SAY

**ENDIF**

**SUNBURN**

**(READ OUT)** The next questions are about sunburn. By sunburn we mean any amount of reddening of the skin after being in the sun.

*[Multiple response]*

*[Programming note: If interviewed on a Monday evening program “yesterday” and “Saturday”. If interviewed on a Tuesday or Wednesday evening program “Sunday? What about Saturday”. The exception of referring to days on the weekend would occur only after a public holiday on a Monday/Tuesday, which does not apply in 2016-17]*

Q4A. Did you get at all sunburnt yesterday? What about on Saturday?

1… SUNDAY

2… SATURDAY

3… *[Single]* NEITHER DAY

*[Data Specification Note: the CATI schedule differs from the final data codes.*

*The Q4A output should be recorded as three sunburn variables:*

*‘burnsun’, ‘burnsat’; and, ‘NOTBURNT’ CODES 1 Yes, 2 No]*

*IF SUNBURNT (CODES 1 - 2 ON Q4A),* ***ASK:***

**BODY PARTS BURNT**

*[Multiple]*

Q4B. Which part or parts of you got sunburnt at the weekend? Where else?

1 FACE

2 NOSE

3 HEAD

4 EARS

5 CHEST

6 STOMACH

7 BACK

8 NECK

9 SHOULDERS

10 ARMS

11 HANDS

12 LEGS

13 BACK OF KNEES

14 FEET

**WORST BURN**

*[Single response]*

Q4C. Which part was burnt WORST?

1 FACE

2 NOSE

3 HEAD

4 EARS

5 CHEST

6 STOMACH

7 BACK

8 NECK

9 SHOULDERS

10 ARMS

11 HANDS

12 LEGS

13 BACK OF KNEES

14 FEET

**SEVERITY**

*[Single response]*

Q5AI. Which of the following statements BEST describes the burn on your **[body part burnt - programmed answer to Q4C]**?

**(READ OUT)**

1 RED WITHOUT BEING TENDER

2 RED AND TENDER

3 RED, TENDER AND BLISTERED

IF BURN WAS RED WITHOUT BEING TENDER (CODE 1 ON Q5AI), **ASK**:

*[Single response]*

Q5AII. Was the redness present the next morning?

1 YES

2 NO

3 COULDN'T DECIDE

4 DON'T KNOW (DIDN'T LOOK)

**ENDIF**

IF BLISTERED (CODE 3 ON Q5AI), **ASK**:

*[Single response]*

Q5B. Are the blisters weeping?

1 YES

2 NO

**ENDIF**

***ENDIF***

*PROGRAMMING NOTE FOR OUTDOOR ACTIVTY QUESTIONS*

*For all questions referring to* ***#/11am and 3pm/10am and 2pm/***

*If respondent is resident in a state/territory with* ***Daylight Saving*** *on interview week,*

*then program* ***“11am and 3pm”***

*If respondent is resident in a state/territory with* ***Standard Time*** *on interview week,*

*then program* ***“10am and 2pm” at #position***

**OUTDOOR ACTIVITY - SUNDAY**

Next we'd like to ask about your outdoor activities over the weekend between #/11am and 3pm/10am and 2pm/ because that's when the sun shines strongest.

*[Single response]*

Q6B. Thinking back to Sunday. Were you out of doors for longer than 15 minutes between #/11am and 3pm/10am and 2pm/ ? By out of doors we mean not in a building and not in a covered vehicle.

IF INTERMITTENTLY OUTDOORS ASK: Would you say you were actually out of doors for longer than 15 minutes in total?

1 YES

2 NO *[IF Code 2 skip to Q6A]*

**IF OUTDOORS FOR LONGER THAN 15 MINUTES (CODE 1 ON Q6B) ASK:**

*[Single response]*

Q7. What activity were you DOING MOSTLY during that time out of doors?

TYPE IN RESPONSE VERBATIM FOR PROGRAMMING [This will be used in more questions about this activity]

________________________________________________

*[Programmer note –The above text [verbatim response Q7] needs to be programmed for use in later questions. The activity category values (numbers) listed below are to be coded at a later stage]*

ACTIVITY RESPONSE IS CODED AS BELOW

1 NoScreen BEACH

2 NoScreen CRICKET AT BEACH

3 NoScreen WATER SKIING

4 NoScreen SURFING/ SAIL BOARDING/ WIND SURFING

5 NoScreen SAILING

6 NoScreen BOATING

7 NoScreen SCUBA DIVING

8 NoScreen WALKING ON BEACH

9 NoScreen SITTING AT BEACH

10 NoScreen RELAXING ON BEACH

11 NoScreen BBQ AT BEACH

12 NoScreen PICNIC AT BEACH

13 NoScreen CAMPING/ FISHING AT BEACH

14 NoScreen AT POOL

15 NoScreen AT POOL IN SUN

16 NoScreen SWIMMING IN BACKYARD POOL

17 NoScreen SUNBATHING AT POOL

18 NoScreen SITTING AT POOL

19 NoScreen RELAXING AT POOL

20 NoScreen SOCIALISING AT POOL

21 NoScreen IN GARDEN/ PARK

22 NoScreen IN SHADE

23 NoScreen SUNBATHING

24 NoScreen SITTING

25 NoScreen SITTING IN GARDEN/ PARK

26 NoScreen SITTING IN SUN

27 NoScreen SITTING IN SHADE

28 NoScreen RELAXING

29 NoScreen RELAXING IN GARDEN/ PARK

30 NoScreen RELAXING IN SUN

31 NoScreen RELAXING IN GARDEN IN SUN

32 NoScreen RELAXING IN SHADE

33 NoScreen BBQ

34 NoScreen PICNIC

35 NoScreen SOCIALISING/ AT A FUNCTION - WEDDING, CHURCH

36 NoScreen DRINKING

37 NoScreen DRINKING IN GARDEN

38 NoScreen WATCHING SPORT

39 NoScreen COACHING/ SUPERVISING SPORT IN GARDEN

40 NoScreen SIGHTSEEING/ OUTINGS/ AT ZOO/ MINI GOLF

41 NoScreen DRIVING/ STOPS DURING TRAVEL/ IN CAR/ LOOKING AT HOUSES

42 NoScreen DRIVING WITH SUNROOF

43 NoScreen WAITING/ STANDING

44 NoScreen CAMPING/ FISHING/ YABBYING

45 NoScreen POTTERY/ READING/ DRAWING

46 NoScreen EATING/ LUNCHING AT A CAFE

47 NoScreen EATING/ LUNCHING IN GARDEN

48 NoScreen AT A PARTY/ PARTYING

49 NoScreen SHOPPING AT A MARKET/ GARAGE SALE/ OUTDOORS

50 NoScreen FESTIVAL/ CARNIVAL/ EXPO/ SHOW/ AMUSEMENT PARK

51 NoScreen TENNIS

52 NoScreen CRICKET

53 NoScreen GOLF

54 NoScreen NETBALL

55 NoScreen SOCCER

56 NoScreen BOWLS

57 NoScreen VOLLEYBALL

58 NoScreen FOOTBALL

59 NoScreen BASEBALL

60 NoScreen TABLE TENNIS

61 NoScreen SOFTBALL

62 NoScreen SPORT (OTHER)

63 NoScreen COACHING/ SUPERVISING SPORT

64 NoScreen BASKETBALL

65 NoScreen WALKING

66 NoScreen WALKING IN GARDEN

67 NoScreen BUSHWALKING

68 NoScreen SKATEBOARDING/ SKATING/ ROLLERBLADING

69 NoScreen EXERCISING

70 NoScreen BIKE RIDING

71 NoScreen HORSE RIDING

72 NoScreen KICKING THE FOOTY

73 NoScreen CLIMBING TREES

74 NoScreen RUNNING

75 NoScreen RACING PIGEONS

76 NoScreen TARGET SHOOTING/ SHOOTING/ ARCHERY

77 NoScreen FLYING A KITE

78 NoScreen JOGGING

79 NoScreen BADMINTON

80 NoScreen TRAMPOLINING

81 NoScreen DOG - WALKING/ WASHING/ PLAYING WITH

82 NoScreen TOBOGGAN RIDES

83 NoScreen PLAYING - WITH FRIENDS, KIDS

84 NoScreen MOTOR RACING/ MOTOR BIKE RIDING/ GO-CART RACING

85 NoScreen WORKING ON THE HOUSE

86 NoScreen WORKING AROUND THE HOUSE

87 NoScreen WORKING ON THE POOL

88 NoScreen WORKING ON THE CAR/ BOAT/ CARAVAN

89 NoScreen OTHER WORK - AT HOME

90 NoScreen WASHING/ HANGING WASHING

91 NoScreen PACKING/ UNPACKING/ SHIFTING/ MOVING

92 NoScreen SHOPPING

93 NoScreen FARM JOBS

94 NoScreen BUILDING RELATED JOBS

95 NoScreen GARDEN RELATED JOBS

96 NoScreen OTHER JOBS

97 NoScreen UNSPECIFIED PAID JOBS

98 NoScreen WORKING

99 NoScreen WASHING/ CLEANING CAR

100 NoScreen GARDENING

101 NoScreen LAWN MOWING

102 NoScreen WATERING

103 NoScreen SWIMMING

104 NoScreen OUTSIDE (UNSPEC)

997 Openend OTHER (SPECIFY)

998 NoScreen Single CAN'T SAY

IF DID NOT SAY WHERE DOING OUTDOOR ACTIVITY ON SUNDAY (NOT CODE 998 ON Q7), **ASK:**

*[Single response]*

Q13. Where were you when you were [**verbatim response - activity outdoors Q7**]?

INTERVIEWER NOTE: **READ OUT IF NECESSARY**

IF OTHER, SELECT OTHER AND TYPE IN RESPONSE

IF LOCATION ALREADY STATED BY RESPONDENT IN PREVIOUS QUESTION, JUST CODE BELOW WITHOUT RER-ASKING THIS QUESTION.

1 AT THE BEACH/LAKE/RIVER

2 AT A LOCAL POOL

3 AT A BACKYARD POOL

4 AT A PUBLIC PARK/GARDEN

5 AT A PUBLIC PLAYGROUND

6 AT SCHOOL GROUNDS

7 AT A SPORTS CENTRE/GROUNDS

8 AT AN OUTDOOR CAFE/RESTAURANT/PUB

9 AT A MARKET/FAIR/OUTDOOR SHOPPING CENTRE

10 ON THE STREET/FOOTPATH/WALKWAY

11 AT HOME/FRIEND'S PLACE

12 AT HOLIDAY HOUSE/CAMPING GROUND/HOTEL/MOTEL

13 ON A FARM

14 IN THE BUSH

15 AT A RACE TRACK/MOTOCROSS TRACK

16 AT WORKPLACE

17 DRIVING/ TRAVELLING/ TOURING/ ON THE ROAD

997 OTHER (SPECIFY)

998 CAN'T SAY

***ENDIF***

**AMOUNT OF TIME OUTDOORS PEAK UV PERIOD - SUNDAY**

*[Quantity] {Min: 0, Max: 240; Can’t say value 241}*

Q8. About how much time in total did you spend out of doors on Sunday between #/11am and 3pm/10am and 2pm/ [**verbatim response - activity outdoors Q7**] in total?

TYPE IN THE TOTAL IN MINUTES.

|  |  |  |
| --- | --- | --- |

IF RESPONDENT GAVE ANSWER IN HOURS, CONVERT TO MINUTES.

IF CAN'T SAY TYPE VALUE 241

1.5 HOURS = 90 MINUTES

2 HOURS = 120 MINUTES

2.5 HOURS = 150 MINUTES

3 HOURS = 180 MINUTES

3.5 HOURS = 210 MINUTES

4 HOURS = 240 MINUTES

IF Q8 ANSWER IS 0 MINUTES, SAY:

Earlier you mentioned that you were outdoors between #/11am and 3pm/10am and 2pm/ for longer than 15 minutes and that the activity you were mostly doing was [**verbatim response - activity outdoors Q7**]. About how much time in total did you spend out of doors on Sunday between #/11am and 3pm/10am and 2pm/ [**verbatim response - activity outdoors Q7**].

INTERVIEWER NOTE: IF RESPONDENT HAD INCORRECTLY ANSWERED CURRENT QUESTION, GO BACK ONE SCREEN AND CHANGE ANSWER AT CURRENT QUESTION.
 IF RESPONDENT INSISTS THAT ANSWER OF LESS THAN 15 MINUTES (INCLUDING ZERO) AT CURRENT QUESTION IS CORRECT, GO BACK AND RE-CONFIRM EARLIER QUESTIONS ABOUT BEING IN SUN FOR MORE THAN 15 MINUTES DURING PEAK SUNLIGHT PERIOD, AND THE MAIN ACTIVITY UNDERTAKEN DURING THAT PERIOD.

ENDIF

**LOCATION OUTDOOR ACTIVITY - SUNDAY**

*[Q9A-Q9H single response – filter Q by state/territory (residential postcode)]*

IF Vic POSTCODE **ASK**:

Q9A. Were you in the **Melbourne** metropolitan area when you were [**verbatim response - activity outdoors Q7**]?

1 YES

2 NO (GO TO Q15B)

*ENDIF*

IF NSW POSTCODE **ASK**:

Q9B. Were you in the **Sydney** metropolitan area when you were [**verbatim response - activity outdoors Q7**]?

1 YES

2 NO (GO TO Q15B)

*ENDIF*

IF Qld POSTCODE **ASK**:

Q9C. Were you in the **Brisbane** metropolitan area when you were [**verbatim response - activity outdoors Q7**]?

1 YES

2 NO (GO TO Q15B)

*ENDIF*

IF WA POSTCODE **ASK**:

Q9D. Were you in the **Perth** metropolitan area when you were [**verbatim response - activity outdoors Q7**]?

1 YES

2 NO (GO TO Q15B)

*ENDIF*

IF SA POSTCODE **ASK**:

Q9E. Were you in the **Adelaide** metropolitan area when you were [**verbatim response - activity outdoors Q7**]?

1 YES

2 NO (GO TO Q15B)

*ENDIF*

IF Tas POSTCODE **ASK**:

Q9F. Were you in the **Hobart** metropolitan area when you were [**verbatim response - activity outdoors Q7**]?

1 YES

2 NO (GO TO Q15B)

*ENDIF*

IF ACT POSTCODE **ASK**:

Q9G. Were you in the **Canberra** metropolitan area when you were [**verbatim response - activity outdoors Q7**]?

1 YES

2 NO (GO TO Q15B)

ENDIF

IF NT POSTCODE **ASK**:

Q9H. Were you in the **Darwin** metropolitan area when you were [**verbatim response - activity outdoors Q7**]?

1 YES

2 NO (GO TO Q15B)

*ENDIF*

*IF NOT IN THE METROPOLITAN AREA (CODE 2 Q9 A-H) for Sunday activity between #/11am and 3pm/10am and 2pm,* ***ASK:***

*[Single response]*

Q15B. What was the nearest suburb or town when you were [**verbatim response - activity outdoors Q7**]

IF OTHER, SELECT OTHER AND TYPE IN RESPONSE

997 OTHER (SPECIFY)

998 **(DO NOT READ OUT)** CAN'T SAY

**ENDIF**

**SUN PROTECTION DURING OUTDOOR ACTIVITY - SUNDAY**

**SHADE**

*[Single response]*

Q10B.Were you MOSTLY IN THE SHADE or MOSTLY OUT IN THE OPEN while you were [**verbatim response - activity outdoors Q7**]?

1 IN THE SHADE

2 IN THE OPEN

3 IN SHADE AND OUT IN OPEN EQUALLY

4 CAN'T SAY

**CLOTHING**

*[Single response]*

Q11A1.Now we need to ask you some questions in detail about what you were wearing yesterday (SUNDAY) to find out how much your skin was exposed to direct sunlight.

Would you mind telling me what you were wearing while you were [**verbatim response - activity outdoors Q7**]?

IF RESPONDENT HESITATES, **SAY**:

Can you remember what you were wearing on the top part of your body?

RETURN FOR CLOTHING WORN ON LOWER PART OF BODY

1 TOP/DRESS/WETSUIT

2 SWIMWEAR

3 TOPLESS

*[Single response]*

Q11A2. CLOTHING WORN ON LOWER PART OF BODY

RECORD OR IF RESPONDENT HESITATES PROMPT WITH:

Can you remember what you were wearing on the lower part of your body?

1 TROUSERS/JEANS/SHORTS/SKIRT/DRESS/WETSUIT

2 SWIMWEAR

3 BOTTOMLESS

IF TOP/DRESS/WETSUIT (CODE 1 ON Q11A1), **ASK**

*[Single response]*

Q11B.How long were the sleeves of your top/dress/wetsuit?

IF RESPONDENT HESITATES, **READ OUT**:

1 WRIST LENGTH

2 3/4 LENGTH

3 ELBOW LENGTH

4 SHORT

5 SLEEVELESS

ENDIF

IF TROUSERS/JEANS/SHORTS/SKIRT/DRESS/WETSUIT (CODE 1 ON Q11A2), **ASK:**

*[Single response]*

Q11C.How long were/was your trousers/jeans/shorts/skirt/dress/ wetsuit?

IF RESPONDENT HESITATES, **READ OUT**:

1 ANKLE LENGTH

2 3/4 LENGTH

3 KNEE LENGTH

4 MINI SKIRT/SHORT SHORTS

ENDIF

IF SWIMWEAR (CODE 2 ON Q11A1 OR CODE 2 ON Q11A2), **ASK**:

*[Multiple]*

Q11D.What sort of swimwear/bathers were you wearing?

1 ONE PIECE BATHERS

2 TWO PIECE/BIKINI

3 BIKINI TOP ONLY

4 BIKINI BOTTOM ONLY

5 LONG/BOARD SHORTS

6 SHORT SHORTS

7 BRIEFS/SPEEDOS

8 RASH VEST

ENDIF

**HEADWEAR**

*[Single response]*

Q11G.Were you wearing a cap, hat or sun visor?

1 HAT

2 CAP

3 VISOR

4 NONE WORN

IF HAT OR CAP WORN (CODES 1 OR 2 ON Q11G), **ASK**:

*[Single response]*

Q11H.Did your #/hat/cap/ have a wide brim or a narrow brim?

1 WIDE BRIM

2 NARROW BRIM

3 NO BRIM

ENDIF

IF HAT, CAP OR VISOR WORN (CODES 1 OR 2 OR 3 ON Q11G), **ASK**:

*[Single response]*

Q11H2. Did it have a flap which covered the back of your neck?

1 YES

2 NO

ENDIF

**SUNGLASSES**

*[Single response]*

Q11I.Were you wearing any sunglasses?

1 YES

2 NO

**SUNSCREEN**

*[Single response]*

Q24. Now some questions about sunscreen.

A sunscreen is a gel, lotion or cream that filters out ultraviolet sunlight to prevent burning and other skin damage.

Did you use a sunscreen between #/11am and 3pm/10am and 2pm/ on Sunday?

1 YES, USED SUNSCREEN

2 NO, DIDN'T USE SUNSCREEN

3 MAKEUP WITH A SUNSCREEN ONLY

IF USED SUNSCREEN (CODE 1 OR 3 ON Q24) **ASK**:

*[Single response]*

Q25A.Did you apply the sunscreen: (READ OUT)

IF RESPONDENT ANSWERS "as soon as I went out in the sun" ENTER AS '2'

IF RESPONDENT ANSWERS "after slightly sun burnt" ENTER AS '3'

1 BEFORE GOING OUT IN THE SUN

2 AFTER YOU'D BEEN IN THE SUN A WHILE

3 **(DO NOT READ OUT)** AFTER SLIGHTLY SUN BURNT

*[Quantity] {Min: 0, Max: 51; Can’t say value 99}*

Q26B.What was the sun protection factor of the sunscreen you used?

|  |  |  |
| --- | --- | --- |

IF RESPONDENT SAYS "15"+ ENTER "16".

IF RESPONDENT SAYS "30"+ ENTER "31".

IF RESPONDENT SAYS "50"+ ENTER "51".

IF "DON'T KNOW" Type 99.

*[Single response]*

Q27. On what parts of the body did you apply sunscreen?

Where else?

Anywhere else?

PROMPT: Did you apply sunscreen to your...

**READ OUT LIST** EXCLUDING ANSWERS ALREADY GIVEN

1 FACE

2 NOSE

3 **(DO NOT READ)** HEAD

4 **(DO NOT READ)** EARS

5 CHEST

6 STOMACH

7 BACK

8 NECK

9 SHOULDERS

10 ARMS

11 HANDS

12 LEGS

13 BACK OF KNEES

14 FEET

ENDIF

**EXPOSED SKIN**

*[Multiple]*

Q28. Were there any areas of your body exposed to the sun that didn't have sunscreen, clothing or a hat covering them?

**READ OUT IF HESITATES**

1 FACE

2 NOSE

3 HEAD

4 EARS

5 CHEST

6 STOMACH

7 BACK

8 NECK

9 SHOULDERS

10 ARMS

11 HANDS

12 LEGS

13 BACK OF KNEES

14 FEET

15 NONE

IF ENGAGED IN ACTIVITIES ON SUNDAY AND MOSTLY OUT IN OPEN (CODE 1 TO 997 Q7, CODE 2 Q10B), **ASK:**

**SHADE AVAILABLE OUTDOOR ACTIVITY - SUNDAY**

*[Single response]*

Q25. Earlier, you said that you were mostly out in the open when you were [**verbatim response - activity outdoors Q7**] outdoors on Sunday between #/11am and 3pm/10am and 2pm/ . It may sound odd, but, would it have been possible for you to [**verbatim response - activity outdoors Q7**] mostly in the shade or indoors?

1 YES

2 NO

3 CAN'T SAY

IF NOT POSSIBLE (CODE 2 ON Q25), **ASK:**

*[Multiple] {Spread:20 }*

Q26A. Why was it not possible for you to [**verbatim response - activity outdoors Q7**] mostly in the shade or indoors?

Any other reason?

Any others?

IF OTHER, SELECT OTHER AND TYPE IN RESPONSE

1 NO SHADE AVAILABLE AT LOCATION

2 NO INDOOR FACILITY AVAILABLE

3 DIDN'T TAKE PORTABLE SHADE (E.G. UMBRELLA, PARASOL, SHADE CLOTH)

4 JUST IMPOSSIBLE TO DO IN THE SHADE

5 JUST IMPOSSIBLE TO DO INDOORS

6 I CHOSE TO BE IN THE SUN

97 OTHER (SPECIFY)

98 [Single] CAN'T SAY

ENDIF

IF ENGAGED IN ACTIVITIES ON SUNDAY AND MOSTLY IN SHADE (CODE 1 OR 3 ON Q10B AND Q7 IS 1-997), **ASK:**

*[Multiple] {Spread:20 }*

Q26D. Earlier, you said that you were **#/mostly in the shade// #/in the shade and out in the open equally**// **[Response Q 10B]** when you were [**verbatim response - activity outdoors Q7**] outdoors on Sunday between #/11am and 3pm/10am and 2pm/ . What type of shade from the sun did you use?

IF OTHER, SELECT OTHER AND TYPE IN RESPONSE

1 OUTDOOR SHADE STRUCTURES (E.G. VERANDAHS, GAZEBOS, SHADE SAILS, ETC.)

2 PORTABLE SHADE (E.G. UMBRELLAS, PARASOLS, SHADE SHELTERS, TENTS, ETC.)

3 TREES, HEDGES, ETC.

5 NoScreen SHADE FROM BUILDINGS/ HOUSES

97 OTHER (SPECIFY)

98 [Single] CAN'T SAY

ENDIF

**ENDIF**

***[ASK EVERYONE]***

**TOTAL TIME OUTDOORS – SUNDAY**

[Quantity] {Min: 0, Max: 841, Can’t say type value: 999}

Q6A. Thinking about the WHOLE day on Sunday - not just the time between #/11am and 3pm/10am and 2pm/ - about how much time did you spend out of doors? By out of doors we mean not in a building and not in a covered vehicle.

TYPE IN TOTAL **IN MINUTES** (IF RESPONDENT GAVE ANSWER IN HOURS, CONVERT TO MINUTES)

|  |  |  |
| --- | --- | --- |

1.5 HOURS = 90 MINUTES 2 HOURS = 120 MINUTES

2.5 HOURS = 150 MINUTES 3 HOURS = 180 MINUTES

3.5 HOURS = 210 MINUTES 4 HOURS = 240 MINUTES

4.5 HOURS = 270 MINUTES 5 HOURS = 300 MINUTES

5.5 HOURS = 330 MINUTES 6 HOURS = 360 MINUTES

6.5 HOURS = 390 MINUTES 7 HOURS = 420 MINUTES

7.5 HOURS = 450 MINUTES 8 HOURS = 480 MINUTES

8.5 HOURS = 510 MINUTES 9 HOURS = 540 MINUTES

If >14 hours type value 841

IF CAN'T SAY, **ASK**:

Well, could you estimate to the NEAREST 1/2 HOUR how much time you spent out of doors on Sunday?

IF STILL CAN’T SAY TYPE 999

|  |  |  |
| --- | --- | --- |

**INTERVIEWER CROSSCHECK CONSISTENCY WITH Q6B, Q8 now revised:**

Earlier you mentioned that you were outdoors on Sunday between #/11am and 3pm/10am and 2pm/ for longer than 15 minutes. Now if you were to think about the WHOLE day on Sunday, how much time did you spend out of doors?

INTERVIEWER NOTE: IF RESPONDENT HAD INCORRECTLY ANSWERED CURRENT QUESTION, GO BACK ONE SCREEN AND CHANGE ANSWER AT CURRENT QUESTION.

 IF RESPONDENT INSISTS THAT ANSWER OF LESS THAN 15 MINUTES AT CURRENT QUESTION IS CORRECT, MARK THE BOX BELOW.

|  |
| --- |

**OUTDOOR ACTIVITY - SATURDAY**

***[ASK EVERYONE]***

*[Single response]*

Q29B.Thinking back to Saturday. Were you out of doors for longer than 15 minutes between #/11am and 3pm/10am and 2pm/

IF INTERMITTENTLY OUTDOORS **ASK**:

Would you say you were actually out of doors for longer than 15 minutes in total?

1 YES

2 NO *[IF Code 2 skip to Q29A]*

IF OUTDOORS ON SATURDAY (CODE 1 ON Q29B) **ASK**:

*[Single response]*

Q30. What activity were you DOING MOSTLY during that time out of doors?

TYPE IN RESPONSE VERBATIM FOR PROGRAMMING

________________________________________________

**[Programmer note. The text response to Q30 will be programmed for use in later questions,. The activity category is coded after the interview]**

ACTIVITY RESPONSE IS CODED AS BELOW

1 NoScreen BEACH

2 NoScreen CRICKET AT BEACH

3 NoScreen WATER SKIING

4 NoScreen SURFING/ SAIL BOARDING/ WIND SURFING

5 NoScreen SAILING

6 NoScreen BOATING

7 NoScreen SCUBA DIVING

8 NoScreen WALKING ON BEACH

9 NoScreen SITTING AT BEACH

10 NoScreen RELAXING ON BEACH

11 NoScreen BBQ AT BEACH

12 NoScreen PICNIC AT BEACH

13 NoScreen CAMPING/ FISHING AT BEACH

14 NoScreen AT POOL

15 NoScreen AT POOL IN SUN

16 NoScreen SWIMMING IN BACKYARD POOL

17 NoScreen SUNBATHING AT POOL

18 NoScreen SITTING AT POOL

19 NoScreen RELAXING AT POOL

20 NoScreen SOCIALISING AT POOL

21 NoScreen IN GARDEN/ PARK

22 NoScreen IN SHADE

23 NoScreen SUNBATHING

24 NoScreen SITTING

25 NoScreen SITTING IN GARDEN/ PARK

26 NoScreen SITTING IN SUN

27 NoScreen SITTING IN SHADE

28 NoScreen RELAXING

29 NoScreen RELAXING IN GARDEN/ PARK

30 NoScreen RELAXING IN SUN

31 NoScreen RELAXING IN GARDEN IN SUN

32 NoScreen RELAXING IN SHADE

33 NoScreen BBQ

34 NoScreen PICNIC

35 NoScreen SOCIALISING/ AT A FUNCTION - WEDDING, CHURCH

36 NoScreen DRINKING

37 NoScreen DRINKING IN GARDEN

38 NoScreen WATCHING SPORT

39 NoScreen COACHING/ SUPERVISING SPORT IN GARDEN

40 NoScreen SIGHTSEEING/ OUTINGS/ AT ZOO/ MINI GOLF

41 NoScreen DRIVING/ STOPS DURING TRAVEL/ IN CAR/ LOOKING AT HOUSES

42 NoScreen DRIVING WITH SUNROOF

43 NoScreen WAITING/ STANDING

44 NoScreen CAMPING/ FISHING/ YABBYING

45 NoScreen POTTERY/ READING/ DRAWING

46 NoScreen EATING/ LUNCHING AT A CAFE

47 NoScreen EATING/ LUNCHING IN GARDEN

48 NoScreen AT A PARTY/ PARTYING

49 NoScreen SHOPPING AT A MARKET/ GARAGE SALE/ OUTDOORS

50 NoScreen FESTIVAL/ CARNIVAL/ EXPO/ SHOW/ AMUSEMENT PARK

51 NoScreen TENNIS

52 NoScreen CRICKET

53 NoScreen GOLF

54 NoScreen NETBALL

55 NoScreen SOCCER

56 NoScreen BOWLS

57 NoScreen VOLLEYBALL

58 NoScreen FOOTBALL

59 NoScreen BASEBALL

60 NoScreen TABLE TENNIS

61 NoScreen SOFTBALL

62 NoScreen SPORT (OTHER)

63 NoScreen COACHING/ SUPERVISING SPORT

64 NoScreen BASKETBALL

65 NoScreen WALKING

66 NoScreen WALKING IN GARDEN

67 NoScreen BUSHWALKING

68 NoScreen SKATEBOARDING/ SKATING/ ROLLERBLADING

69 NoScreen EXERCISING

70 NoScreen BIKE RIDING

71 NoScreen HORSE RIDING

72 NoScreen KICKING THE FOOTY

73 NoScreen CLIMBING TREES

74 NoScreen RUNNING

75 NoScreen RACING PIGEONS

76 NoScreen TARGET SHOOTING/ SHOOTING/ ARCHERY

77 NoScreen FLYING A KITE

78 NoScreen JOGGING

79 NoScreen BADMINTON

80 NoScreen TRAMPOLINING

81 NoScreen DOG - WALKING/ WASHING/ PLAYING WITH

82 NoScreen TOBOGGAN RIDES

83 NoScreen PLAYING - WITH FRIENDS, KIDS

84 NoScreen MOTOR RACING/ MOTOR BIKE RIDING/ GO-CART RACING

85 NoScreen WORKING ON THE HOUSE

86 NoScreen WORKING AROUND THE HOUSE

87 NoScreen WORKING ON THE POOL

88 NoScreen WORKING ON THE CAR/ BOAT/ CARAVAN

89 NoScreen OTHER WORK - AT HOME

90 NoScreen WASHING/ HANGING WASHING

91 NoScreen PACKING/ UNPACKING/ SHIFTING/ MOVING

92 NoScreen SHOPPING

93 NoScreen FARM JOBS

94 NoScreen BUILDING RELATED JOBS

95 NoScreen GARDEN RELATED JOBS

96 NoScreen OTHER JOBS

97 NoScreen UNSPECIFIED PAID JOBS

98 NoScreen WORKING

99 NoScreen WASHING/ CLEANING CAR

100 NoScreen GARDENING

101 NoScreen LAWN MOWING

102 NoScreen WATERING

103 NoScreen SWIMMING

104 NoScreen OUTSIDE (UNSPEC)

997 Openend OTHER (SPECIFY)

998 Single CAN'T SAY

IF DID NOT SAY WHERE DOING ACTIVITY OUTDOORS ON SATURDAY (NOT CODE 998 ON Q30), **ASK**:

*[Single response]*

Q30B. Where were you when you were [**verbatim response - activity outdoors Q30**]?

INTERVIEWER NOTE: **READ OUT IF NECESSARY**

IF OTHER, HIGHLIGHT OTHER AND TYPE IN RESPONSE

1 AT THE BEACH/LAKE/RIVER

2 AT A LOCAL POOL

3 AT A BACKYARD POOL

4 AT A PUBLIC PARK/GARDEN

5 AT A PUBLIC PLAYGROUND

6 AT SCHOOL GROUNDS

7 AT A SPORTS CENTRE/GROUNDS

8 AT AN OUTDOOR CAFE/RESTAURANT/PUB

9 AT A MARKET/FAIR/OUTDOOR SHOPPING CENTRE

10 ON THE STREET/FOOTPATH/WALKWAY

11 AT HOME/FRIEND'S PLACE

12 AT HOLIDAY HOUSE/CAMPING GROUND/HOTEL/MOTEL

13 ON A FARM

14 IN THE BUSH

15 AT A RACE TRACK/MOTOCROSS TRACK

16 AT WORKPLACE

17 DRIVING/ TRAVELLING/ TOURING/ ON THE ROAD

997 OTHER (SPECIFY)

998 CAN'T SAY

ENDIF

**AMOUNT OF TIME OUTDOORS PEAK UV PERIOD - SATURDAY**

*[Quantity] {Min: 0, Max: 241, Can’t say value:241}*

Q31. About how much time in total did you spend out of doors on Saturday between #/11am and 3pm/10am and 2pm/ [**verbatim response - activity outdoors Q30**]?

TYPE IN THE TOTAL IN MINUTES.

|  |  |  |
| --- | --- | --- |

IF RESPONDENT GAVE ANSWER IN HOURS, CONVERT TO MINUTES.

IF STILL CAN'T SAY TYPE VALUE 241

1.5 HOURS = 90 MINUTES

2 HOURS = 120 MINUTES

2.5 HOURS = 150 MINUTES

3 HOURS = 180 MINUTES

3.5 HOURS = 210 MINUTES

4 HOURS = 240 MINUTES

IF Q31 ANSWER IS 0 MINUTES, **SAY:**

Earlier you mentioned that you were outdoors on Saturday between #/11am and 3pm/10am and 2pm/ for longer than 15 minutes and that the activity you were mostly doing was [**verbatim response - activity outdoors Q30**]. About how much time in total did you spend out of doors on Saturday between #/11am and 3pm/10am and 2pm/ [**verbatim response - activity outdoors Q30**].

INTERVIEWER NOTE: IF RESPONDENT HAD INCORRECTLY ANSWERED CURRENT QUESTION, GO BACK ONE SCREEN AND CHANGE ANSWER AT CURRENT QUESTION.

 IF RESPONDENT INSISTS THAT ANSWER OF LESS THAN 15 MINUTES (INCLUDING ZERO) AT CURRENT QUESTION IS CORRECT, GO BACK AND RE-CONFIRM EARLIER QUESTIONS ABOUT BEING IN SUN FOR MORE THAN 15 MINUTES DURING PEAK SUNLIGHT PERIOD, AND THE MAIN ACTIVITY UNDERTAKEN DURING THAT PERIOD.

ENDIF

**LOCATION OUTDOOR ACTIVITY - SATURDAY**

IF Vic POSTCODE **ASK**:

Q32A. Were you in the Melbourne metropolitan area when you were [**verbatim response - activity outdoors Q30**]?

1 YES

2 NO (GO TO Q32I)

ENDIF

IF NSW POSTCODE **ASK**:

Q32B. Were you in the Sydney metropolitan area when you were [**verbatim response - activity outdoors Q30**]?

1 YES

2 NO (GO TO Q32I)

ENDIF

IF Qld POSTCODE **ASK**:

Q32C. Were you in the Brisbane metropolitan area when you were [**verbatim response - activity outdoors Q30**]?

1 YES

2 NO (GO TO Q32I)

ENDIF

IF WA POSTCODE **ASK**:

Q32D. Were you in the Perth metropolitan area when you were [**verbatim response - activity outdoors Q30**]?

1 YES

2 NO (GO TO Q32I)

ENDIF

IF SA POSTCODE **ASK**:

Q32E. Were you in the Adelaide metropolitan area when you were [**verbatim response - activity outdoors Q30**]?

1 YES

2 NO (GO TO Q32I)

ENDIF

IF Tas POSTCODE **ASK**:

Q32F. Were you in the Hobart metropolitan area when you were [**verbatim response - activity outdoors Q30**]?

1 YES

2 NO (GO TO Q32I)

ENDIF

IF ACT POSTCODE **ASK**:

Q32G. Were you in the Canberra metropolitan area when you were [**verbatim response - activity outdoors Q30**]?

1 YES

2 NO (GO TO Q32I )

ENDIF

IF NT POSTCODE **ASK**:

Q32H. Were you in the Darwin metropolitan area when you were [**verbatim response - activity outdoors Q30**]?

1 YES

2 NO (GO TO Q32I)

ENDIF

IF NOT IN THE ***METROPOLITAN* AREA** CODE 2 Q32 A-H for Sunday activity between #/11am and 3pm/10am and 2pm, ASK:

*[Single response]*

Q32I. What was the nearest suburb or town when you were [**verbatim response - activity outdoors Q30**]

997 OTHER (SPECIFY)

998 [Single] CAN'T SAY

ENDIF

**SUN PROTECTION DURING OUTDOOR ACTIVITY - SATURDAY**

**SHADE - SATURDAY**

*[Single response]*

Q33B. Were you MOSTLY IN THE SHADE or MOSTLY OUT IN THE OPEN while you were [**verbatim response - activity outdoors Q30**]?

1 IN THE SHADE

2 IN THE OPEN

3 IN SHADE AND OUT IN OPEN EQUALLY

4 CAN'T SAY

**CLOTHING - SATURDAY**

*[Single response]*

Q34A1. Would you mind telling me what you were wearing while you were [**verbatim response - activity outdoors Q30**]?

IF RESPONDENT HESITATES, **SAY**:

Can you remember what you were wearing on the top part of your body?

RETURN FOR CLOTHING WORN ON LOWER PART OF BODY

1 TOP/DRESS/WETSUIT

2 SWIMWEAR

3 TOPLESS

*[Single response]*

Q34A2.CLOTHING WORN ON LOWER PART OF BODY

RECORD OR IF RESPONDENT HESITATES **PROMPT WITH**:

Can you remember what you were wearing on the lower part of your body?

1 TROUSERS/JEANS/SHORTS/SKIRT/DRESS/WETSUIT

2 SWIMWEAR

3 BOTTOMLESS

IF TOP/DRESS/WETSUIT (CODE 1 ON Q34A1), **ASK**

*[Single response]*

Q34B. How long were the sleeves of your top/dress/wetsuit?

IF RESPONDENT HESITATES, **READ OUT**:

1 WRIST LENGTH

2 3/4 LENGTH

3 ELBOW LENGTH

4 SHORT

5 SLEEVELESS

ENDIF

IF TROUSERS/JEANS/SHORTS/SKIRT/DRESS/WETSUIT (CODE 1 ON Q34A2), **ASK**

*[Single response]*

Q34C. How long were/was your trousers/jeans/shorts/skirt/dress /wetsuit?

IF RESPONDENT HESITATES, **READ OUT**:

1 ANKLE LENGTH

2 3/4 LENGTH

3 KNEE LENGTH

4 MINI SKIRT/SHORT SHORTS

ENDIF

IF SWIMWEAR (CODE 2 ON Q34A1 OR CODE 2 ON Q34A2), **ASK**

*[Multiple]*

Q34D. What sort of swimwear/bathers were you wearing?

1 ONE PIECE BATHERS

2 TWO PIECE/BIKINI

3 BIKINI TOP ONLY

4 BIKINI BOTTOM ONLY

5 LONG/BOARD SHORTS

6 SHORT SHORTS

7 BRIEFS/SPEEDOS

8 RASH VEST

ENDIF

**HEAD WEAR - SATURDAY**

*[Single response]*

Q34G.Were you wearing a cap, hat or sun visor?

1 HAT

2 CAP

3 VISOR

4 NONE WORN

IF HAT OR CAP WORN (CODES 1 OR 2 ON Q34G), **ASK**

*[Single response]*

Q34H.Did your #/hat/cap/ have a wide brim or a narrow brim?

1 WIDE BRIM

2 NARROW BRIM

3 NO BRIM

ENDIF

IF HAT, CAP OR VISOR WORN (CODE 1 OR 2 OR 3 ON Q34G), **ASK**

*[Single response]*

Q34H2. Did it have a flap which covered the back of your neck?

1 YES

2 NO

ENDIF

**SUNGLASSES - SATURDAY**

*[Single response]*

Q34I.Were you wearing any sunglasses?

1 YES

2 NO

**SUNSCREEN - SATURDAY**

*[Single response]*

Q47. Now some questions about sunscreen.

A sunscreen is a gel, lotion or cream that filters out ultraviolet sunlight to prevent burning and other skin damage.

Did you use a sunscreen between #/11am and 3pm/10am and 2pm/ on Saturday?

1 YES, USED SUNSCREEN

2 NO, DIDN'T USE SUNSCREEN

3 MAKEUP WITH A SUNSCREEN ONLY

IF USED SUNSCREEN (CODE 1 OR 3 ON Q47) **ASK**:

*[Single response]*

Q48A.Did you apply the sunscreen:

IF RESPONDENT ANSWERS SELECT "as soon as I went out in the sun

IF RESPONDENT ANSWERS SELECT "after slightly sun burnt

**(READ OUT)**

1 BEFORE GOING OUT IN THE SUN

2 AFTER YOU'D BEEN IN THE SUN A WHILE

3 **DO NOT READ OUT** - AFTER SLIGHTLY SUN BURNT

*[Quantity] {Min: 0, Max: 99, Default Value:99}*

Q49B.What was the sun protection factor of the sunscreen you used?

|  |  |  |
| --- | --- | --- |

IF RESPONDENT SAYS "15"+ ENTER "16".

IF RESPONDENT SAYS "30"+ ENTER "31".

If RESPONDENT SAYS “50”+ ENTER “51”.

IF "DON'T KNOW" type value “99”.

*[Multiple]*

Q50A. On what areas of the body did you apply sunscreen?

Where else?

Anywhere else?

PROMPT: Did you apply sunscreen to your...

READ OUT LIST (EXCLUDING ANSWERS GIVEN)

1 FACE

2 NOSE

3 **(DO NOT READ)** HEAD

4 **(DO NOT READ)** EARS

5 CHEST

6 STOMACH

7 BACK

8 NECK

9 SHOULDERS

10 ARMS

11 HANDS

12 LEGS

13 BACK OF KNEES

14 FEET

ENDIF

**EXPOSED SKIN - SATURDAY**

*[Multiple]*

Q50B. Were there any areas of your body exposed to the sun that didn't have sunscreen, clothing or a hat covering them?

**READ OUT IF HESITATES**

1 FACE

2 NOSE

3 HEAD

4 EARS

5 CHEST

6 STOMACH

7 BACK

8 NECK

9 SHOULDERS

10 ARMS

11 HANDS

12 LEGS

13 BACK OF KNEES

14 FEET

15 NONE

**SHADE AVAILABLE OUTDOOR ACTIVITY - SATURDAY**

IF ENGAGED IN ACTIVITIES ON SATURDAY AND MOSTLY IN OPEN (CODE 1 TO 997 ON Q30 AND CODE 2 ON Q33B), **ASK**:

*[Single response]*

Q42. Earlier, you said that you were mostly out in the open when you were [**verbatim response - activity outdoors Q30**] outdoors on Saturday between #/11am and 3pm/10am and 2pm/ . It may sound odd, but, would it have been possible for you to [**verbatim response - activity outdoors Q30**] mostly in the shade or indoors?

1 YES

2 NO

3 CAN'T SAY

ENDIF

IF NOT POSSIBLE (CODE 2 ON Q42), **ASK**:

*[Multiple]*

Q43A. Why was it not possible for you to [**verbatim response - activity outdoors Q30**] mostly in the shade or indoors?

Any other reason?

Any others?

IF OTHER, HIGHLIGHT OTHER AND TYPE IN RESPONSE

1 NO SHADE AVAILABLE AT LOCATION

2 NO INDOOR FACILITY AVAILABLE

3 DIDN'T TAKE PORTABLE SHADE (E.G. UMBRELLA, PARASOL, SHADE CLOTH)

4 JUST IMPOSSIBLE TO DO IN THE SHADE

5 JUST IMPOSSIBLE TO DO INDOORS

6 NoScreen I CHOSE TO BE IN THE SUN

97 OTHER (SPECIFY)

98 [Single] CAN'T SAY

ENDIF

IF ENGAGED IN ACTIVITIES ON SATURDAY AND MOSTLY IN SHADE (CODE 1 OR 3 ON Q33B AND Q30 IS 1-997), **ASK**:

*[Multiple] {Spread:10 }*

Q43D. Earlier, you said that you were #/mostly in the shade// #/in the shade and out in the open equally// when you were [**verbatim response - activity outdoors Q30**] outdoors on Saturday between #/11am and 3pm/10am and 2pm/ . What type of shade from the sun did you use?

IF OTHER, SELECT OTHER AND TYPE IN RESPONSE

1 OUTDOOR SHADE STRUCTURES (E.G. VERANDAHS, GAZEBOS, SHADE SAILS, ETC.)

2 PORTABLE SHADE (E.G. UMBRELLAS, PARASOLS, SHADE SHELTERS, TENTS, ETC.)

3 TREES, HEDGES, ETC.

5 NoScreen SHADE FROM BUILDINGS/ HOUSES

97 OTHER (SPECIFY)

98 [Single] CAN'T SAY

ENDIF

**ENDIF**

**TOTAL TIME OUTDOORS – SATURDAY**

*[Quantity] {Min: 0, Max: 841, Can’t say type value: 999}*

Q29A. Thinking about the WHOLE day on Saturday not just the time between #/11am and 3pm/10am and 2pm/ - about how much time did you spend out of doors? By out of doors we mean not in a building and not in a covered vehicle.

|  |  |  |
| --- | --- | --- |

TYPE IN TOTAL IN MINUTES!

IF RESPONDENT GAVE ANSWER IN HOURS, CONVERT TO MINUTES.

IF CAN'T SAY, **ASK**:

Well, could you estimate to the NEAREST 1/2 HOUR how much time you spent out of doors on Saturday?

TYPE TIME IN MINUTES

IF STILL CAN'T SAY TYPE 999

1.5 HOURS = 90 MINUTES 2 HOURS = 120 MINUTES

2.5 HOURS = 150 MINUTES 3 HOURS = 180 MINUTES

3.5 HOURS = 210 MINUTES 4 HOURS = 240 MINUTES

4.5 HOURS = 270 MINUTES 5 HOURS = 300 MINUTES

5.5 HOURS = 330 MINUTES 6 HOURS = 360 MINUTES

6.5 HOURS = 390 MINUTES 7 HOURS = 420 MINUTES

7.5 HOURS = 450 MINUTES 8 HOURS = 480 MINUTES

8.5 HOURS = 510 MINUTES 9 HOURS = 540 MINUTES

If >14 hours type value 841

**INTERVIWER CROSS-CHECK CONSISTENCY WITH Q29B & Q31 revised:**

Earlier you mentioned that you were outdoors on Saturday between #/11am and 3pm/10am and 2pm/ for longer than 15 minutes. Now if you were to think about the WHOLE day on Saturday, how much time did you spend out of doors?

 INTERVIEWER NOTE: IF RESPONDENT HAD INCORRECTLY ANSWERED CURRENT QUESTION, GO BACK ONE SCREEN AND CHANGE ANSWER AT CURRENT QUESTION.

 IF RESPONDENT INSISTS THAT ANSWER OF LESS THAN 15 MINUTES AT CURRENT QUESTION IS CORRECT, MARK THE BOX BELOW.

Q29AN - Q29AN

INTERVIEWER NOTE: IF RESPONDENT CHANGES THE ANSWER FOR Q29A or Q29B, GO BACK AND CHANGE THE ANSWER NOW.

 IF RESPONDENT DOES NOT WANT TO CHANGE THE ANSWER, ENTER TO CONTINUE

|  |
| --- |

***ASK EVERYONE***

**SUN-RELATED ATTITUDES**

I am now going to read out a number of statements. For each statement, I'd like to know how much you agree or disagree.

*[Single response]*

Q52C. Do you agree or disagree with the statement "A suntanned person LOOKS more healthy".

**(READ OUT):**

Do you ... Strongly Agree, Mildly Agree,

.. Neither Agree Nor Disagree

.. Mildly Disagree or Strongly Disagree

1 STRONGLY AGREE

2 MILDLY AGREE

3 NEITHER AGREE NOR DISAGREE

4 MILDLY DISAGREE

5 STRONGLY DISAGREE

6 CAN'T SAY

*[Single response]*

Q52E.(Do you agree or disagree with the statement)

"A suntanned person IS more healthy".

IF NEEDED PROMPT:

Do you ...# Strongly Agree, Mildly Agree,

..# Neither Agree Nor Disagree

# Mildly Disagree or Strongly Disagree

1 STRONGLY AGREE

2 MILDLY AGREE

3 NEITHER AGREE NOR DISAGREE

4 MILDLY DISAGREE

5 STRONGLY DISAGREE

6 CAN'T SAY

*[Single response]*

Q52H.(Do you agree or disagree with the statement)

"Most of my CLOSE FAMILY think that a suntan is a good thing".

IF NEEDED PROMPT:

Do you ...# Strongly Agree, Mildly Agree,

..# Neither Agree Nor Disagree

# Mildly Disagree or Strongly Disagree

1 STRONGLY AGREE

2 MILDLY AGREE

3 NEITHER AGREE NOR DISAGREE

4 MILDLY DISAGREE

5 STRONGLY DISAGREE

6 CAN'T SAY

*[Single response]*

Q52Z. (Do you agree or disagree with the statement)

"If I regularly protect myself from the sun, I can avoid skin cancer".

IF NEEDED PROMPT:

Do you ...# Strongly Agree, Mildly Agree,

..# Neither Agree Nor Disagree

# Mildly Disagree or Strongly Disagree

1 STRONGLY AGREE

2 MILDLY AGREE

3 NEITHER AGREE NOR DISAGREE

4 MILDLY DISAGREE

5 STRONGLY DISAGREE

6 CAN'T SAY

*[Single response]*

Q52J. (Do you agree or disagree with the statement)

"Most of my FRIENDS think a suntan is a good thing".

IF NEEDED PROMPT:

Do you ...# Strongly Agree, Mildly Agree,

..# Neither Agree Nor Disagree

# Mildly Disagree or Strongly Disagree

1 STRONGLY AGREE

2 MILDLY AGREE

3 NEITHER AGREE NOR DISAGREE

4 MILDLY DISAGREE

5 STRONGLY DISAGREE

6 CAN'T SAY

**PERSONAL SKIN CANCER RISK**

*[Single response]*

QSC1. Reflecting on your lifestyle up to now, what do you think is your chance of developing skin cancer some day? Please give a rating from 0 to 10, where 0 is no chance at all, and 10 is will definitely develop skin cancer.

1 0 - NO CHANCE AT ALL

2 1

3 2

4 3

5 4

6 5

7 6

8 7

9 8

10 9

11 10 - I WILL DEFINITELY DEVELOP SKIN CANCER

**REASONS SUNBURNT THIS WEEKEND**

IF SUNBURNT (SINGLE RESPONSE ON Q4B OR RESPONSE ON Q4C), **SAY:**

Returning to the issue of sunburn. I want to find out a little bit more about how your body got sunburnt on the weekend.

*[Multiple]*

Q51. You said you got sunburnt on #/Saturday// #/and// #/Sunday//. How do you think you got sunburnt?

IF OTHER, SELECT OTHER AND TYPE IN RESPONSE

1 TRYING TO GET A SUN TAN

2 FORGOT TO PROTECT

3 DIDN'T THINK NEEDED TO PROTECT

4 COULD NOT BE BOTHERED PROTECTING

5 MISSED AREA WHEN APPLYING SUNSCREEN

6 SUNSCREEN WORE OFF

7 BURNT THROUGH SUNSCREEN

8 BURNT THROUGH CLOTHING

9 STAYED IN SUN TOO LONG

10 DIDN'T WEAR CLOTHING/DIDN'T USE SUNSCREEN OR OTHER SUN PROTECTION

11 SUNNY DAY/HOT TEMPERATURE

97 OTHER (SPECIFY)

98 [Single] CAN'T SAY

ENDIF

**(STATE/TERRITORY) TVC CAMPAIGN EVALUATION**

*Now I am going to ask you some questions about advertisements on television.*

***ASK ALL WA RESPONDENTS:***

*[Single response]*

QAD1 Do you remember seeing any video advertisements on TV about a young man Wes Bonny who died of melanoma? The TV ads feature his family and friends talking about how he was like other young men and loved the outdoors and going to the beach. He died soon after his brother’s wedding. Do you remember ever seeing these advertisements?

1 YES

2 NO

3 CAN'T SAY

IF REMEMBERS SEEING ADVERTISEMENT (CODE 1 ON QAD1),

**ASK :**

*[Multiple] {Spread:20 }*

QAD2. Can you tell me the main message in these advertisements? Can you tell me any other messages?

INTERVIEWER NOTE: FULL PROBING TO RECORD ALL MESSAGES RECALLED I.E. Repeat ‘CAN YOU TELL ME ANY OTHER MESSAGES?’

HIGHLIGHT ALL MENTIONED

IF OTHER, HIGHLIGHT OTHER AND TYPE IN RESPONSE

(CODED Response categories are to be confirmed)

1 SKIN CANCER/MELANOMA CAN AFFECT ANYBODY

2 YOUNG PEOPLE ARE AT RISK/CAN DIE FROM SKIN CANCER/MELANOMA

3 SKIN CANCER/MELANOMA CAN SPREAD

4 SKIN CANCER/MELANOMA IS DANGEROUS/CAN KILL

5 INCIDENTAL SUN EXPOSURE CAN CAUSE SKIN CANCER/MELANOMA

6 SKIN CANCER/MELANOMA CAN IMPACT YOUR FAMILY/FRIENDS

7 ‘YOU KNOW WHAT TO DO. DO IT’

8 USE SUN PROTECTION/PROTECT YOUR SKIN

97 OTHER (PLEASE SPECIFY)

98 [Single] CAN'T SAY

ENDIF

IF REMEMBERS SEEING ADVERTISEMENT (CODE 1 ON QAD1),

**ASK**

*[Single response]*

QAD7. ( Do you agree or disagree with the statement:) This Ad made me more likely to protect myself from the sun.

INTERVIEWER NOTE: IF AGREE, SAY: Is it strongly agree or mildly agree?

INTERVIEWER NOTE: IF DISAGREE, SAY: Is it strongly disagree or mildly disagree?

1 STRONGLY AGREE

2 MILDLY AGREE

3 NEITHER AGREE/DISAGREE

4 MILDLY DISAGREE

5 STRONGLY DISAGREE

6 CAN'T SAY

ENDIF

ENDIF

***ASK ALL VIC RESPONDENTS:***

*[Single response]*

*[Programming note: If interviewed during Week 1 (November 2016) program “this spring…”; if interviewed from Week 2 onward (i.e. all other dates) program “this spring or summer…”]*

QADVIC1. Do you remember seeing a television advertisement this spring (or summer) that shows a man doing everyday outdoor activities like mowing the lawn, walking his dog, hosting a BBQ, playing backyard cricket and working outdoors on a construction site? The man has a clock on his arm, which counts the time he spends in the sun unprotected. At the end of the advertisement the man is on a hospital bed, with the clock on his arm flashing red as he is wheeled into surgery.

IF NEEDED PROMPT: Do you remember seeing this advertisement this summer?

1 YES

2 NO

3 CAN'T SAY

IF REMEMBERS SEEING ADVERTISEMENT (CODE 1 ON QADVIC1), **ASK**

*[Multiple] {Spread:20 }*

QADVIC2. Can you tell me the main message in these advertisements? Can you tell me any other messages?

1 UV ADDS UP (SLOGAN)

2 THE MORE TIME YOU SPEND IN THE SUN UNPROTECTED, THE GREATER THE RISK OF CANCER

3 EVEN A SMALL AMOUNT OF UV/SUN EXPOSURE IS HARMFUL/CAN CAUSE SKIN CANCER

4 YOU SPEND MORE TIME IN THE SUN THAN YOU THINK

5 EVERYDAY ACTIVITIES ARE A SIGNIFICANT SOURCE OF UV/SUN EXPOSURE/SKIN DAMAGE

6 UV RADIATION (IN GENERAL) IS HARMFUL/DAMAGING/CAN CAUSE SKIN CANCER

7 USE SUN PROTECTION/PROTECT YOUR SKIN/DON’T GET TOO MUCH SUN

8 THE CLAIMS IN THE AD ARE EXAGGERATED

9 SKIN CANCER KILLS/CAN AFFECT THOSE WE LOVE

10 OTHER

11 DON’T KNOW/CAN’T SAY

*[Single response]*

QAD7. ( Do you agree or disagree with the statement:) This Ad made me more likely to protect myself from the sun.

INTERVIEWER NOTE: IF AGREE, SAY: Is it strongly agree or mildly agree?

INTERVIEWER NOTE: IF DISAGREE, SAY: Is it strongly disagree or mildly disagree?

1 STRONGLY AGREE

2 MILDLY AGREE

3 NEITHER AGREE/DISAGREE

4 MILDLY DISAGREE

5 STRONGLY DISAGREE

6 CAN'T SAY

ENDIF

***ASK ALL NSW AND ACT RESPONDENTS:***

*[Single]*

QAD1 Do you remember seeing a video advertisement on TV about a young girl who starts as a baby and moves through childhood to adulthood spending her time outdoors. The advertisement shows how skin cancer develops in the body. Do you remember ever seeing this advertisement?

1 YES

2 NO

3 CAN'T SAY

*IF REMEMBERS SEEING ADVERTISEMENT (CODE 1 ON QAD1),*

*ASK Qs AD2 TO AD8:*

*[Multiple] {Spread:20 }*

QAD2. Can you tell me the main message in these advertisements? Can you tell me any other messages?

INTERVIEWER NOTE: FULL PROBING TO RECORD ALL MESSAGES RECALLED I.E. Repeat ‘CAN YOU TELL ME ANY OTHER MESSAGES?’

HIGHLIGHT ALL MENTIONED

IF OTHER, HIGHLIGHT OTHER AND TYPE IN RESPONSE

*(CODED Response categories are to be confirmed)*

1 SKIN CANCER/MELANOMA CAN AFFECT ANYBODY

2 YOUNG PEOPLE ARE AT RISK/CAN DIE FROM SKIN CANCER/MELANOMA

3 SKIN CANCER/MELANOMA CAN SPREAD

4 SKIN CANCER/MELANOMA IS DANGEROUS/CAN KILL

5 INCIDENTAL SUN EXPOSURE CAN CAUSE SKIN CANCER/MELANOMA

6 UV RADIATION GETS INTO YOUR SKIN

7 UV RADIATION CAUSES DNA DAMAGE

8 SOME DNA DAMAGE CAUSED BY UV RADIATION IS PERMANENT

9 DNA DAMAGE CAUSES MELANOMA

10 MELANOMA CAN COME UP AS CANCER IN OTHER PARTS OF THE BODY

11 CHEMOTHERAPY CAN BE PART OF THE TREATMENT FOR MELANOMA

12 USE SUN PROTECTION/PROTECT YOUR SKIN

97 OTHER (PLEASE SPECIFY) _______________________

98 [Single] CAN'T SAY

*[Single]*

QAD7. ( Do you agree or disagree with the statement:) This Ad made me more likely to protect myself from the sun.

INTERVIEWER NOTE: IF AGREE, SAY: Is it strongly agree or mildly agree?

INTERVIEWER NOTE: IF DISAGREE, SAY: Is it strongly disagree or mildly disagree?

1 STRONGLY AGREE

2 MILDLY AGREE

3 NEITHER AGREE/DISAGREE

4 MILDLY DISAGREE

5 STRONGLY DISAGREE

6 CAN'T SAY

ENDIF

**SUN SOUND CAMPAIGN**

***ASK ALL ALL WA and ALL QLD RESPONDENTS, ASK ALL NSW NSPS SAMPLE EXCEPT SYDNEY YOUTH BOOST:***

[Program the appropriate jingle for each state],

QSUNSOUND#. Do you remember hearing a jingle on loud speakers with the words ‘Be Sun Sound’ when you were outdoors this summer, either at the beach or pool?

I am going to play the Sun Sound Jingle now. [PLAY JINGLE ] Do you remember ever hearing this jingle?

1 YES

2 NO

3 CAN'T SAY

*[Multiple]*

ASK if CODE 1 on QSUNSOUND#:

QSUNSOUND3. Where were you when you heard it?

1 NAME OF BEACH OR POOL (SPECIFY)____________________________

2 OTHER LOCATION (SPECIFY)____________________________________

3 ON THE RADIO

4 CAN’T REMEMBER

ASK if CODE 1 on QSUNSOUND#:

*[Single response]*

QSUNSOUND4. Can you tell me what the main message of the jingle is?

1 PROTECT YOURSELF FROM THE SUN

2 BE SUNSMART

3 REAPPLY SUNSCREEN

4 OTHER_______________

5 DON'T KNOW

ENDIF

ENDIF

**ASK ALL Sydney Youth Boost Sample aged 12-24 years**

**PRETTY SHADY CAMPAIGN**

*Pretty Shady is a youth brand that focuses on skin cancer and sun safety. It features celebrities, prize giveaways and the branding is yellow and black in colour.*

*[Single]*

QPSHAD1. Do you remember seeing, reading or hearing anything about Pretty Shady? This may have been an advertisement on TV or the internet, on social media, a logo, or something else.

1 YES

2 NO

3 CAN'T SAY

*IF REMEMBERS SEEING ADVERTISEMENT (CODE 1 ON QPSHAD1),*

*ASK QPSHAD2 and QPSHAD3:*

*[Multiple] {Spread:20 }*

QPSHAD2. Can you tell me the main message of Pretty Shady? Can you tell me any other messages?

INTERVIEWER NOTE: FULL PROBING TO RECORD ALL MESSAGES RECALLED I.E. Repeat ‘CAN YOU TELL ME ANY OTHER MESSAGES?’

HIGHLIGHT ALL MENTIONED

IF OTHER, HIGHLIGHT OTHER AND TYPE IN RESPONSE

1 ‘BE PART OF THE GENERATION THAT STOPS SKIN CANCER ONE SUMMER AT A TIME’

2 ‘ITS THE MOST COMMON CANCER AFFECTING YOUNG AUSTRALIANS’

3 ‘2 IN 3 OF YOUR FRIENDS WILL BE DIAGNOSED AT SOME POINT IN THEIR LIVES’

4 ‘THOUSANDS ARE DYING EACH YEAR’

5 ‘STOP THE SUN DAMAGE, STOP 95% OF MELANOMAS’

6 CELEBRITY AMBASSADORS – GENERAL

7 GET INVOLVED WITH PRETTY SHADY (e.g. sign up or visit the Pretty Shady website)

8 USE SUN PROTECTION / PROTECT YOUR SKIN

9 USE PRETTY SHADY PRODUCTS (e.g. sunscreen, shirt, umbrella, sunglasses, hat)

10 PRETTY SHADY SUN PROTECTION PRODUCTS ARE FREE / WIN PRETTY SHADY SUN PROTECTION

PRODUCTS

97 OTHER (PLEASE SPECIFY) _______________________

98 *[Single]* CAN'T SAY

*[Multiple]*

QPSHAD4.

*In the next questions let me know the type of things you have done or thought about doing after seeing, reading or hearing about Pretty Shady.*

*Multiple response categories*

1 YES

2 NO

3 CAN'T SAY

*ROTATE:*

READ OUT:

1 INCREASE MY LEVEL OF SUN PROTECTION

2 VISIT A DOCTOR TO HAVE MY SKIN CHECKED

3 CHECK MY OWN SKIN FOR SKIN CANCER

4 TALK TO FAMILY AND FRIENDS ABOUT SUN PROTECTION OR SKIN CANCER

5 USE FAKE TANNING LOTIONS, SPRAYS OR CREAMS INSTEAD OF SUNTANNING

6 VISIT THE PRETTY SHADY WEBSITE/FACEBOOK PAGE

7 SIGN UP TO PRETTY SHADY

***ASK EVERYONE***

**SOLARIUMS**

**Lifetime use**

*[Single response]*

Qsol1. Have you EVER used a solarium?

1 YES

2 NO

3 CAN'T SAY

**Recent use**

Qsol#1. Have you used a solarium, sunbed or other UV tanning device in the past 12 months?

1 YES

2 NO

3 CAN'T SAY

***ASK IF CODE 1 ON Qsol#1***

*[Multiple response]*

Qsol#2. Thinking about the past 12 months at which of the following locations did you use the sunbed or other UV tanning device?

(READ OUT)

1 AT YOUR HOME OR A FRIENDS’ OR FAMILY MEMBER’S HOME IN AUSTRALIA

2 WHILE TRAVELLING OVERSEAS

3 AT A COMMERCIAL SOLARIUM, TANNING SALON, FITNESS CENTRE, HAIRDRESSERS OR OTHER BUSINESS IN AUSTRALIA

4 OTHER LOCATION (PLEASE SPECIFY**)____________________________________**

END IF

**PEER TANNING NORMS – Adolescent respondents only**

***IF AGED 12 – 17 YEARS ASK:***

*[Single response]*

Q58B. Of your best friends who are male, do most of them try to get a tan?

1 YES

2 NO

3 CAN'T SAY

*[Single response]*

Q58C. Of your best friends who are female, do most of them try to get a tan?

1 YES

2 NO

3 CAN'T SAY

ENDIF

**TAN ATTITUDES for Sydney Youth Boost Sample**

*IF SYDNEY BOOSTER SAMPLE Aged 12-24 years ASK*

***ROTATE STATEMENTS***

QNSW. I will now read out a series of statements.  For each statement, could you please tell me whether you strongly agree, mildly agree, neither agree nor disagree, mildly disagree or strongly disagree. The first statement is….

(STATEMENTS)

QNSW1. A suntan protects you against melanoma and other skin cancers

QNSW2. This summer I intend to sunbathe regularly to get a suntan

QNSW3. I feel more healthy with a suntan

QNSW4. A suntan makes me feel better about myself

QNSW5. A suntan makes me feel more attractive to others

(RESPONSE FRAME)

1. STRONGLY AGREE
2. MILDLY AGREE
3. NEITHER AGREE NOR DISAGREE
4. MILDLY DISAGREE
5. STRONGLY DISAGREE
6. CAN'T SAY

ENDIF

**INTENTIONAL AND INCIDENTAL TANNING**

***ASK EVERYONE***

*[Single response]*

QINCIDENTAL 1. This summer how many days did you spend more than 1 hour outside per day between 9am and 5pm?

1 I NEVER SPENT MORE THAN 1 HOUR OUTSIDE DURING THESE TIMES THIS SUMMER

2 1-3 DAYS SO FAR THIS SUMMER

3 4-5 DAYS SO FAR THIS SUMMER

4 6 OR MORE DAYS SO FAR THIS SUMMER

5 CAN’T SAY

IF CODE 2 TO 4 ON QINCIDENTAL1, **ASK**

QINCIDENTAL 2. On the last sunny day you spent more than 1 hour outside between 9am and 5pm did you protect your arms, face and most of your body from the sun with sunscreen, clothing or a hat, or mostly stayed under shade?

1 YES

2 NO

3 CAN’T SAY

END IF

***ASK EVERYONE***

QTAN1. Is your skin tanned at all?

1 YES

2 NO

3 CAN’T SAY

QTAN2. Thinking about the past year did you intentionally try to maintain a tan at all?

1 YES

2 NO

3 CAN’T SAY

*IF RESPONDS ‘YES’ ON QTAN2****, ASK:***

QTAN4. Over the past year, which of the following options did you use to maintain a tan? (multiple response)

1 SUNBAKING

2 SPRAY TAN

3 TANNING LOTIONS

4 SOLARIUM

5 HOME TANNING BED OR HOME SUN BED

97 OTHER (SPECIFY)

END IF

***ASK EVERYONE***

*[Single response]*

*[Programming note: If interviewed during Week 1 (November 2016) program “this spring…”; if interviewed from Week 2 onward (i.e. all other dates) program “this spring and summer…”]*

QINCIDSPB#1 This spring (and summer) how often did you use sun protection in your everyday activities when you were outside for 10 minutes or more? Would that be….

**(READ OUT)**

1 NEVER

2 RARELY

3 SOMETIMES

4 MOST OF THE TIME

5 ALL THE TIME

**WORK OUTDOORS**

*IF AGED 15 OR OVER,* **ASK**:

NOW I'd like to ask you a few questions about work.

*[Single response]*

Qjob1.Are you now in paid employment?

Interviewer note: IF “YES” **ASK**: Is that full-time for 35 hours or more a week, or part-time?

Interviewer note: IF “NO” **ASK**: Are you retired, studying or home duties?

1 YES - FULL-TIME

2 YES - PART-TIME

3 NO - HOME DUTIES/DON'T WORK

4 NO - AT SCHOOL/STUDYING FULL TIME

5 NO - RETIRED

IF RESPONDENT WORKS (CODE 1 OR 2 ON Qjob1), **ASK**:

*[Single response]*

Qjob2#. When you're working at your job on a typical workday in summer, do you spend any time outside?

1 YES

2 NO

3 CAN'T SAY

**SUN PROTECTION POLICY & ENVIRONMENT**

IF RESPONDENT WORKS OUTDOORS (CODE 1 on Qjob2#. ASK:

**Which of the following are provided at your workplace?**

*[Single response]*

Q61A. (Which of the following are provided at your workplace?)

**Sunscreen**

1 YES

2 NO

3 CAN'T SAY

*[Single response]*

Q62. Does your workplace have a policy to encourage workers to protect themselves from the sun when they work outside?

1 YES

2 NO

3 CAN'T SAY

**ENDIF**

***ASK EVERYONE***

**EARLY DETECTION ITEMS**

**SAY:**

Now the next question is about checking your skin for skin cancer.

*[Single response]*

QED. In the last 12 months, has a doctor checked at least some of your skin for any suspicious spots that might be skin cancer?

Interviewer note: (IF MORE THAN ONCE, PLEASE REFER TO THE MOST RECENT SKIN CHECK)

IF YES AND NEEEDED ASK: Is that a check of all or nearly all your body? Or was it only part of the body or a specific mole or spot?

1 YES A GENERAL CHECK OF ALL OR NEARLY ALL OF MY BODY (IE. DOWN TO UNDERWEAR)

2 YES A GENERAL CHECK OF PART OF MY BODY

3 YES A CHECK OF A SPECIFIC MOLE OR SPOT

4 NO

**UV KNOWLEDGE**

*[Multiple]*

QUV1. Which of the following measures would be most useful to tell you the risk of sunburn for the day?

**(Rotate response list)**

**(READ OUT)**

1 TEMPERATURE

2 CLOUD COVER

3 UV INDEX

4 WIND CONDITIONS

5 HUMIDITY

6  **(DO NOT READ OUT)** CAN’T SAY

*[Quantity]*

QUV2. The UV Index ranges from zero upwards. As far as you know at or above what UV Index value would you say that you need to protect yourself from the sun?

|  |  |  |
| --- | --- | --- |

998 **(DO NOT READ OUT)** CAN’T SAY

***ASK ALL SA RESPONDENTS:***

The next question is about a television advertisement.

*[Single response]*

*[Programming note: If interviewed during Week 1 (November 2016) program “this spring…”; if interviewed from Week 2 onward (i.e. all other dates) program “this spring or summer…”]*

QADSA1. Do you remember seeing a television advertisement this spring (or summer) that featured a cartoon character walking his dog in the sun and telling people about the UV index and when to use sun protection?

1 YES

2 NO

3 CAN’T SAY

**ENDIF**

**BELIEFS ABOUT SUNSCREENS**

*[Single response]*

QNANO1. Do you agree or disagree with the statement 'All sunscreens can be used safely on a daily basis to protect your skin when out in the sun. By safely we mean with no harm to your health.'?

**IF AGREE**, SAY: Is it strongly agree or mildly agree?

**IF DISAGREE**, SAY: Is it strongly disagree or mildly disagree?

1 STRONGLY AGREE

2 MILDLY AGREE

3 NEITHER AGREE/DISAGREE

4 MILDLY DISAGREE

5 STRONGLY DISAGREE

6 CAN'T SAY

QENV#. Are the following statements about sunscreens true or false?

**(ROTATE)**

QENV2. People who use sunscreen regularly when they are outdoors don’t get enough vitamin D from the sun.

1 TRUE

2 FALSE

3 CAN'T SAY

QENV3. Sunscreens can prevent skin cancer if used on a daily basis.

1 TRUE

2 FALSE

3 CAN'T SAY

QENV4. The ingredients in sunscreens are bad for your health if used regularly.

1 TRUE

2 FALSE

3 CAN'T SAY

**VITAMIN D ITEMS**

**IF RESPONDENT IS 18-69 YEARS ASK Qs VD 1-3 and SAY:**

The next question is about Vitamin D. The body needs Vitamin D to be healthy. You can get Vitamin D from sunlight and from some food sources including milk, or your GP can prescribe a Vitamin D supplement if you don't have enough.

*[Single response]*

QVD3. Thinking about the past few summers together, did you make any changes to the way you protect yourself from the sun to get more Vitamin D?

1 YES

2 NO

3 CAN’T SAY

**ENDIF**

**TV / Media Use**

*[Multiple response]*

*Multiple response categories*

*1 YES*

*2 NO*

*3 CAN'T SAY*

QMEDIA7. In the past week, which of the following did you spend any time doing:

**[READ OUT]**

1. WATCHING COMMERCIAL FREE-TO-AIR TELEVISION

2. WATCHING ‘CATCH-UP’ TELEVISION (SUCH AS TEN-PLAY, OR I-VIEW OR SBS ON DEMAND)

3. ON FACEBOOK

5. ON SNAPCHAT

**SunSmart APP USE**

IF THE RESPONDENT PERSONALLY HAS A MOBILE PHONE (CODE 1 QPHONE2) OR IS IN THE MOBILE PHONE SAMPLE, **ASK**:

*[Single response]*

QAPP1. SunSmart have a free smartphone App that provides information on the UV for the day and the times when sun protection is required.

Have you downloaded the SunSmart App?

1 YES

2 NO

3 Don’t have a smartphone

4 CAN'T SAY

IF YES (CODE 1 ON QAPP1), **ASK**:

*[Single response]*

*[Programming note: If interviewed during Week 1 (November 2016) program “this spring…”; if interviewed from Week 2 onward (i.e. all other dates) program “this spring or summer…”]*

QAPP2. Have you used the SunSmart App this spring (or summer)?

1 YES

2 NO

3 CAN'T SAY

**ENDIF**

***ASK EVERYONE***

SAY: Just to make sure we have interviewed a true cross-section of people, I'd like to ask you one final question.

**CURRENT/HIGHEST EDUCATION**

**IF AGED 12 – 17 (Quantity 12-17 on AGE1), ASK**:

*[Single response]*

Q64A. What year level are you in?

1. YEAR 6
2. YEAR 7
3. YEAR 8
4. YEAR 9
5. YEAR 10
6. YEAR 11
7. YEAR 12
8. LEFT SCHOOL
9. REFUSED
10. Year 5 (HELD BACK)

**ENDIF**

IF AGED 18+ (Quantity 18-69 on AGE1) OR, IF AGED 12-17 AND LEFT SCHOOL (Code 8 on Q64A), **ASK:**

*[Single response]*

Q56C. Could you please tell me your highest level of education?

1 SOME PRIMARY SCHOOL

2 FINISHED PRIMARY SCHOOL

3 SOME SECONDARY SCHOOL

4 SOME TECHNICAL OR COMMERCIAL

5 4TH FORM/INTERMEDIATE, 5TH FORM/LEAVING

6 TECHNICAL SCHOOL/TAFE

7 VCE/HSC OR MATRIC

8 SOME UNI/C.A.E.

9 TERTIARY DIPLOMA

10 CURRENTLY UNIVERSITY OR C.A.E.

11 TERTIARY DEGREE

**ENDIF**

Thank you for your time and assistance. This market research is carried out in compliance with the Privacy Act, and the information you provided will be used only for research purposes.

We are conducting this research on behalf of Cancer Council Australia.

If you would like any more information about this project or our company, you can phone us on XXXXX

END-OF-QUESTIONNAIRE
