# Supplementary material for: Implementation of the SunSmart program and population sun protection behaviour in Melbourne, Australia: Results from cross-sectional summer surveys from 1987 to 2017
Source: PLoS Med. 2019 Oct 8;16(10):e1002932. doi: 10.1371/journal.pmed.1002932 (PMC6782093; doi:10.1371/journal.pmed.1002932)
Supplement: S1 Table — (DOCX) [file pmed.1002932.s003.docx]

| **S1 Table:** Sun Protection Survey questions and response categories | |
| --- | --- |
| **Questionnaire item** | **Response categories** |
| ***Sunburn incidence*** |  |
| Did you get at all sunburnt yesterday? What about on Saturday? | 1. Sunday  2. Saturday  3. Neither day |
| ***Outdoors*** |  |
| Thinking back to Sunday. Were you out of doors for longer than 15 minutes between 11am and 3pm?  By out of doors we mean not in a building and not in a covered vehicle. | 1. Yes 2. No |
| ***Activity type*** |  |
| What activity were you doing mostly during that time out of doors? | *Verbatim response -* used in subsequent questions: (~100 activity categories) |
| ***Total time outdoors*** |  |
| About how much time in total did you spend out of doors on Sunday between 11am and 3pm [verbatim response - activity outdoors] in total? | *Continuous response:* total time in minutes |
| ***Activity in metropolitan area*** |  |
| Were you in the Melbourne metropolitan area when you were [*verbatim response - activity outdoors*]? | 1. Yes  2. No |
| ***Used shade*** |  |
| Were you mostly in the shade or mostly in the shade or mostly out in the open while you were [*verbatim response - activity outdoors*]? | 1. In the shade  2. In the open  3. In shade and out in open equally  4. Can’t say |
| *Now we need to ask you some questions in detail about what you were wearing yesterday (SUNDAY) to find out how much your skin was exposed to direct sunlight. Would you mind telling me what you were wearing while you were [verbatim response - activity outdoors]?* | |
| ***Cover on top part of body*** |  |
| Can you remember what you were wearing on the top part of your body? | 1. Top/dress/wetsuit  2. Swimwear  3. Topless |
| ***Cover on lower part of body*** |  |
| Can you remember what you were wearing on the lower part of your body? | 1.Trousers/jeans/shorts/skirt/dress/wetsuit  2. Swimwear  3. Bottomless |
| ***Sleeve length*** |  |
| How long were the sleeves of your top/dress/wetsuit? | 1. Wrist length  2. ¾ length  3. Elbow length  4. Short  5. Sleeveless |
| ***Bottoms length*** |  |
| How long were/was your trousers/jeans/shorts/skirt/dress/ wetsuit? | 1. Ankle length  2. ¾ length  3. Knee length  4. Mini skirt/short shorts |
| ***Swimwear*** |  |
| What sort of swimwear/bathers were you wearing? | 1. One piece bathers  2. Two piece/bikini  3. Bikini top only  4. Bikini bottom only  5. Long/board shorts  6. Short shorts  7. Briefs/speedos  8. Rash vest |
| ***Wore hat*** |  |
| Were you wearing a cap, hat or sun visor? | 1. Hat  2. Cap  3. Visor  4. None |
| ***Brim of hat*** |  |
| Did your #hat/cap/ have a wide brim or narrow brim? | 1. Wide brim  2. Narrow brim  3. No brim |
| ***Flap of hat*** |  |
| Did it have a flap which covered the back of your neck? | 1. Yes  2. No |
| ***Wore sunglasses*** |  |
| Were you wearing any sunglasses? | 1. Yes  2. No |
| *Now some questions about sunscreen. A sunscreen is a gel, lotion or cream that filters out ultraviolet sunlight to prevent burning and other skin damage.* | |
| ***Used sunscreen*** |  |
| Did you use a sunscreen between #/11am and 3pm/10am and 2pm/ on Sunday? | 1. Yes used sunscreen  2. No, didn’t use sunscreen  3. Makeup with a sunscreen only |
| What was the sun protection factor of the sunscreen you used? | *Continuous response:* SPF |
| On what parts of the body did you apply sunscreen? | 1. Face  2. Nose  3. Head  4. Ears  5. Chest  6. Stomach  7. Back  8. Neck  9. Shoulders  10. Arms  11. Hands  12. Legs  13. Back of knees  14. Feet |
| ***Like to get a suntan*** |  |
| Do you like to get a suntan or not? | 1. Yes  2. No |
| ***A suntanned person is more healthy*** |  |
| ‘A suntanned person is more healthy’ | 1. Strongly agree  2. Mildly agree  3. Neither agree nor disagree  4. Mildly disagree  5. Strongly disagree  6. Can’t say |
| ***Most friends think a suntan is a good thing*** |  |
| ‘Most of my friends think a suntan is a good thing’ | 1. Strongly agree  2. Mildly agree  3. Neither agree nor disagree  4. Mildly disagree  5. Strongly disagree  6. Can’t say |
| **Note:** All questions were repeated for Saturday. | |
