# Supplementary material for: Implementation of the SunSmart program and population sun protection behaviour in Melbourne, Australia: Results from cross-sectional summer surveys from 1987 to 2017
Source: PLoS Med. 2019 Oct 8;16(10):e1002932. doi: 10.1371/journal.pmed.1002932 (PMC6782093; doi:10.1371/journal.pmed.1002932)
Supplement: S2 Table — (DOCX) [file pmed.1002932.s004.docx]

| **S2 Table**: Tanning attitudes and sun protection behaviour among respondents outdoors (1987-88 to 2010s) | | | | |  |
| --- | --- | --- | --- | --- | --- |
|  | **Decade** (Adjusted %) | | | |  |
|  | **1987-88**  *Pre-SunSmart*  (n=1,655) | **1990s**  (n=5,258) | **2000s**  (n=3,385) | **2010s**  (n=2,987) | |
| **Tanning attitudes** | (n=1,655) | (n=5,258) | (n=3,385) | (n=2,987) | |
| Do you like to get a suntan? (no) | 43.1 | 65.0 | 59.7 | 66.1 | |
| Thinks a suntan is healthy (disagree) | 82.3 | 90.3 | 88.3 | 89.8 | |
| Friends think a suntan is good (disagree) | 35.9 | 63.1 | 57.0 | 56.5 | |
| **Exposure to ultraviolet radiation** | (n=1,501) | (n=4,752) | (n=2,940) | (n=2,623) | |
| Outdoors on Sunday (yes) | 55.5 | 47.7 | 46.0 | 41.8 | |
| Time outdoors^a^ (adjusted mean, minutes (SE)) | 120 (2.7) | 118 (1.5) | 111 (1.9) | 101 (2.0) | |
| **Sun protection behaviour^b^** | (n=1,064) | (n=2,984) | (n=1,802) | (n=1,587) | |
| Used a hat | 19.7 | 39.2 | 38.9 | 36.5 | |
| Wore ¾ or long-sleeved top ^a^ | 15.9 | 20.3 | 19.4 | 21.5 | |
| Used sunscreen | 11.4 | 28.5 | 29.3 | 36.3 | |
| Stayed mostly in the shade | 19.3 | 25.7 | 25.3 | 28.2 | |
| Used at least one sun protection behaviour | 39.3 | 65.3 | 65.1 | 68.1 | |
|  | (n=1,457) | (n=4,612) | (n=2,785) | (n=2,496) | |
| **Maximal protection** |  |  |  |  | |
| Indoors on both days of the weekend^a^ | 27.9 | 35.6 | 35.4 | 37.5 | |
| Maximally protected both days excluding shade | 27.9 | 37.4 | 36.8 | 39.1 | |
| Maximally protected both days including shade | 38.7 | 50.7 | 50.0 | 53.4 | |
| **Weekend sunburn** | 11.4 | 8.2 | 8.5 | 7.6 | |
| Note: Bold face indicates statistical significance at *p*<0.05 level.  ^a^ Models not reported in main tables.  ^b^ Models include respondents who were outdoors on Sunday (or Saturday, if not outdoors on Sunday) in a metropolitan area. | | | | | |
