# Supplementary material for: Implementation of the SunSmart program and population sun protection behaviour in Melbourne, Australia: Results from cross-sectional summer surveys from 1987 to 2017
Source: PLoS Med. 2019 Oct 8;16(10):e1002932. doi: 10.1371/journal.pmed.1002932 (PMC6782093; doi:10.1371/journal.pmed.1002932)
Supplement: S3 Table — (DOCX) [file pmed.1002932.s005.docx]

| **S3 Table:** Mean change in the proportion of the body protected by sunscreen, clothing or a hat when outdoors per decade: relative to the pre-SunSmart program decade | | | | |
| --- | --- | --- | --- | --- |
|  | **Decade** | | | |
|  | Adjusted mean (S.E.) | | | |
|  | **1987-88**  *Pre-SunSmart*  (n=1,064) | **1990s**  (n=2,984) | **2000s**  (n=1,802) | **2010s**  (n=1,587) |
| **Body Cover Index^a^** | 0.72 (0.70-0.73) | **0.79 (0.79, 0.80)** | **0.79 (0.78, 0.80)** | **0.80 (0.79, 0.81)** |
|  |  |  |  |  |
| Note: Bold face indicates statistical significance at *p*<0.05 level.  The multiple linear regression model included respondents outdoors in the Melbourne metropolitan area during peak UV hours (11am to 3pm). The mean and S.E. are reported adjusting for covariates: age, sex, skin sensitivity, survey month, weekend temperature, weekend cloud cover, median monthly temperature, no. days heavy cloud cover (≥ 6 oktas) past month, no. days heavy rainfall (≥1mm) past month.   ^a^ The ‘Body Cover Index’ describes the proportion of the body covered by a hat, sunglasses, clothing and/or sunscreen on a scale of zero (full exposure) to 1 (full coverage). | | | | |
